# Supplementary material for: New Imatinib Derivatives with Antiproliferative Activity against A549 and K562 Cancer Cells
Source: Molecules. 2022 Jan 24;27(3):750. doi: 10.3390/molecules27030750 (PMC8838532; doi:10.3390/molecules27030750)
Supplement: Supplementary file 1 [file molecules-27-00750-s001.zip › molecules-1555206-supplementary.pdf]

New imatinib derivatives with antiproliferative activity against A549 and K562 cancer cells

Andressa Oliveira[1,2], Stefany Moura[1,2], Luiz Pimentel[1], João Neto[3], Rafael Dantas[3], Floriano Junior[2,3], Monica Bastos[1,2] and Nubia Boechat*[1,2]

[1] Laboratório de Sintese de Farmacos - LASFAR, Instituto de Tecnologia em Farmacos - Farmanguinhos, FIOCRUZ, Rua Sizenando Nabuco 100, Manguinhos, Rio de Janeiro, RJ, 21041-250, Brazil.
[2] Programa de Pós-graduação em Farmacologia e Química Medicinal do Instituto de Ciências Biomédicas – ICB-UFRJ, Centro de Ciências da Saúde - CCS, Bloco J, Ilha do Fundão, Rio de Janeiro, RJ, 21941-902, Brazil.
[3] Fundação Oswaldo Cruz, Instituto Oswaldo Cruz, Laboratório de Bioquímica Experimental e Computacional de Fármacos, Av. Brasil 4365, Manguinhos, Rio de Janeiro, RJ, 21040-360, Brazil.
* Nubia Boechat, LASFAR, Farmanguinhos, FIOCRUZ, Rio de Janeiro/ PPGFQM - ICB- UFRJ, RJ, Brazil; https://orcid.org/0000-0003-0146-2218, Email: nboechat@gmail.com

Table of contents:

Compound **10**.. Page 2
Compounds **12a-e**.. Page 3-7
Compounds **2a-e**.. Page 8-23
Compounds **13a-e**.. Page 24-33
Compounds **14a-e**.. Page 34-42
Compounds **3a-e**.. Page 43-62

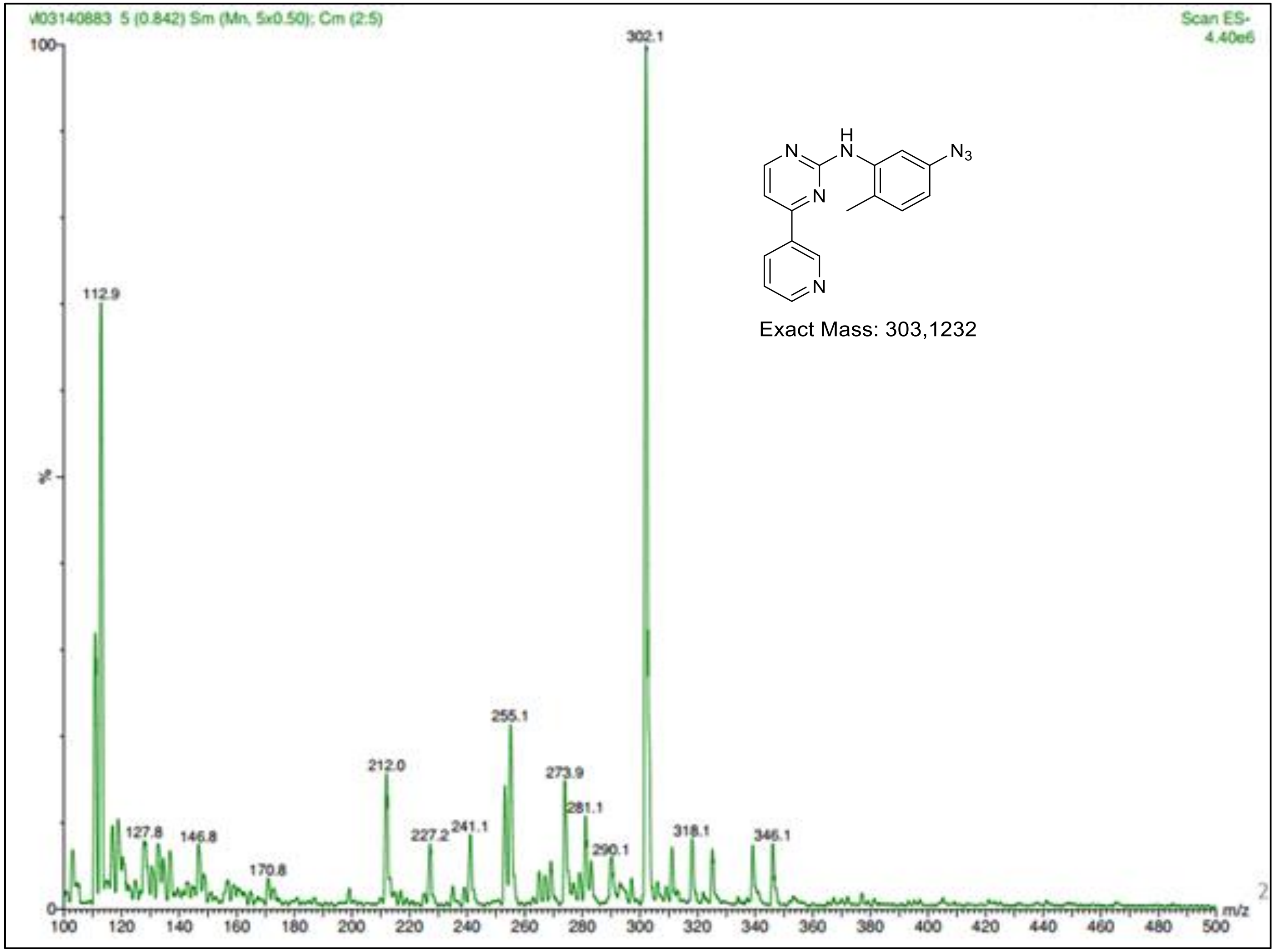

Figure S1. ESI-MS of N-(5-azido-2-methylphenyl)-4-(pyridin-3-yl)pyrimidin-2-amine (**10**)

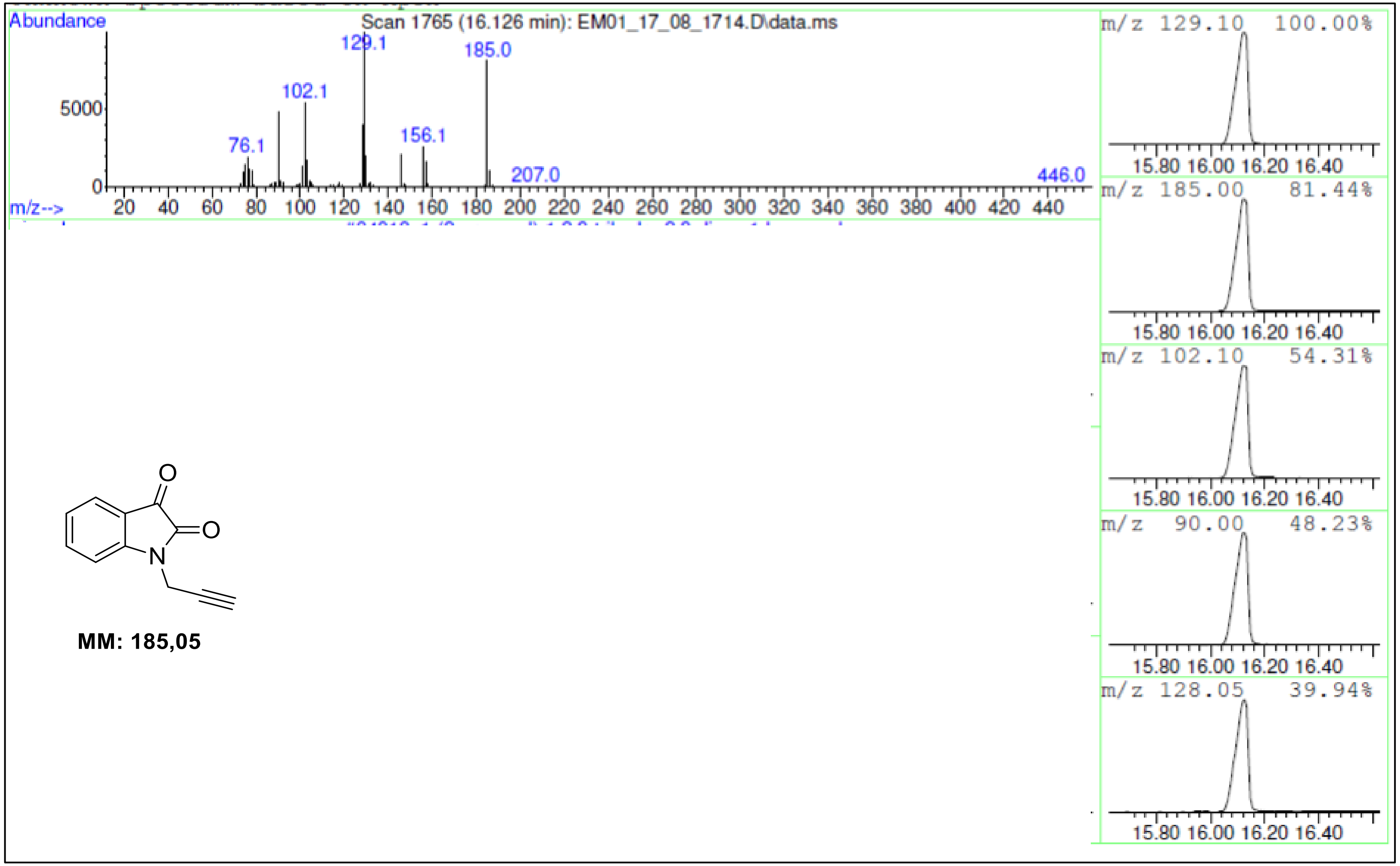

Figure S2. GC-MS of 1-(prop-2-yn-1-yl)indoline-2,3-dione (**12a**)

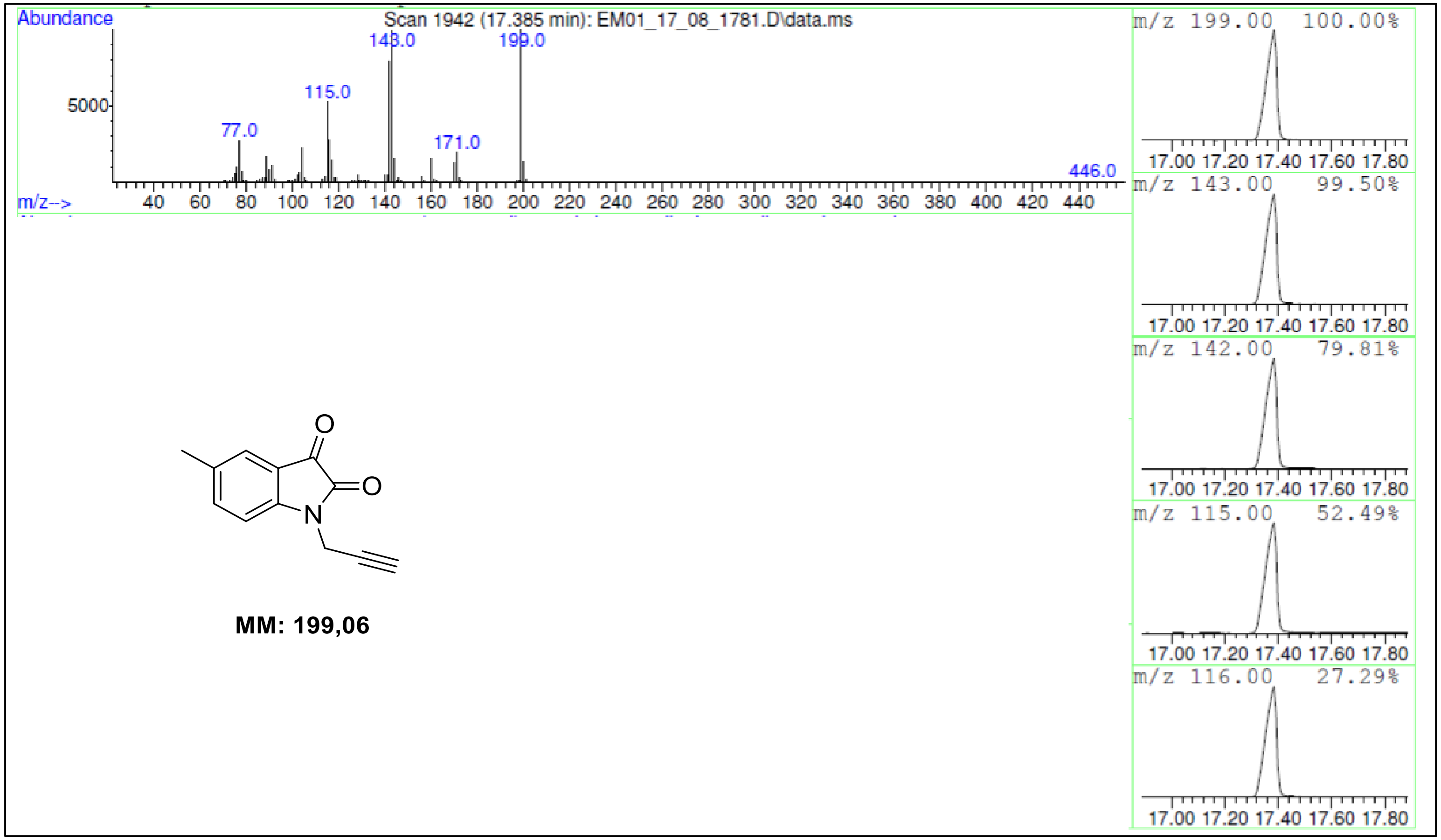

Figure S3. GC-MS of 5-methyl-1-(prop-2-yn-1-yl)indoline-2,3-dione (**12b**)

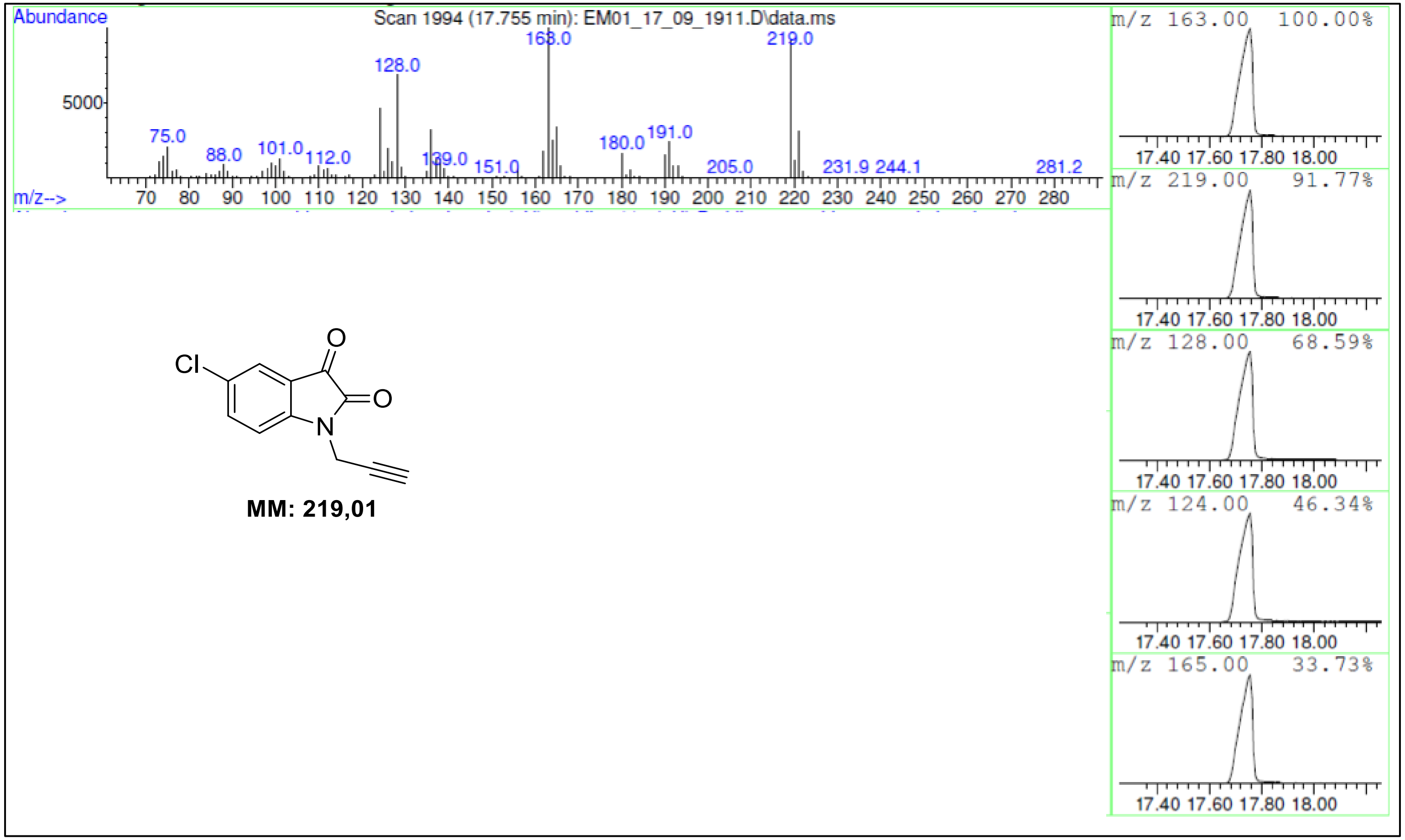

Figure S4. GC-MS of 5-chloro-1-(prop-2-yn-1-yl)indoline-2,3-dione (**12c**)

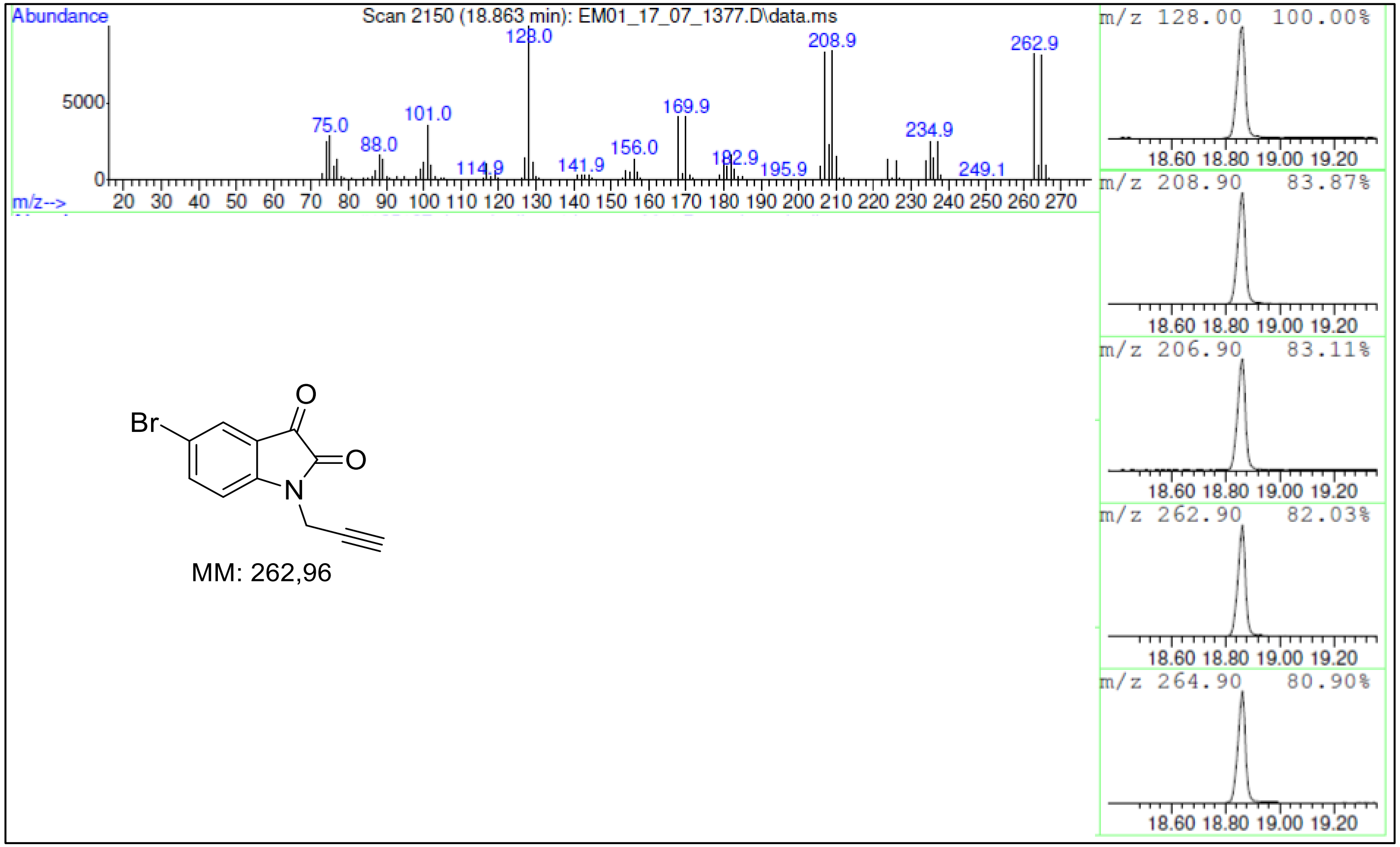

Figure S5. GC-MS of 5-bromo-1-(prop-2-yn-1-yl)indoline-2,3-dione (**12d**)

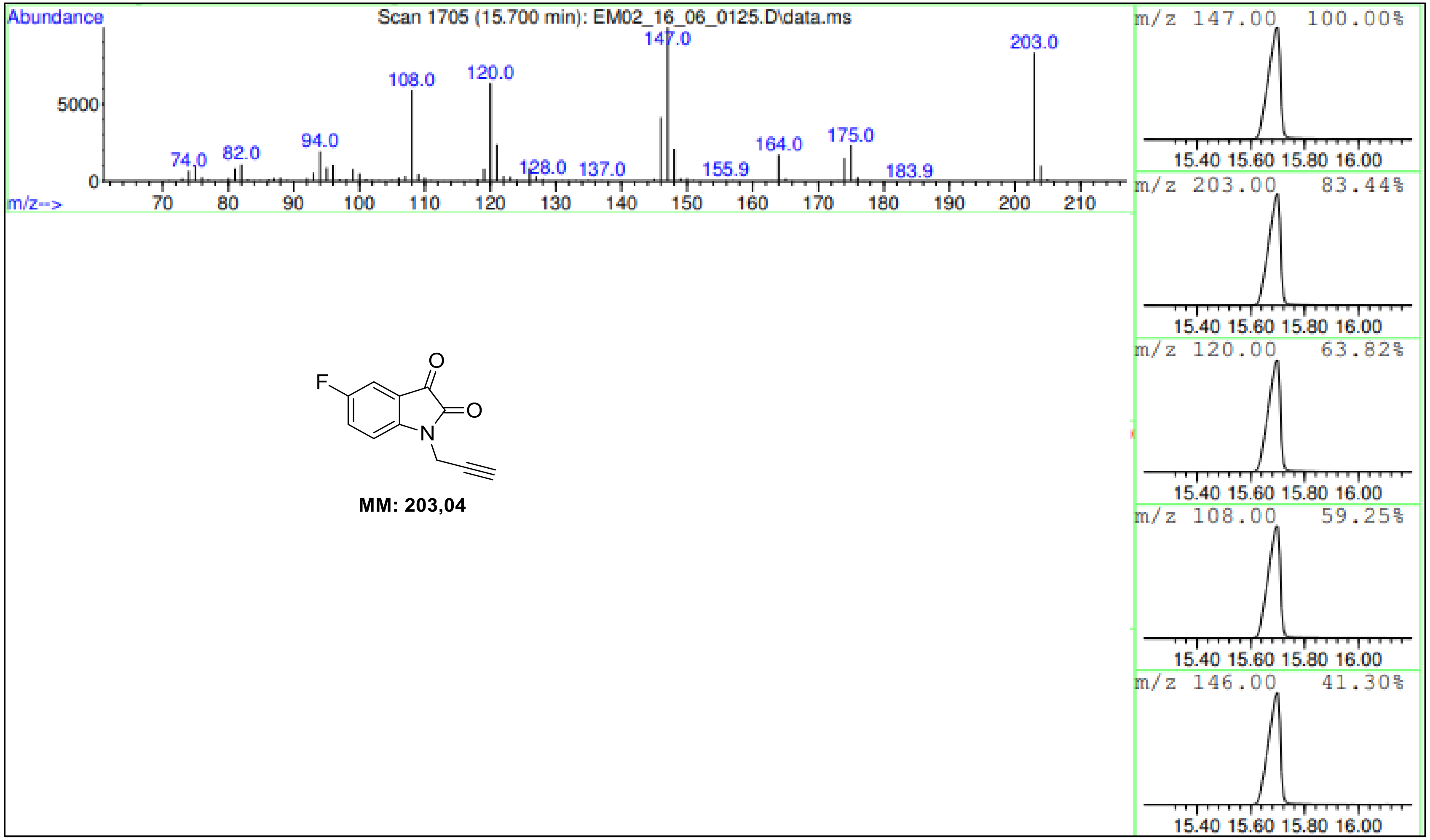

Figure S6. GC-MS of 5-fluoro-1-(prop-2-yn-1-yl)indoline-2,3-dione (**12e**)

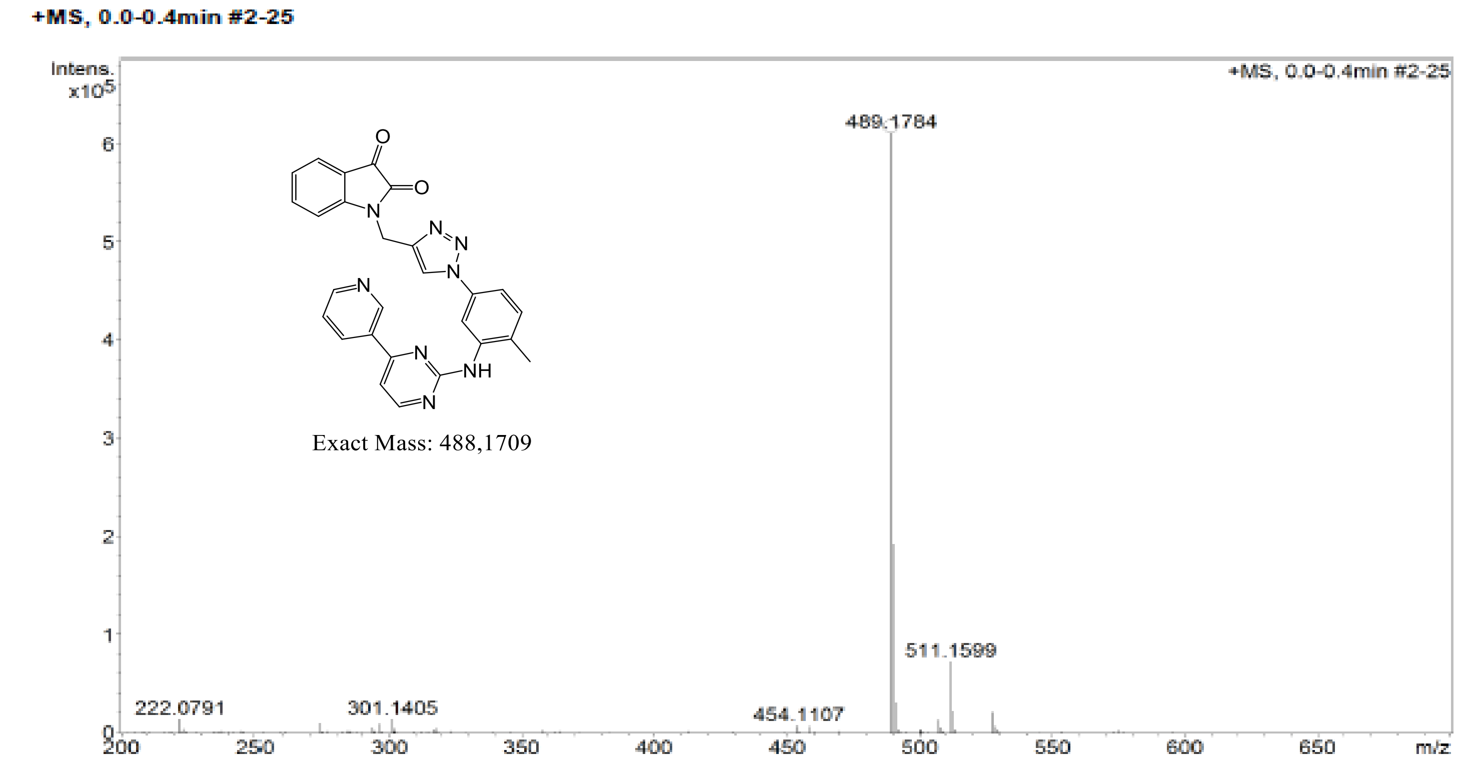

Meas. m/z	#	Ion Formula	m/z	err [ppm]	Mean err [ppm]	rdb	N-Rule	e⁻ Conf	mSigma	Std I	Std Mean m/z	Std I VarNorm	Std m/z Diff	Std Comb Dev
489.178367	1	C27H21N6O2	489.178198	-0.3	0.0	21.5	ok	even	6.0	9.1	n.a.	n.a.	n.a.	n.a.
	2	C26H25N4O6	489.176861	-3.1	-1.9	16.5	ok	even	7.6	10.8	n.a.	n.a.	n.a.	n.a.
	3	C23H17N14	489.175513	-5.8	-6.1	22.5	ok	even	8.5	13.2	n.a.	n.a.	n.a.	n.a.
	4	C31H25N2O4	489.180684	5.1	6.8	20.5	ok	even	17.9	31.3	n.a.	n.a.	n.a.	n.a.
	5	C25H29O10	489.175524	-5.8	-4.4	11.5	ok	even	19.1	28.4	n.a.	n.a.	n.a.	n.a.
	6	C32H21N6	489.182221	7.9	9.3	25.5	ok	even	28.8	46.4	n.a.	n.a.	n.a.	n.a.

Figure S7. HRMS of 1-((1-(4-methyl-3-((4-(pyridin-3-yl)pyrimidin-2-yl)amino)phenyl)-1H-1,2,3-triazol-4-yl)methyl)indoline-2,3-dione (**2a**)

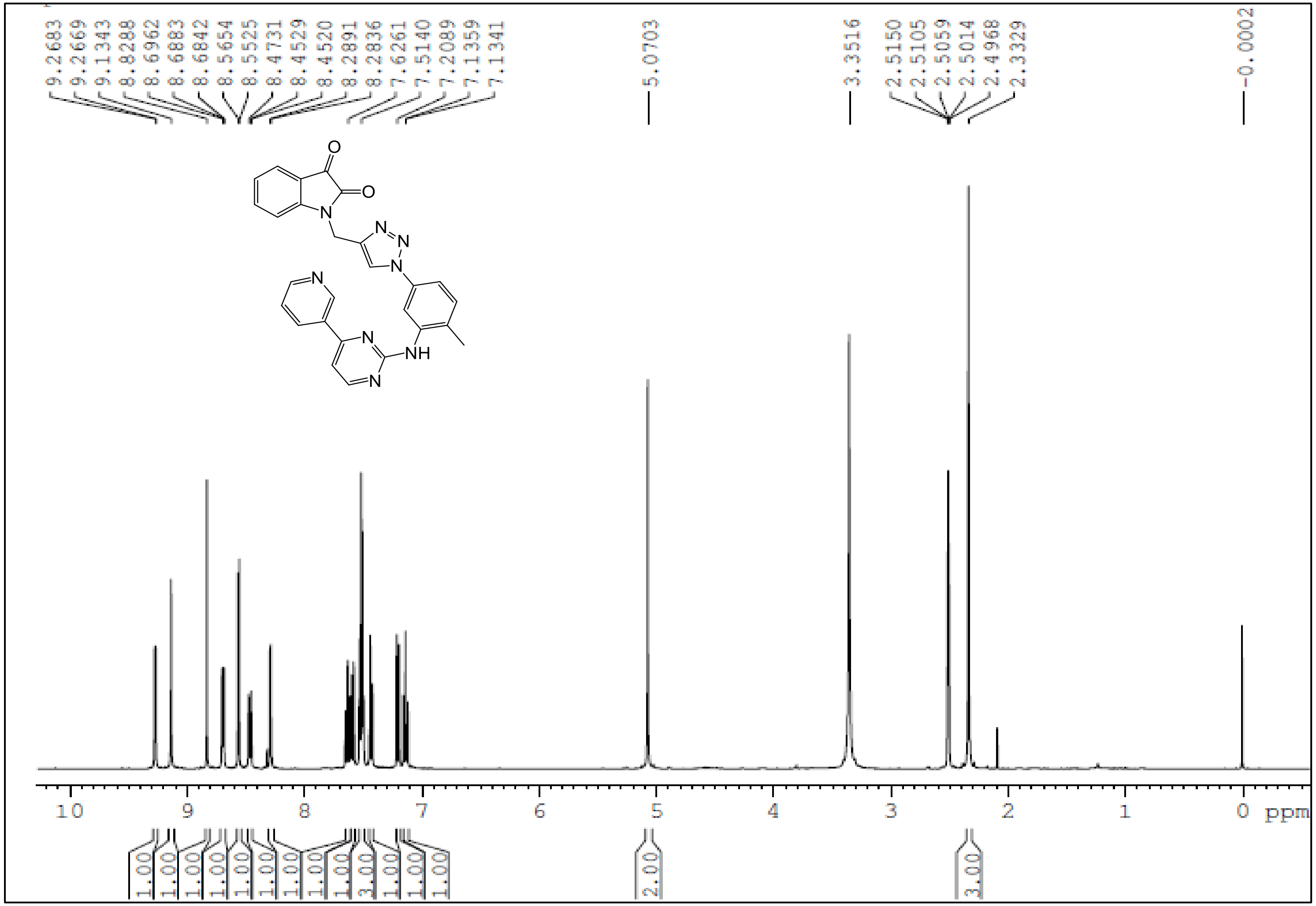

Figure S8. ^{1}H NMR of 1-((1-(4-methyl-3-((4-(pyridin-3-yl)pyrimidin-2-yl)amino)phenyl)-1H-1,2,3-triazol-4-yl)methyl)indoline-2,3-dione (**2a**)

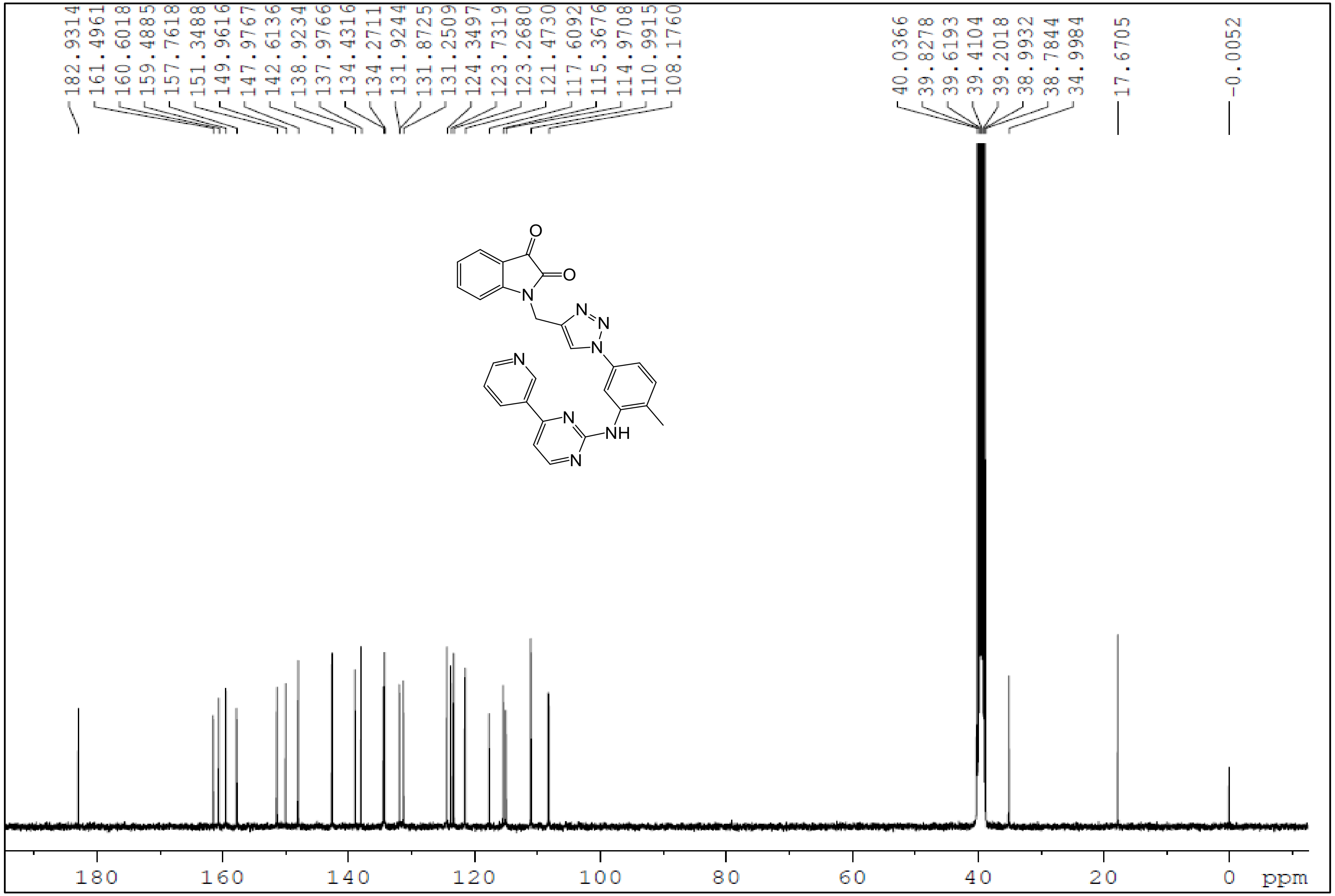

Figure S9. ^{13}C NMR of 1-((1-(4-methyl-3-((4-(pyridin-3-yl)pyrimidin-2-yl)amino)phenyl)-1H-1,2,3-triazol-4-yl)methyl)indoline-2,3-dione (**2a**)

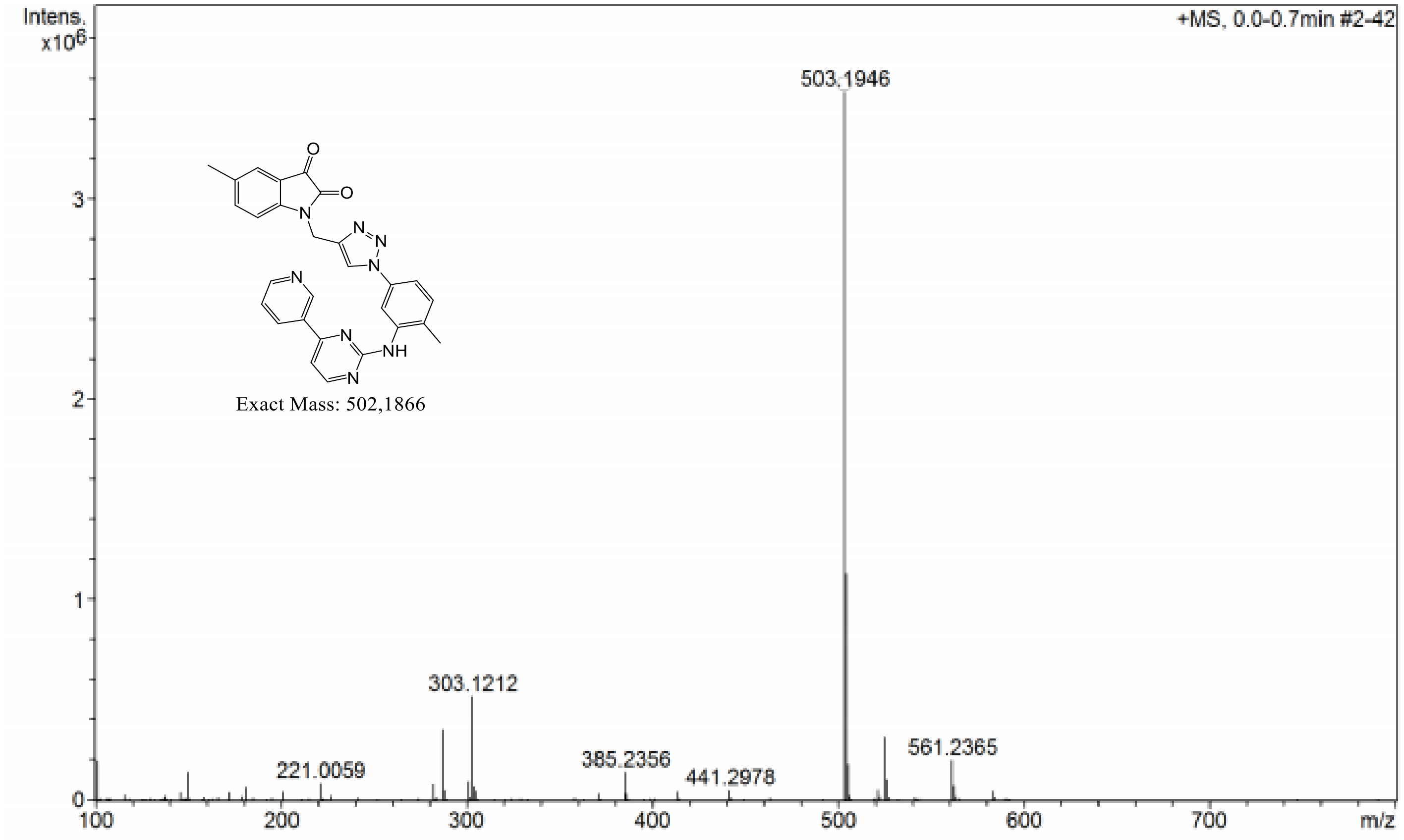

Meas. m/z	#	Ion Formula	m/z	err [ppm]	Mean err [ppm]	rdb	N-Rule	e⁻ Conf	mSigma	Std I	Std Mean m/z	Std I VarNorm	Std m/z Diff	Std Comb Dev
503.194614	1	C27H27N4O6	503.192511	-4.2	-3.1	16.5	ok	even	6.3	9.4	n.a.	n.a.	n.a.	n.a.
	2	C28H23N8O2	503.193848	-1.5	-0.7	21.5	ok	even	8.3	14.6	n.a.	n.a.	n.a.	n.a.
	3	C32H27N2O4	503.196534	3.8	5.3	20.5	ok	even	21.1	36.6	n.a.	n.a.	n.a.	n.a.

Figure S10. HRMS of 5-methyl-1-((1-(4-methyl-3-((4-(pyridin-3-yl)pyrimidin-2-yl)amino)phenyl)-1H-1,2,3-triazol-4-yl)methyl)indoline-2,3-dione (**2b**)

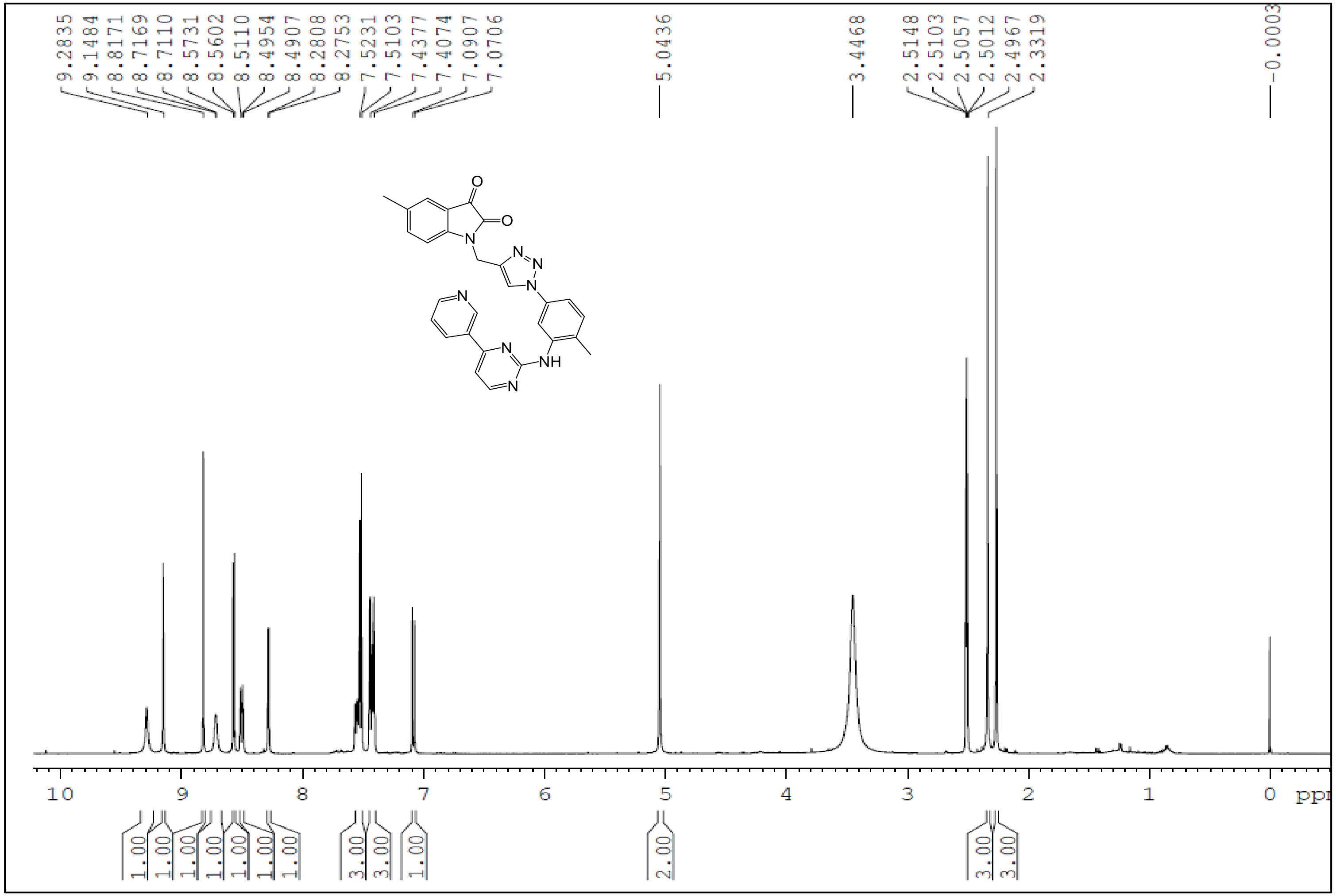

Figure S11. ^{1}H NMR of 5-methyl-1-((1-(4-methyl-3-((4-(pyridin-3-yl)pyrimidin-2-yl)amino)phenyl)-1H-1,2,3-triazol-4-yl)methyl)indoline-2,3-dione (**2b**)

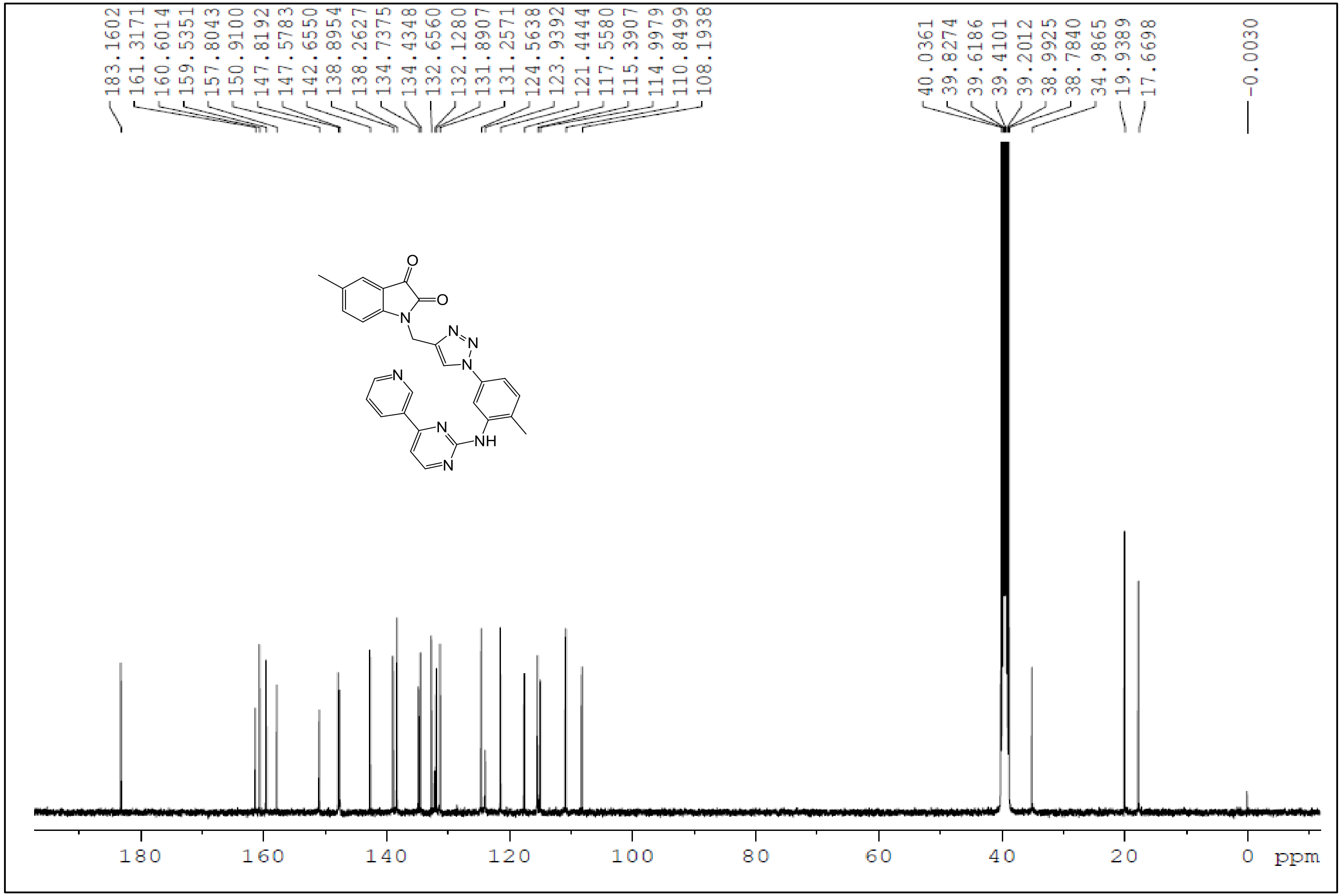

Figure S12. ^{13}C NMR of 5-methyl-1-((1-(4-methyl-3-((4-(pyridin-3-yl)pyrimidin-2-yl)amino)phenyl)-1H-1,2,3-triazol-4-yl)methyl)indoline-2,3-dione (**2b**)

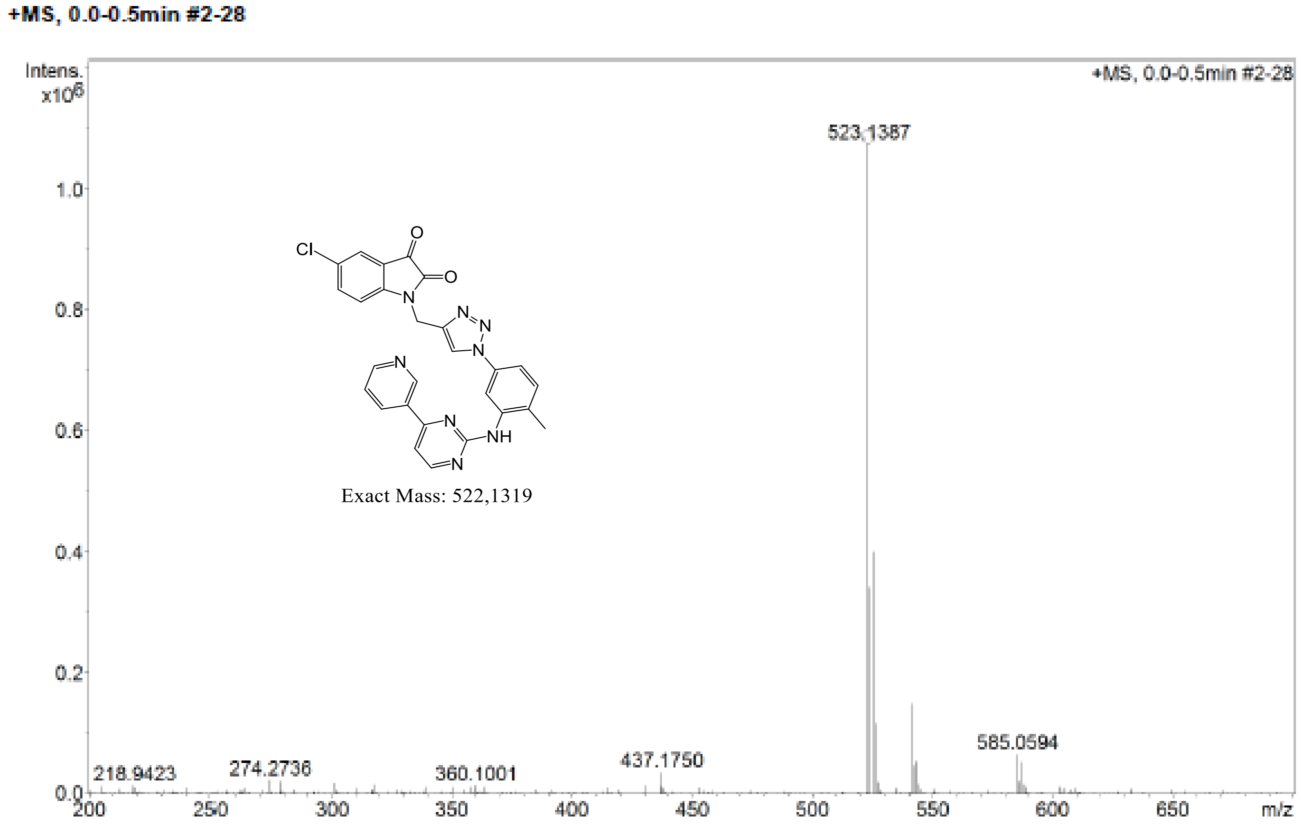

Meas. m/z	#	Ion Formula	m/z	err [ppm]	Mean err [ppm]	rdb	N-Rule	e⁻ Conf	mSigma	Std I	Std Mean m/z	Std I VarNorm	Std m/z Diff	Std Comb Dev
523.138692	1	C27H20ClN8O2	523.139226	1.0	0.9	21.5	ok	even	3.1	4.1	n.a.	n.a.	n.a.	n.a.
	2	C26H24ClN4O6	523.137889	-1.5	-1.4	16.5	ok	even	8.1	10.4	n.a.	n.a.	n.a.	n.a.
	3	C23H16ClN14	523.136541	-4.1	-5.0	22.5	ok	even	9.1	13.0	n.a.	n.a.	n.a.	n.a.
	4	C25H28ClO10	523.136551	-4.1	-3.6	11.5	ok	even	18.9	24.9	n.a.	n.a.	n.a.	n.a.

Figure S13. HRMS of 5-chloro-1-((1-(4-methyl-3-((4-(pyridin-3-yl)pyrimidin-2-yl)amino)phenyl)-1H-1,2,3-triazol-4-yl)methyl)indoline-2,3-dione (**2c**)

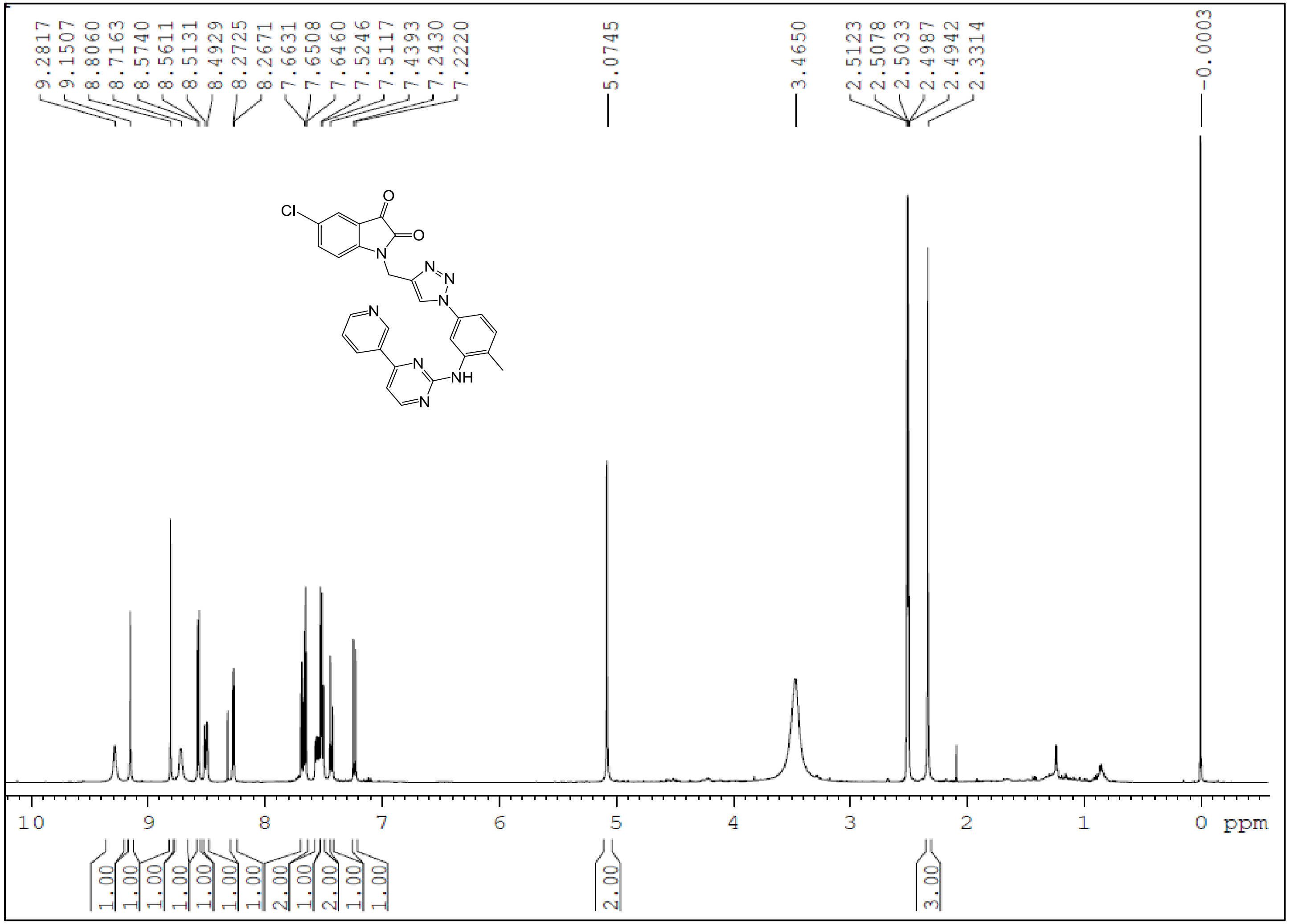

Figure S14. ^{1}H NMR of 5-chloro-1-((1-(4-methyl-3-((4-(pyridin-3-yl)pyrimidin-2-yl)amino)phenyl)-1H-1,2,3-triazol-4-yl)methyl)indoline-2,3-dione (**2c**)

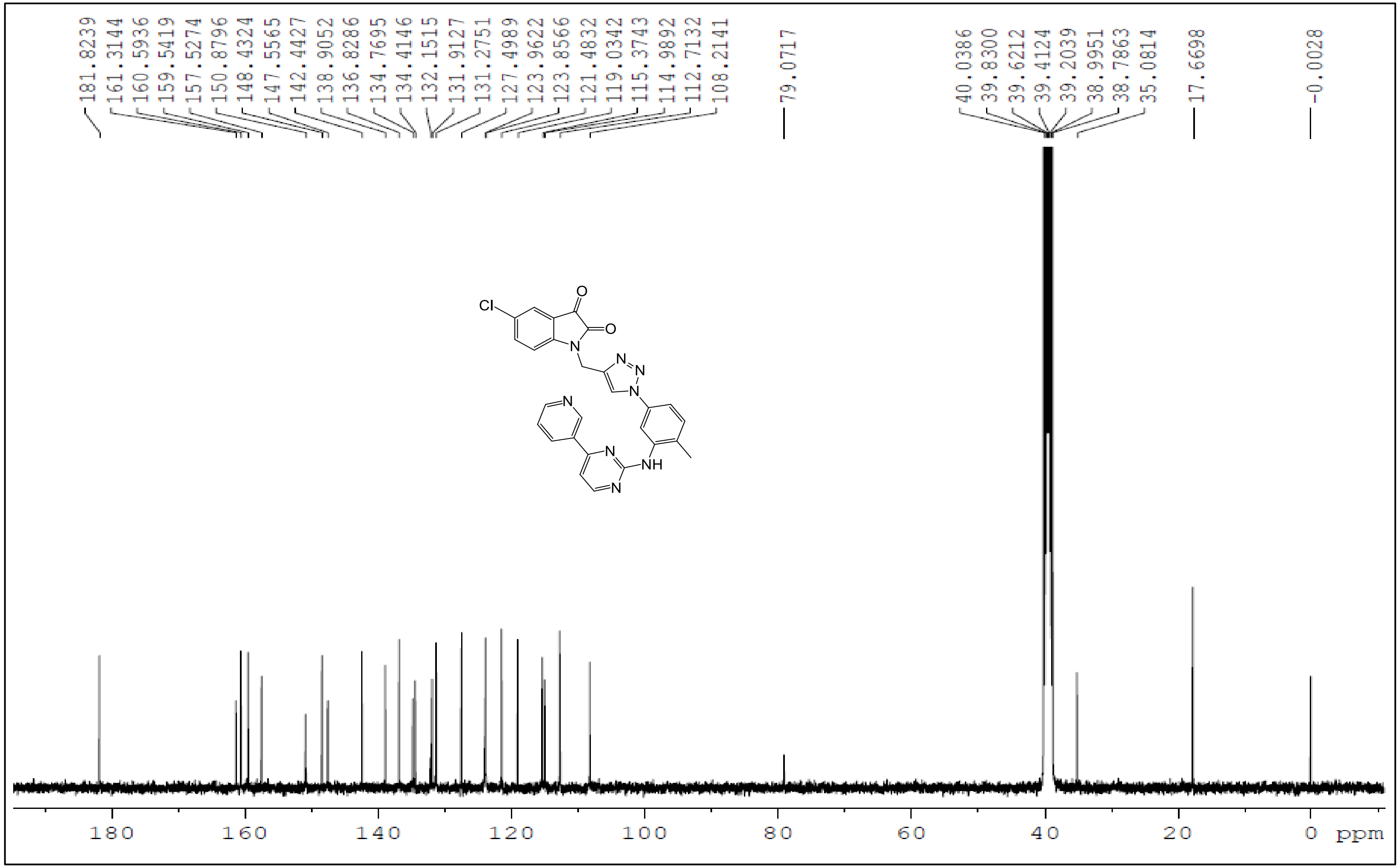

Figure S15. ^{13}C NMR of 5-chloro-1-((1-(4-methyl-3-((4-(pyridin-3-yl)pyrimidin-2-yl)amino)phenyl)-1H-1,2,3-triazol-4-yl)methyl)indoline-2,3-dione (**2c**)

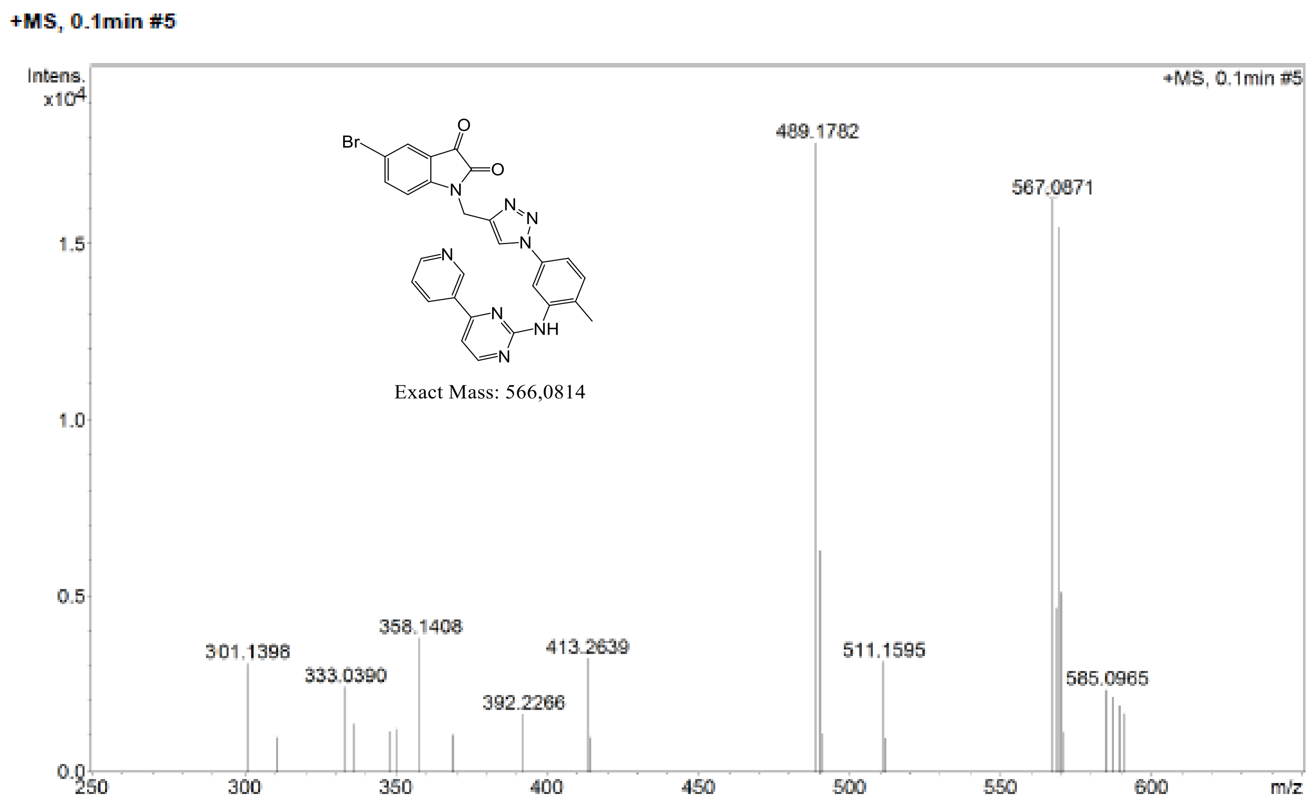

Meas. m/z	#	Ion Formula	m/z	err [ppm]	Mean err [ppm]	rdb	N-Rule	e⁻ Conf	mSigma	Std I	Std Mean m/z	Std I VarNorm	Std m/z Diff	Std Comb Dev
567.087086	1	C23H16BrN14	567.086025	-1.9	804.4	22.5	ok	even	31.5	27.0	n.a.	n.a.	n.a.	n.a.
	2	C26H24BrN4O6	567.087373	0.5	818.5	16.5	ok	even	36.1	32.1	n.a.	n.a.	n.a.	n.a.
	3	C27H20BrN8O2	567.088710	2.9	814.1	21.5	ok	even	36.3	33.2	n.a.	n.a.	n.a.	n.a.
	4	C22H20BrN10O4	567.084688	-4.2	809.4	17.5	ok	even	36.5	31.7	n.a.	n.a.	n.a.	n.a.
	5	C25H28BrO10	567.086036	-1.9	823.5	11.5	ok	even	41.3	36.2	n.a.	n.a.	n.a.	n.a.
	6	C21H24BrN6O6	567.083350	-6.6	814.8	12.5	ok	even	45.9	40.9	n.a.	n.a.	n.a.	n.a.

Figure S16. HRMS of 5-bromo-1-((1-(4-methyl-3-((4-(pyridin-3-yl)pyrimidin-2-yl)amino)phenyl)-1H-1,2,3-triazol-4-yl)methyl)indoline-2,3-dione (**2d**)

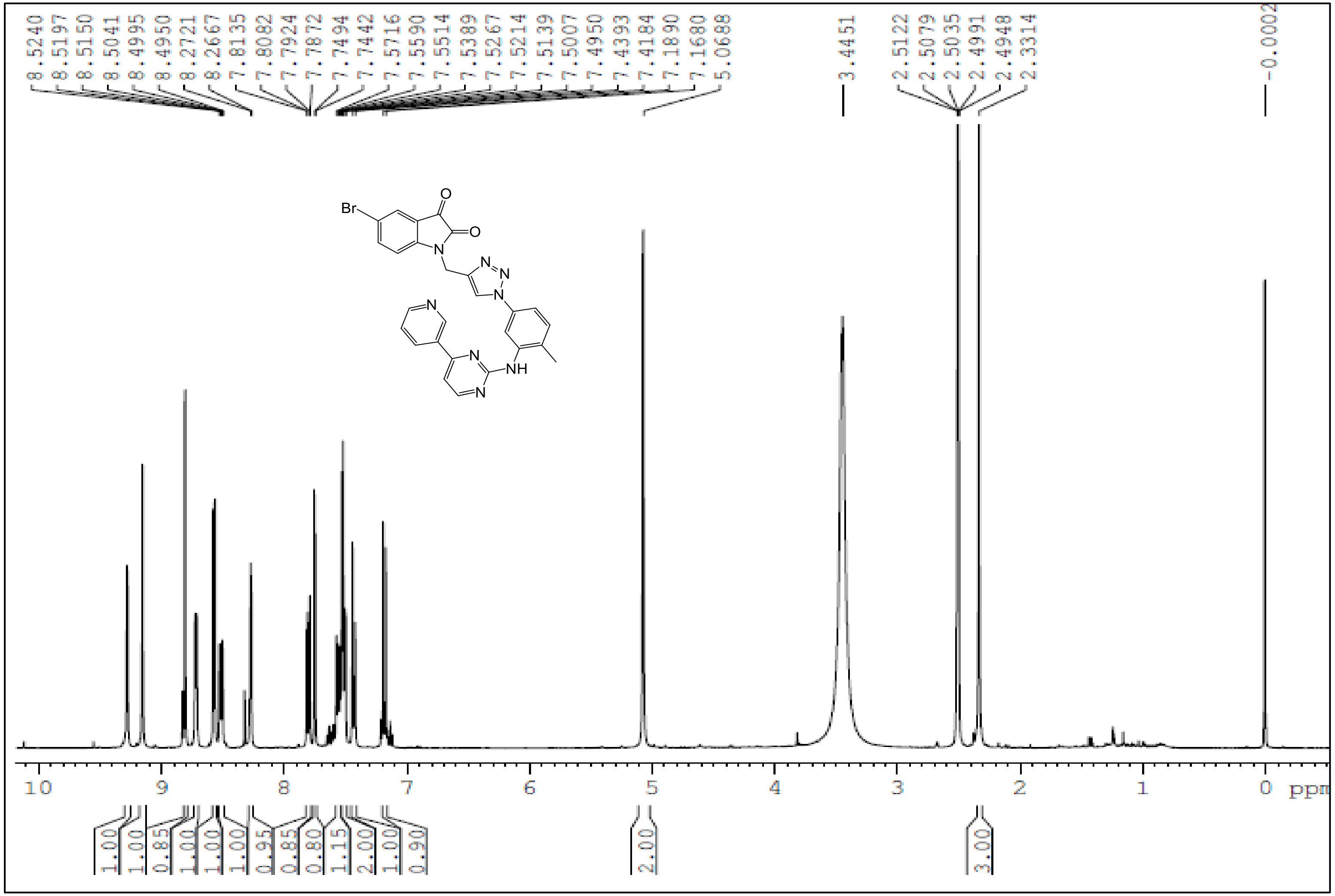

Figure S17. ^{1}H NMR of 5-bromo-1-((1-(4-methyl-3-((4-(pyridin-3-yl)pyrimidin-2-yl)amino)phenyl)-1H-1,2,3-triazol-4-yl)methyl)indoline-2,3-dione (**2d**)

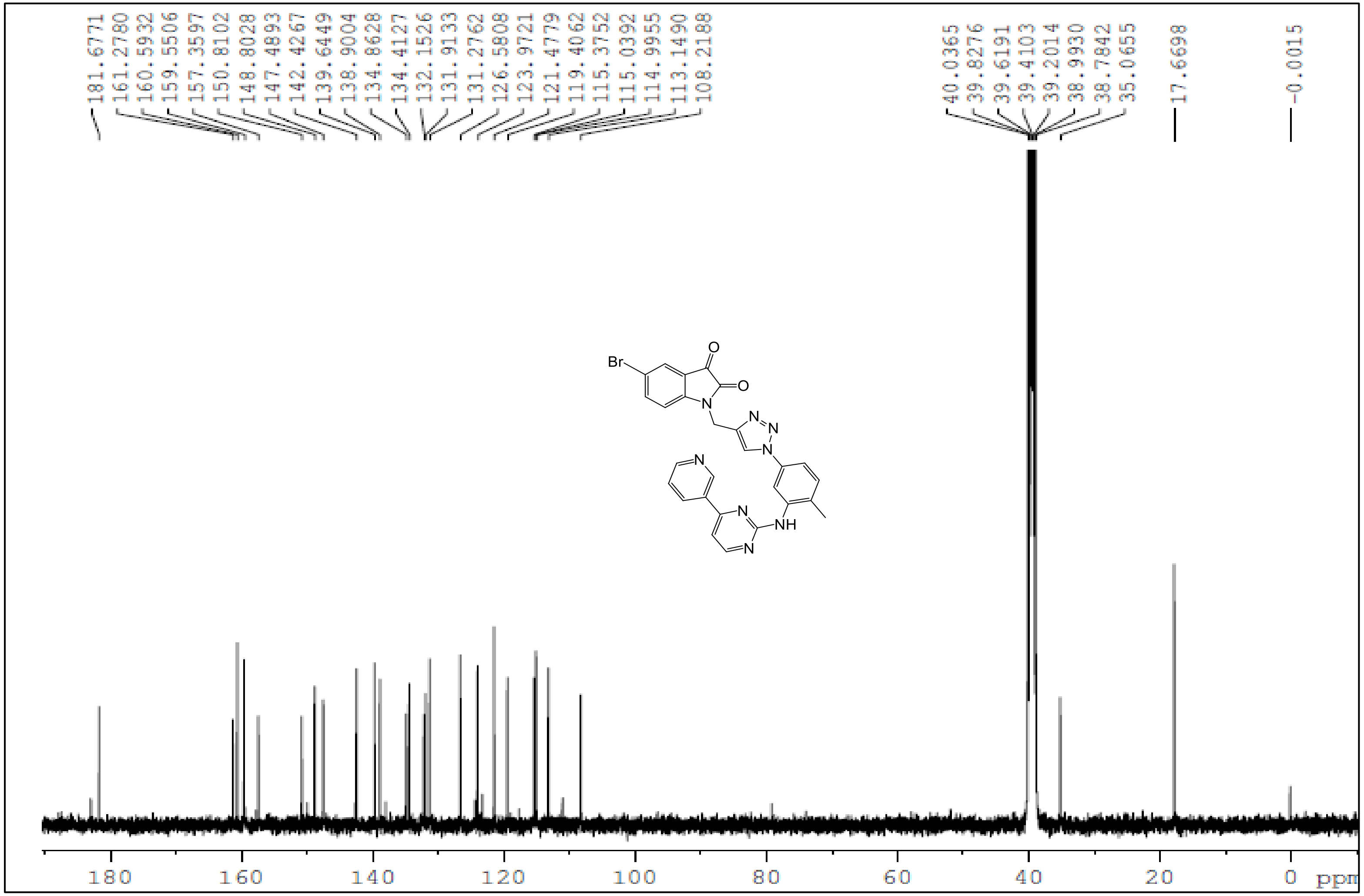

Figure S18. ^{13}C NMR of 5-bromo-1-((1-(4-methyl-3-((4-(pyridin-3-yl)pyrimidin-2-yl)amino)phenyl)-1H-1,2,3-triazol-4-yl)methyl)indoline-2,3-dione (**2d**)

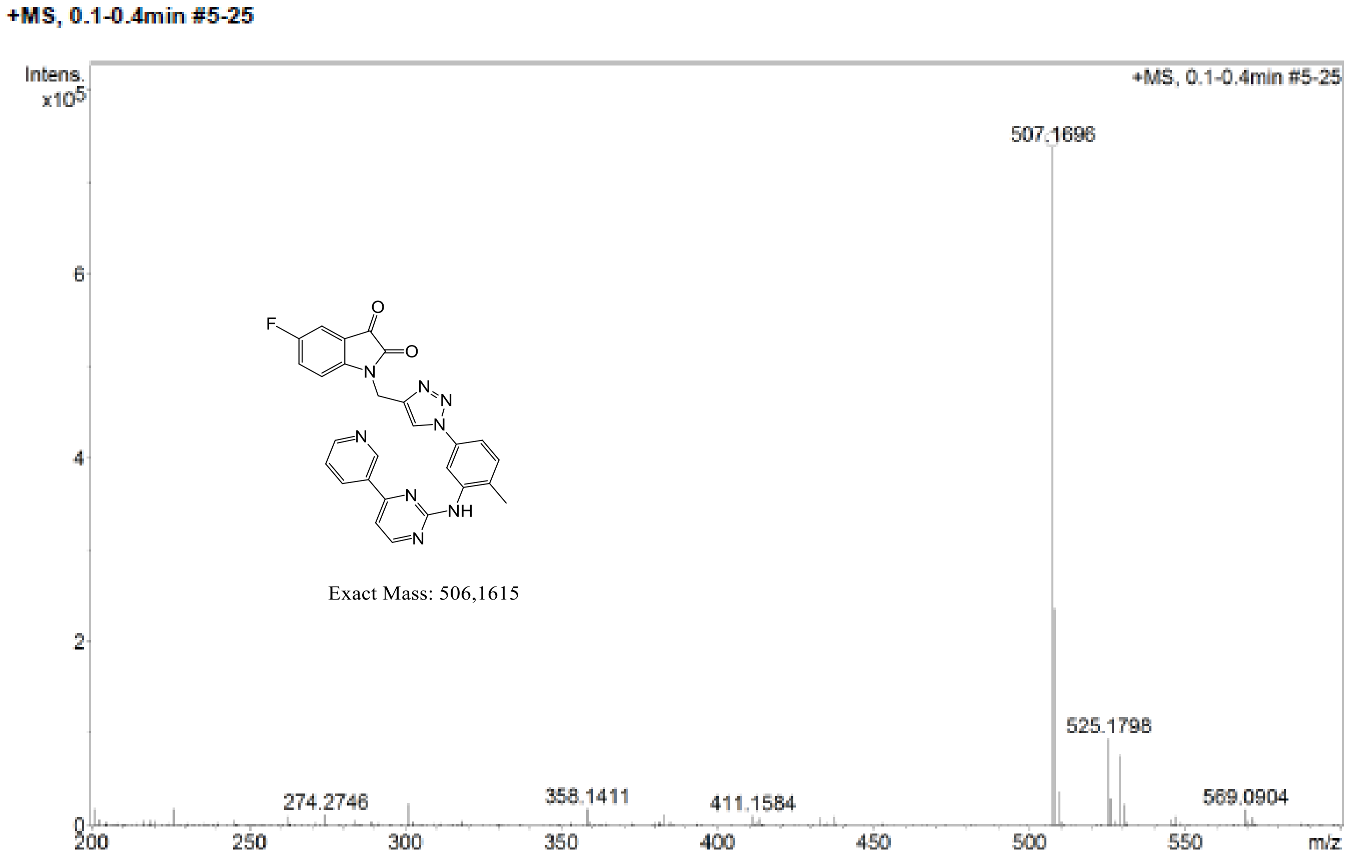

Meas. m/z	#	Ion Formula	m/z	err [ppm]	Mean err [ppm]	rdb	N-Rule	e⁻ Conf	mSigma	Std I	Std Mean m/z	Std I VarNorm	Std m/z Diff	Std Comb Dev
507.169565	1	C27H20FN8O2	507.168777	-1.6	-1.1	21.5	ok	even	3.5	5.6	n.a.	n.a.	n.a.	n.a.
	2	C26H24FN4O6	507.167439	-4.2	-3.1	16.5	ok	even	10.2	14.7	n.a.	n.a.	n.a.	n.a.
	3	C31H24FN2O4	507.171462	3.7	5.3	20.5	ok	even	15.5	27.6	n.a.	n.a.	n.a.	n.a.

Figure S19. HRMS of 5-fluoro-1-((1-(4-methyl-3-((4-(pyridin-3-yl)pyrimidin-2-yl)amino)phenyl)-1H-1,2,3-triazol-4-yl)methyl)indoline-2,3-dione (**2e**)

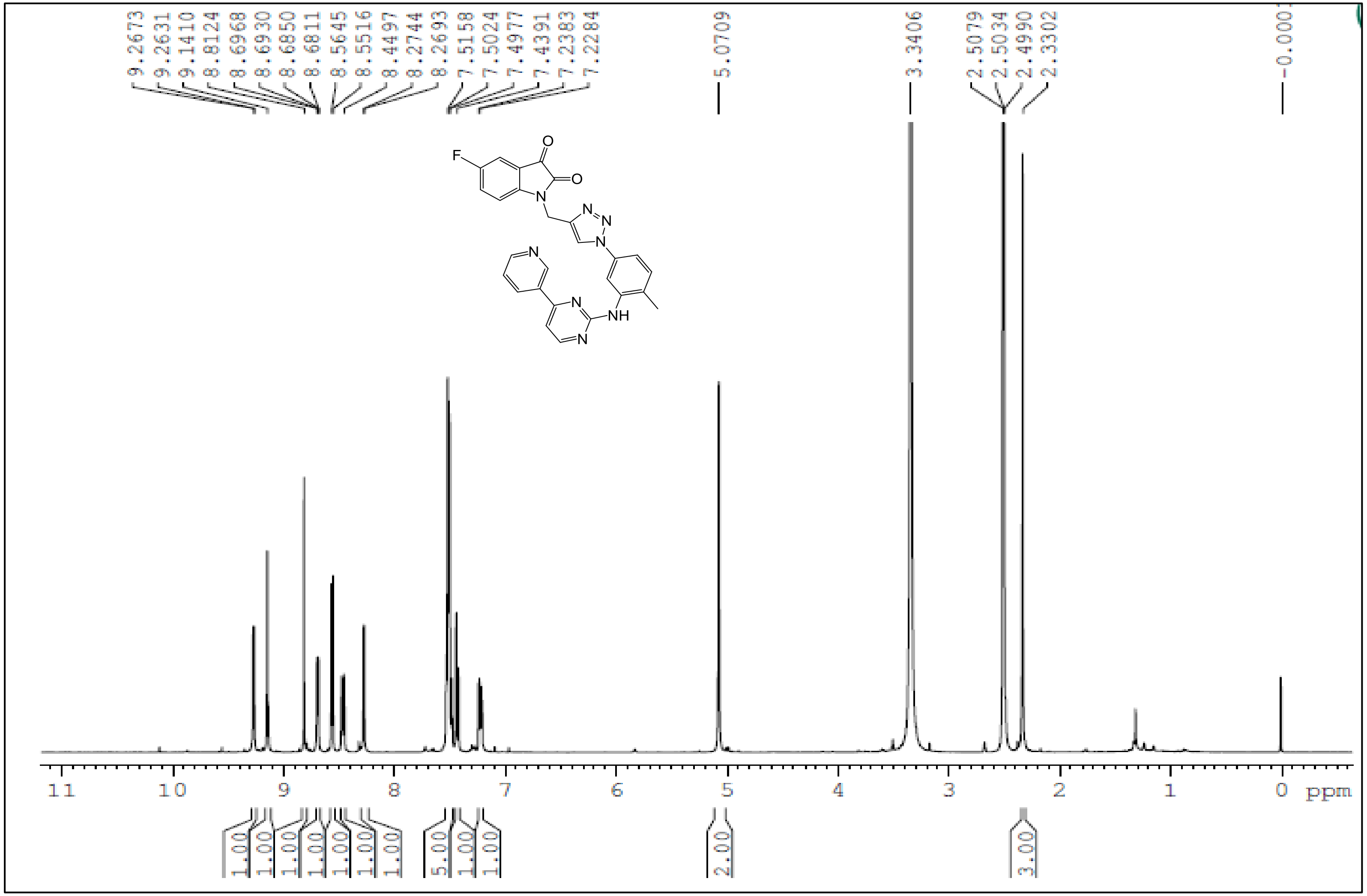

Figure S20. ^{1}H NMR of 5-fluoro-1-((1-(4-methyl-3-((4-(pyridin-3-yl)pyrimidin-2-yl)amino)phenyl)-1H-1,2,3-triazol-4-yl)methyl)indoline-2,3-dione (**2e**)

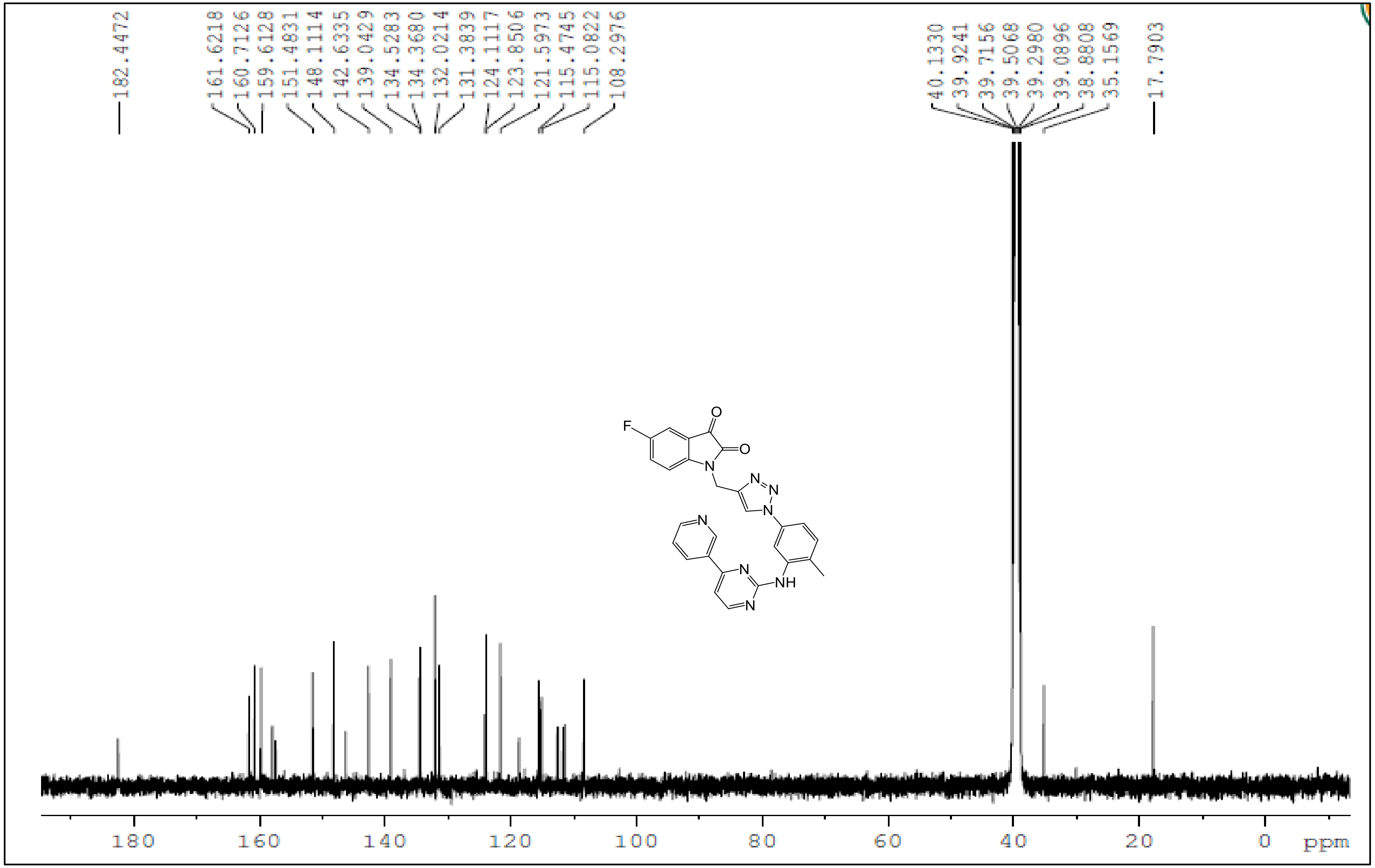

Figure S21. ^{13}C NMR of 5-fluoro-1-((1-(4-methyl-3-((4-(pyridin-3-yl)pyrimidin-2-yl)amino)phenyl)-1H-1,2,3-triazol-4-yl)methyl)indoline-2,3-dione (**2e**)

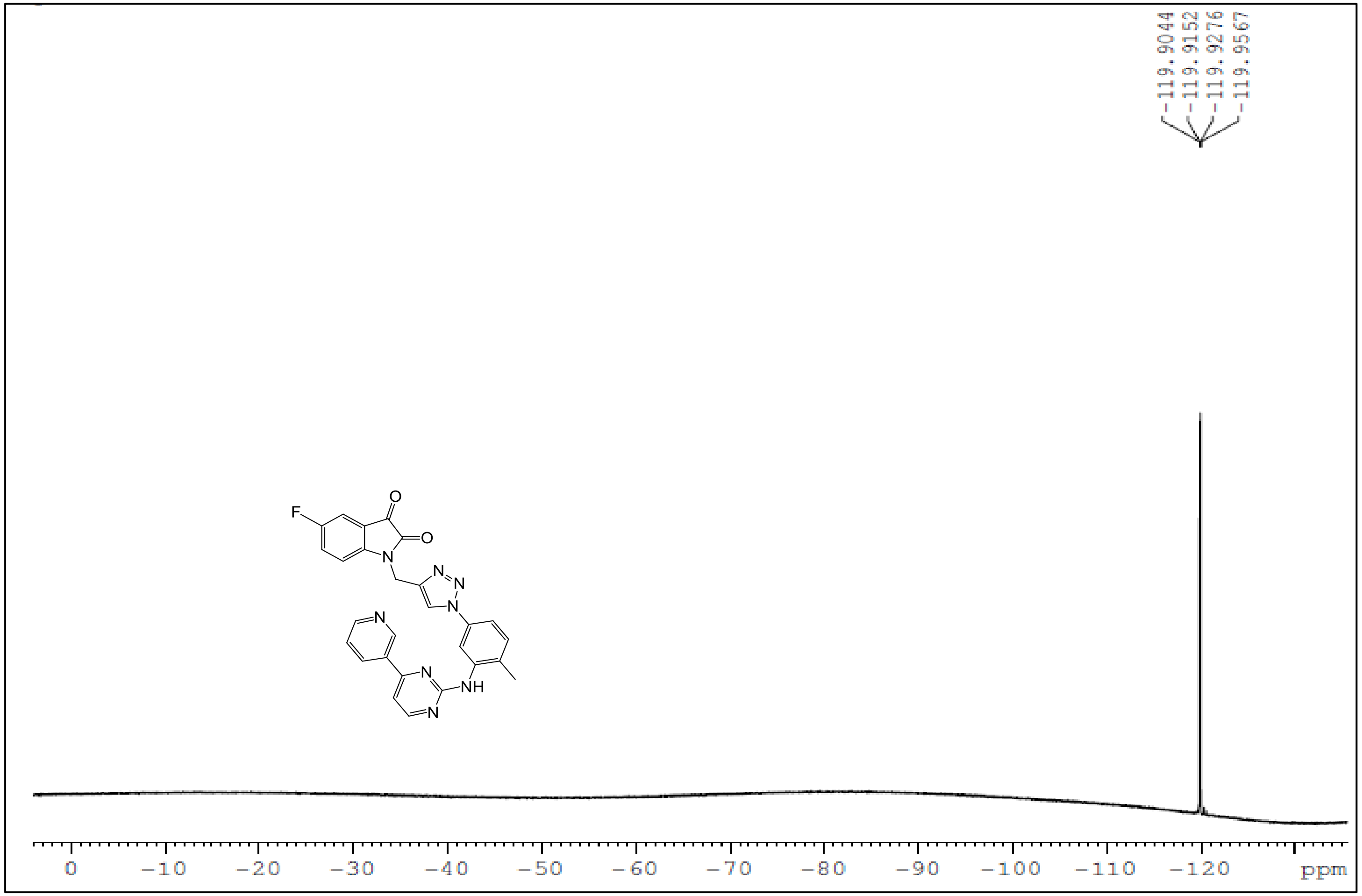

Figure S22. ^{19}F NMR of 5-fluoro-1-((1-(4-methyl-3-((4-(pyridin-3-yl)pyrimidin-2-yl)amino)phenyl)-1H-1,2,3-triazol-4-yl)methyl)indoline-2,3-dione (**2e**)

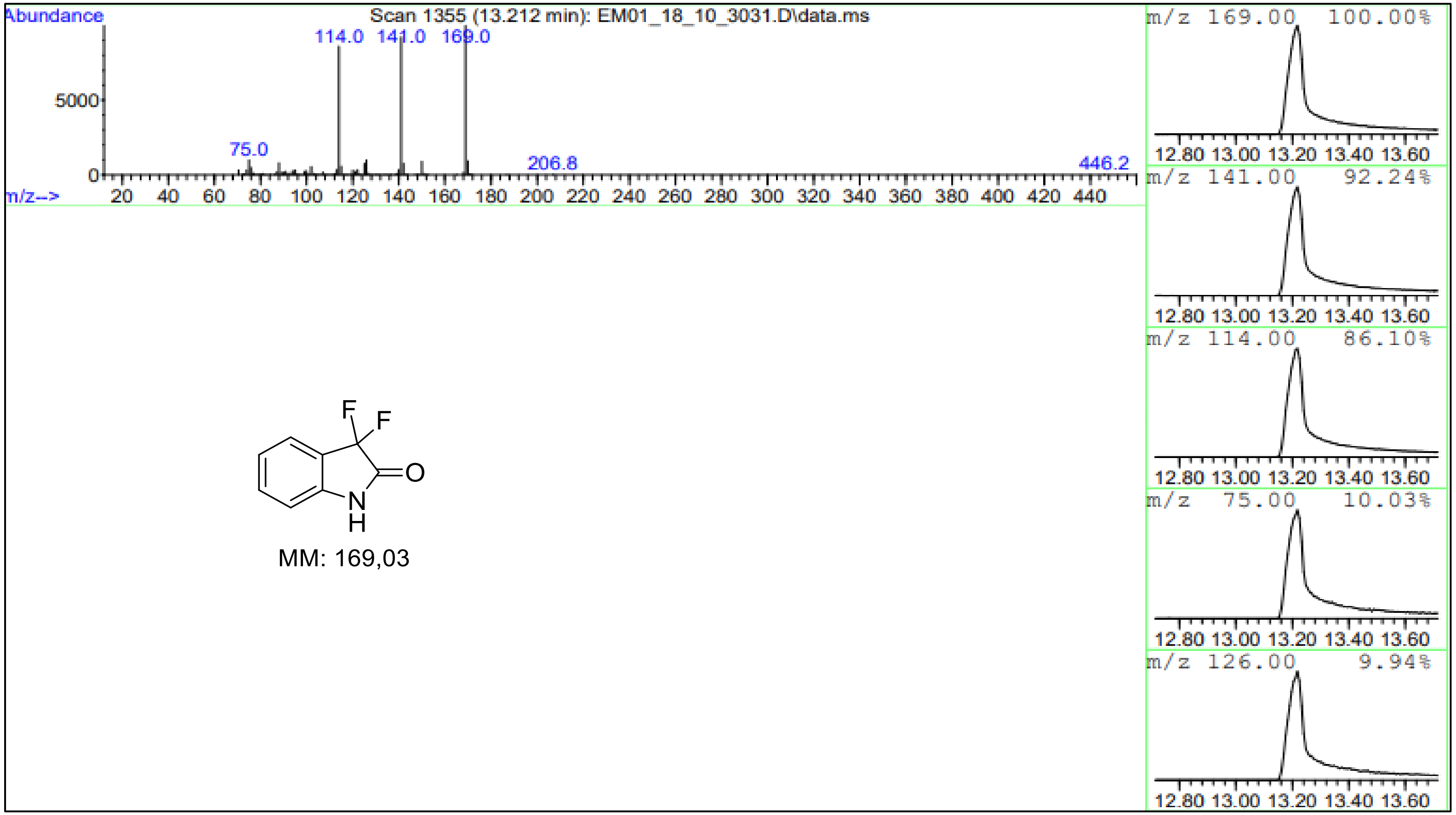

Figure S23. GC-MS of 3,3-difluoroindolin-2-one (**13a**)

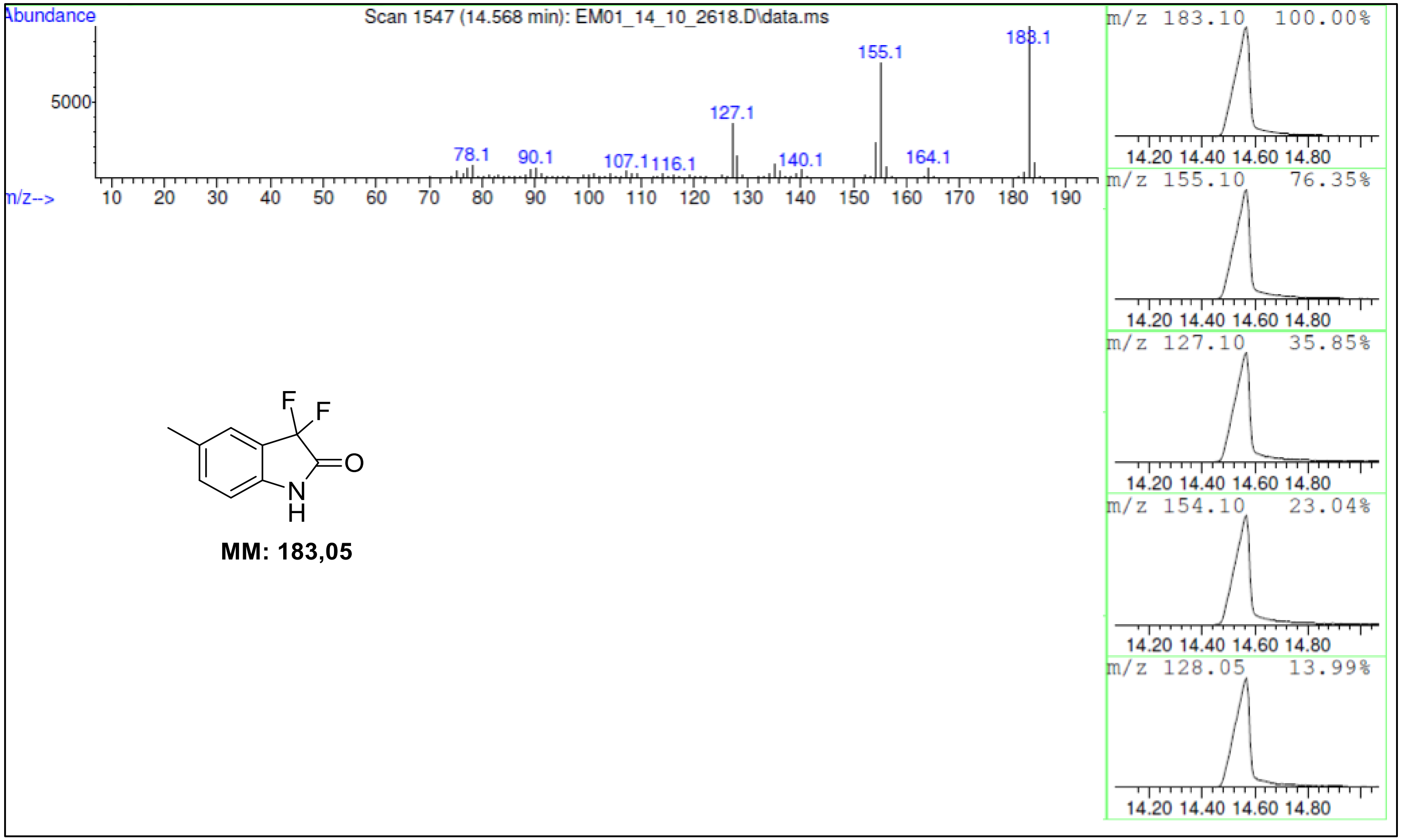

Figure S24. GC-MS of 3,3-difluoro-5-methylindolin-2-one (**13b**)

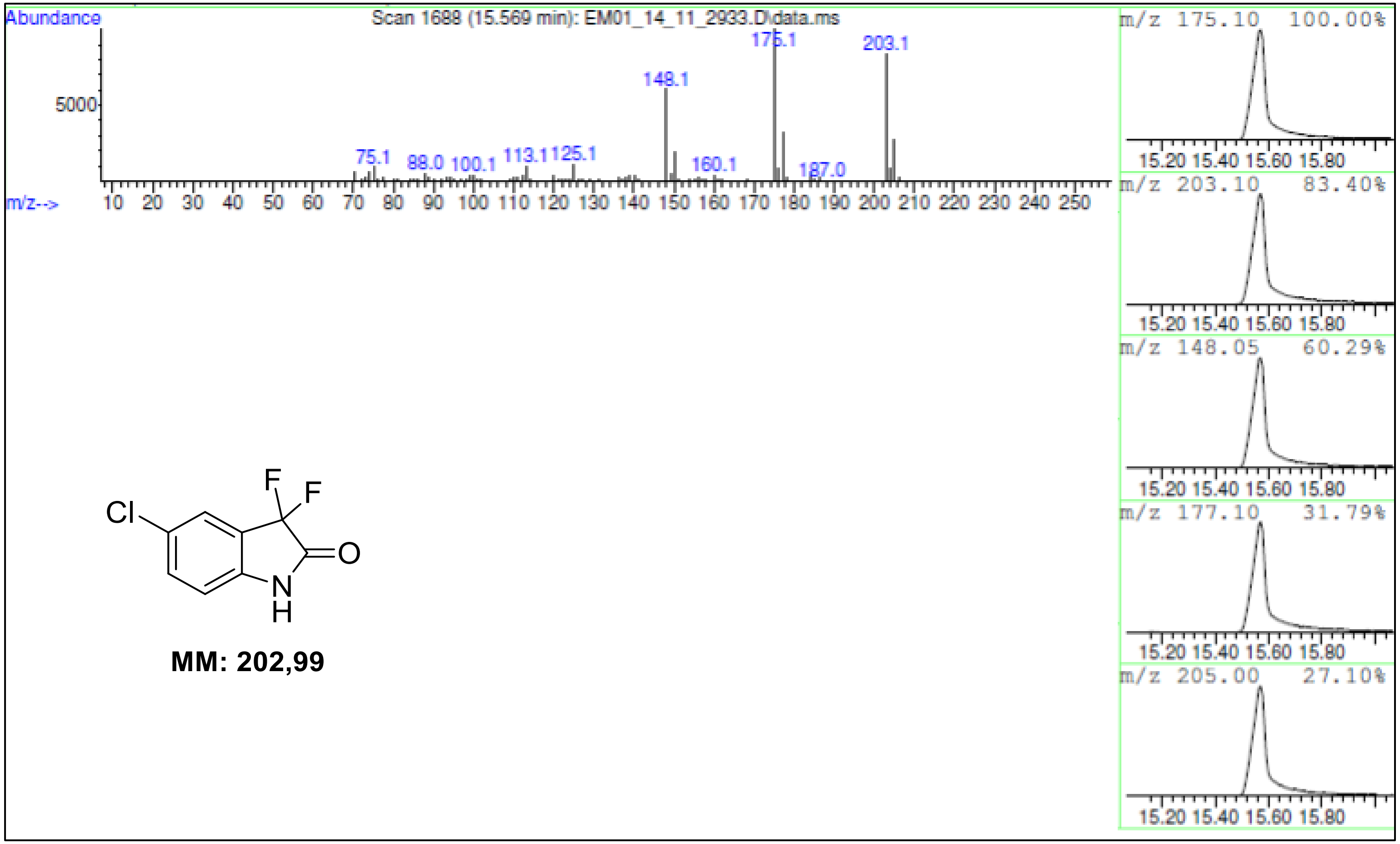

Figure S25. GC-MS of 5-chloro-3,3-difluoroindolin-2-one (**13c**)

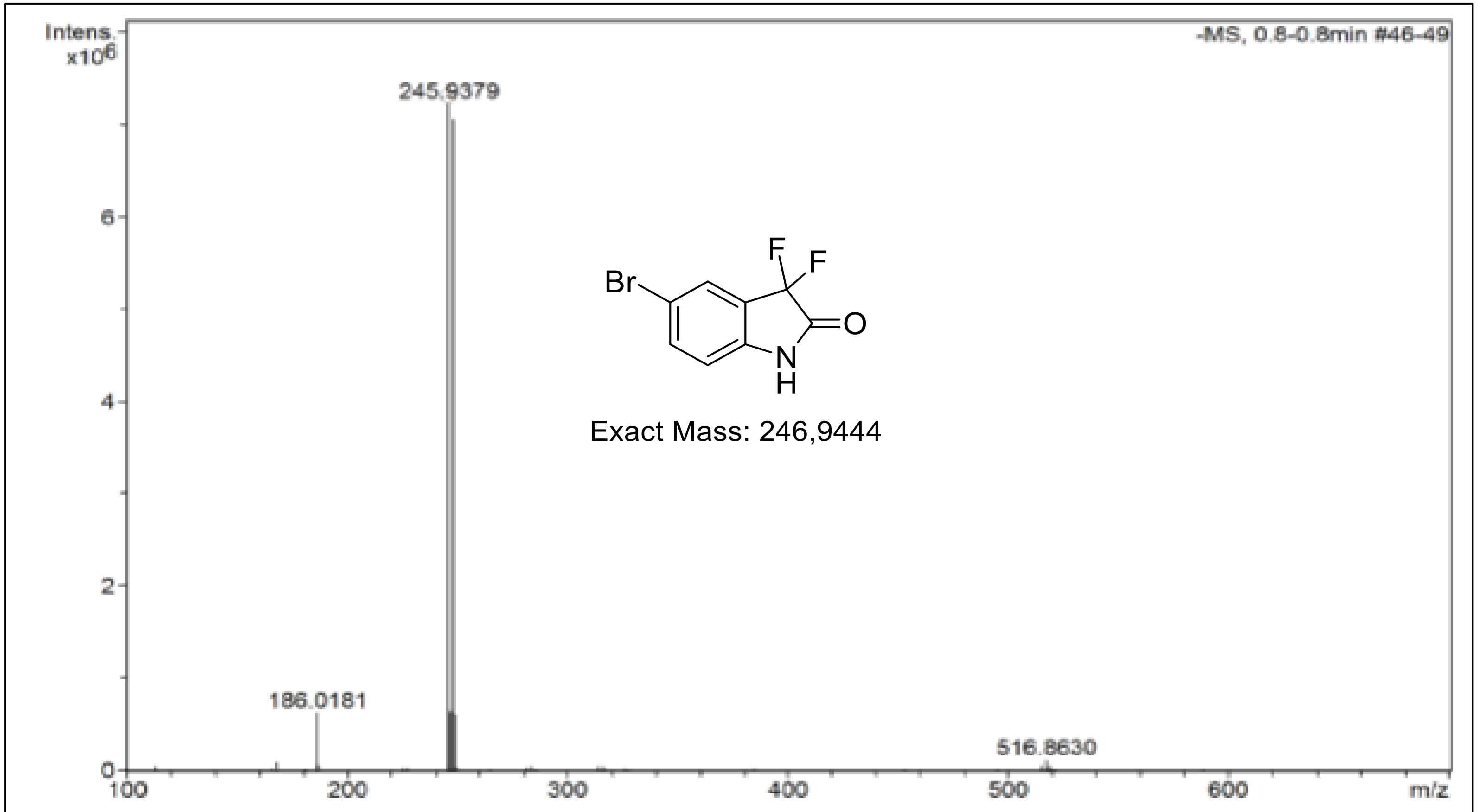

Meas. m/z	#	Ion Formula	m/z	err [ppm]	Mean err [ppm]	rdb	N-Rule	e⁻ Conf	mSigma	Std I	Std Mean m/z	Std I VarNorm	Std m/z Diff	Std Comb Dev
245.937909	1	C8H3BrF2NO	245.937156	-3.1	-3.2	6.5	ok	even	3.2	3.5	n.a.	n.a.	n.a.	n.a.

Figure S26. HRMS of 5-bromo-3,3-difluoroindolin-2-one (**13d**)

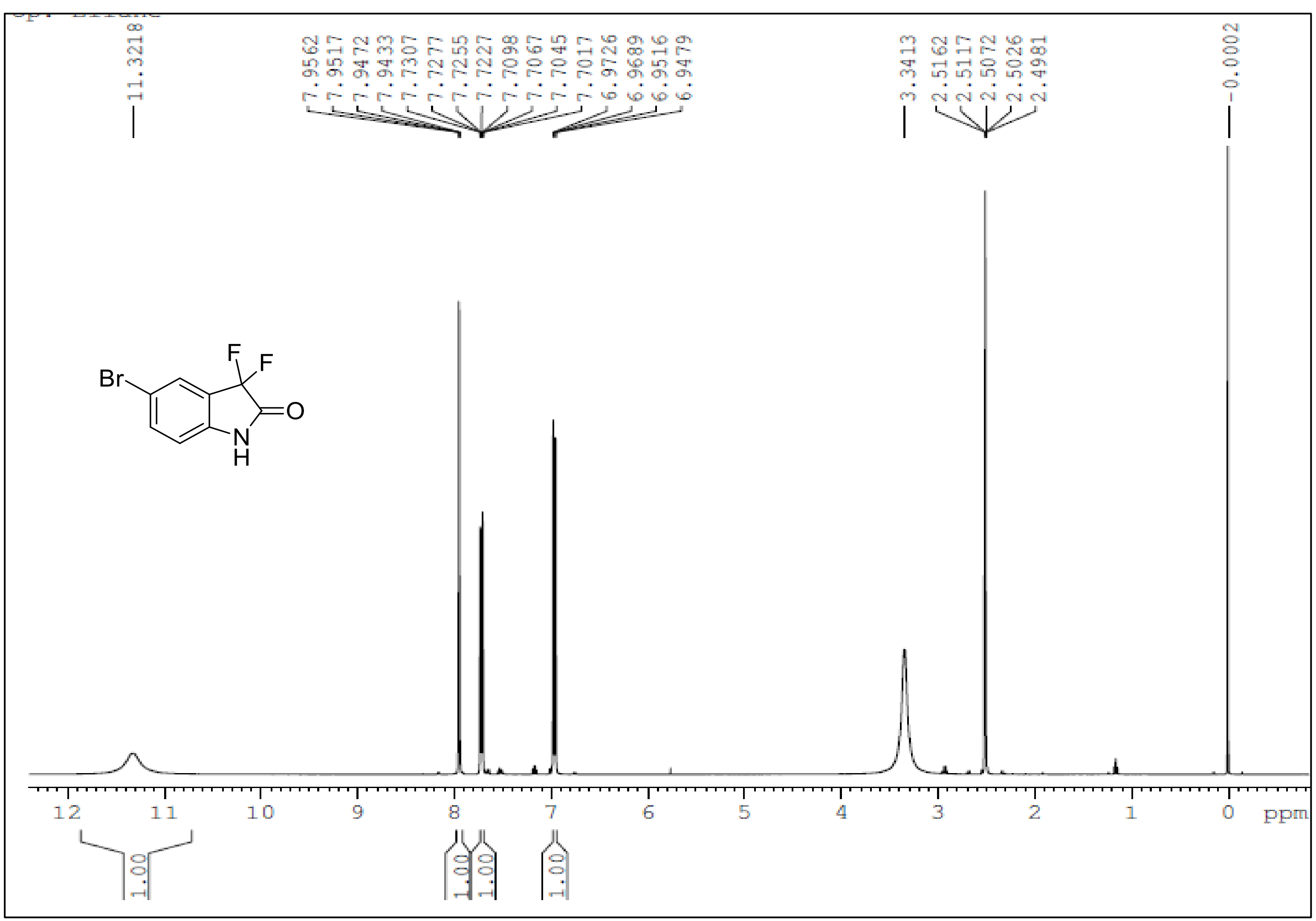

Figure S27. ^{1}H NMR of 5-bromo-3,3-difluoroindolin-2-one (**13d**)

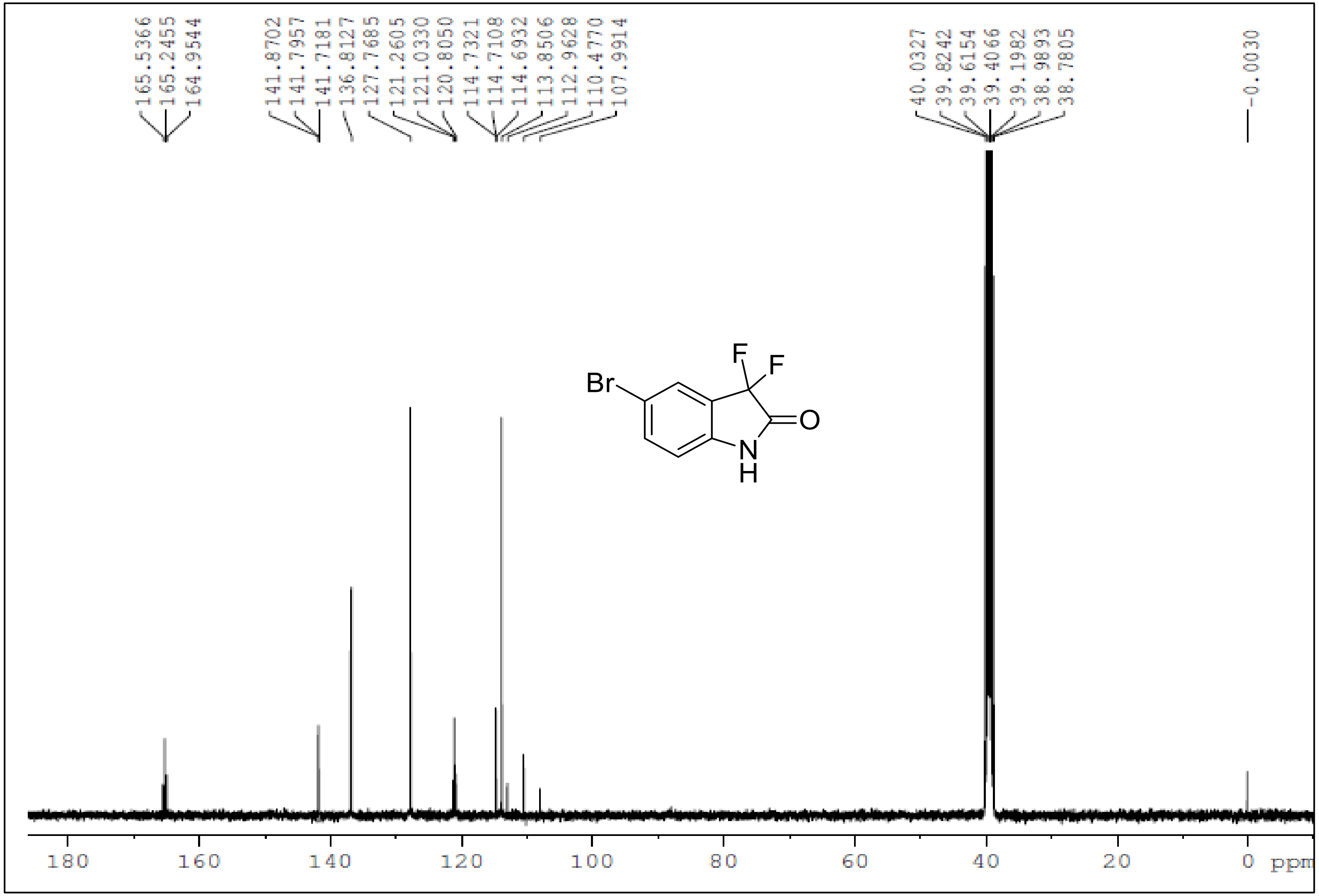

Figure S28. ^{13}C NMR of 5-bromo-3,3-difluoroindolin-2-one (**13d**)

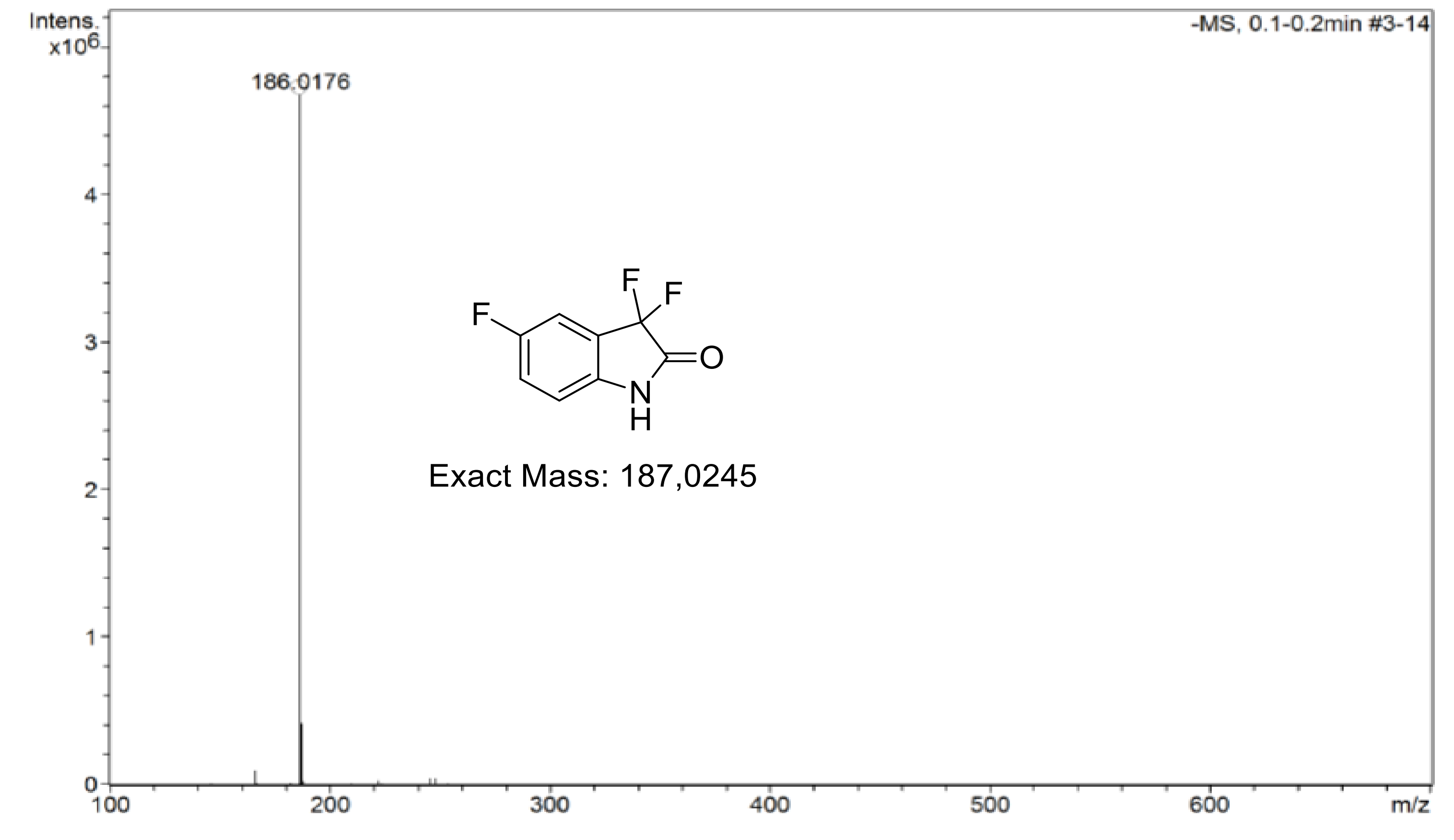

Meas. m/z	#	Ion Formula	m/z	err [ppm]	Mean err [ppm]	rdb	N-Rule	e⁻ Conf	mSigma	Std I	Std Mean m/z	Std I VarNorm	Std m/z Diff	Std Comb Dev
186.017575	1	C8H3F3NO	186.017222	-1.9	-2.0	6.5	ok	even	0.9	2.1	n.a.	n.a.	n.a.	n.a.

Figure S29. HRMS of 3,3,5-trifluoroindolin-2-one (**13e**)

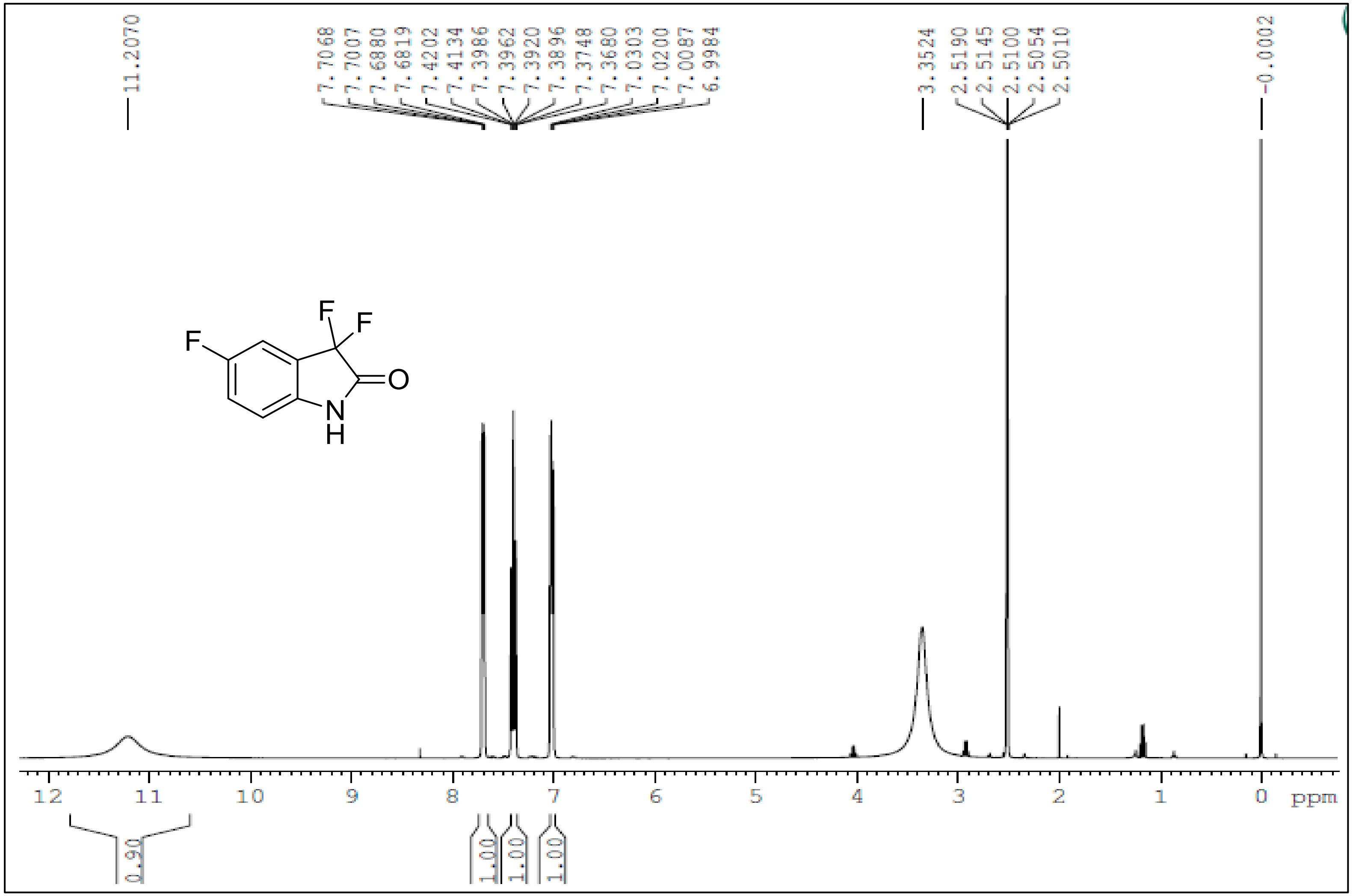

Figure S30. ^{1}H NMR of 3,3,5-trifluoroindolin-2-one (**13e**)

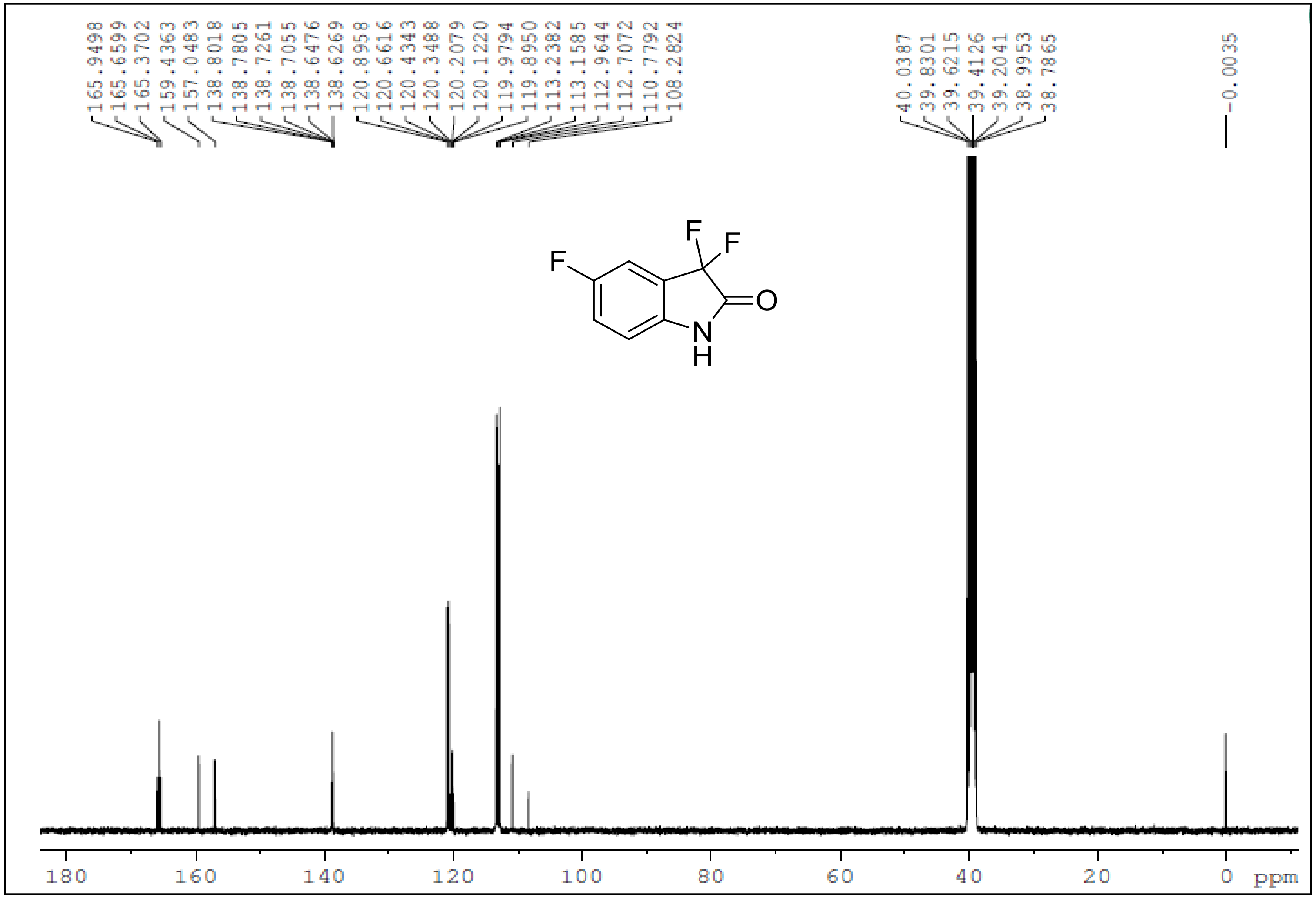

Figure S31. ^{13}C NMR of 3,3,5-trifluoroindolin-2-one (**13e**)

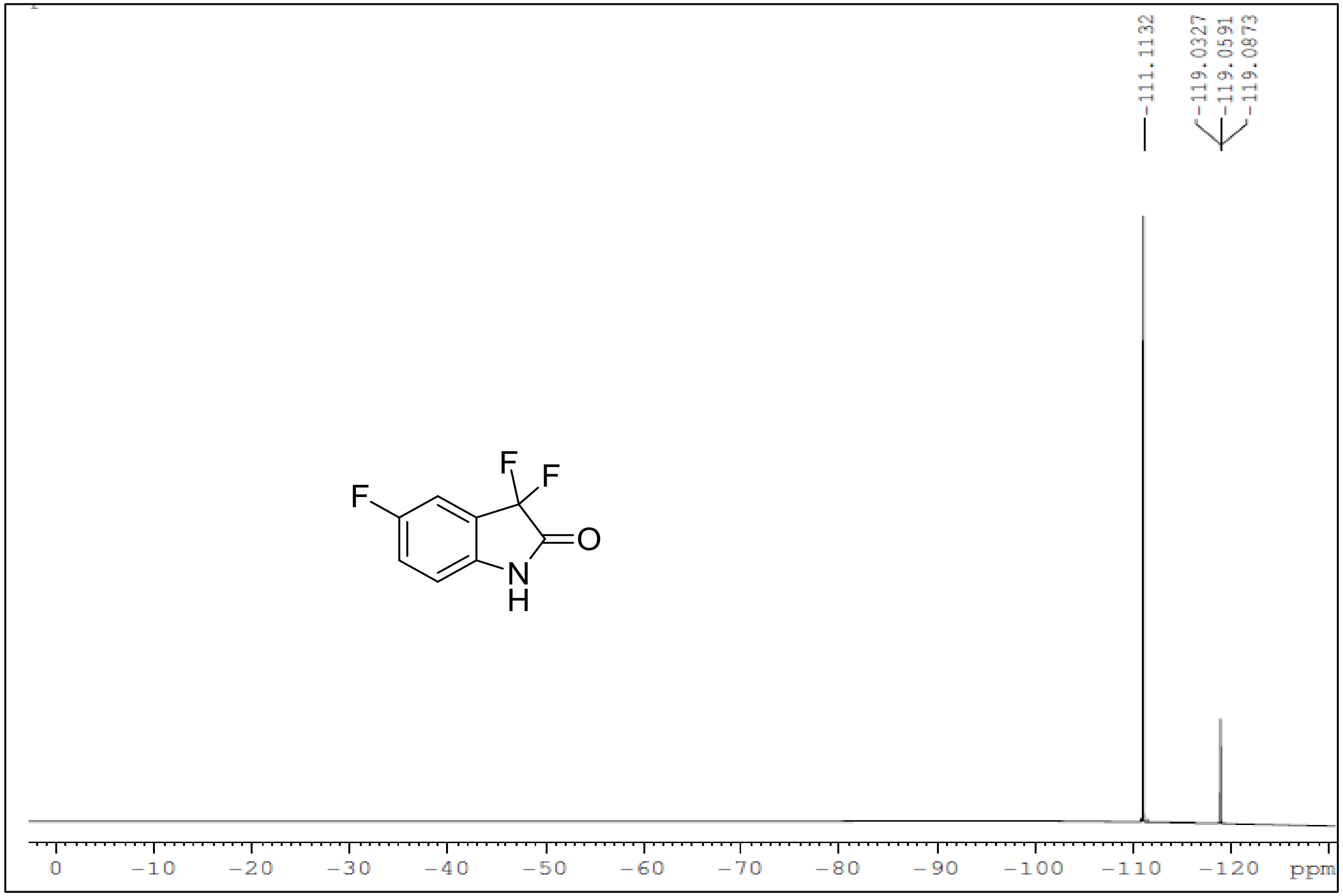

Figure S32. ^{19}F NMR of 3,3,5-trifluoroindolin-2-one (**13e**)

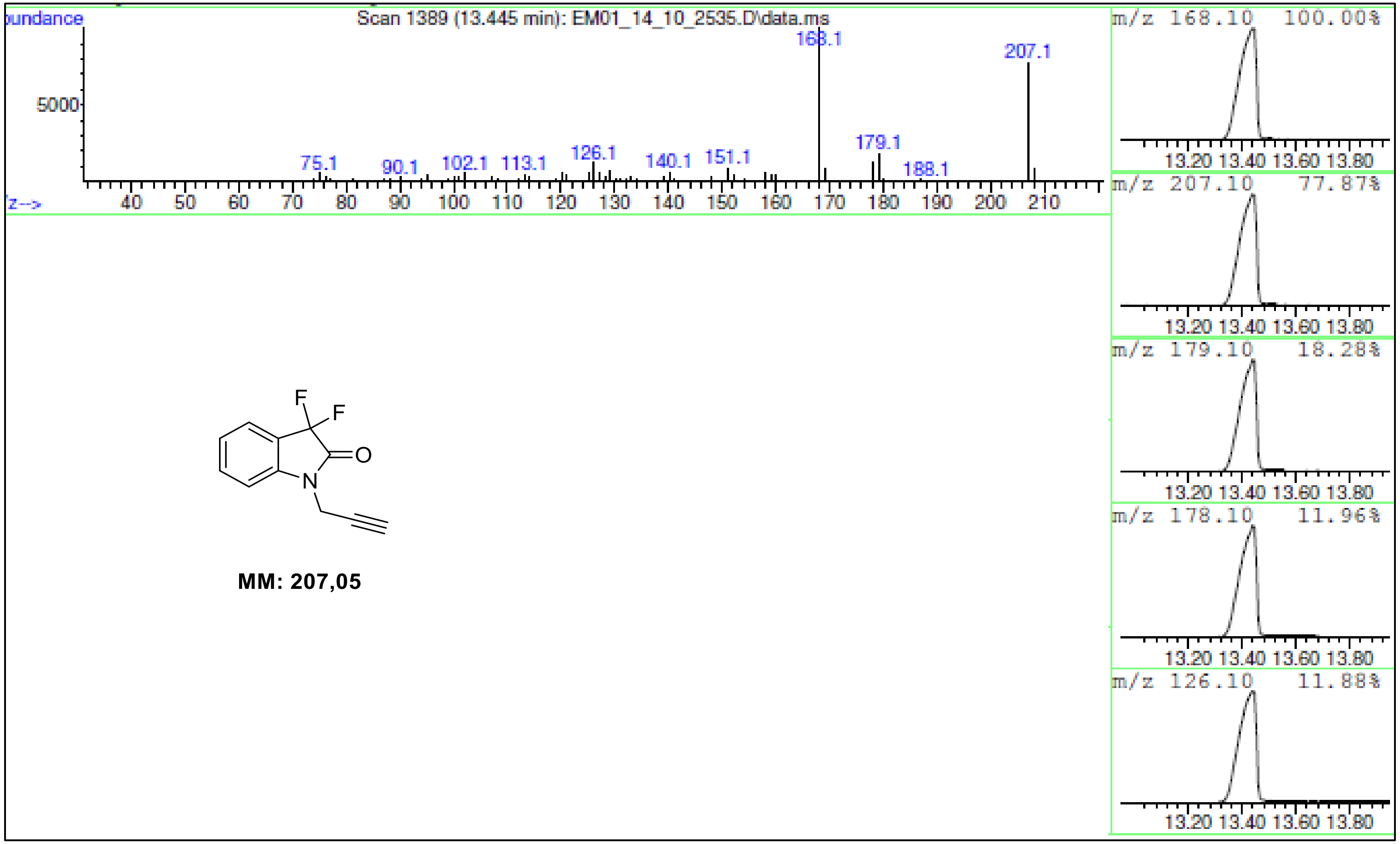

Figure S33. GC-MS of 3,3-difluoro-1-(prop-2-yn-1-yl)indolin-2-one (**14a**)

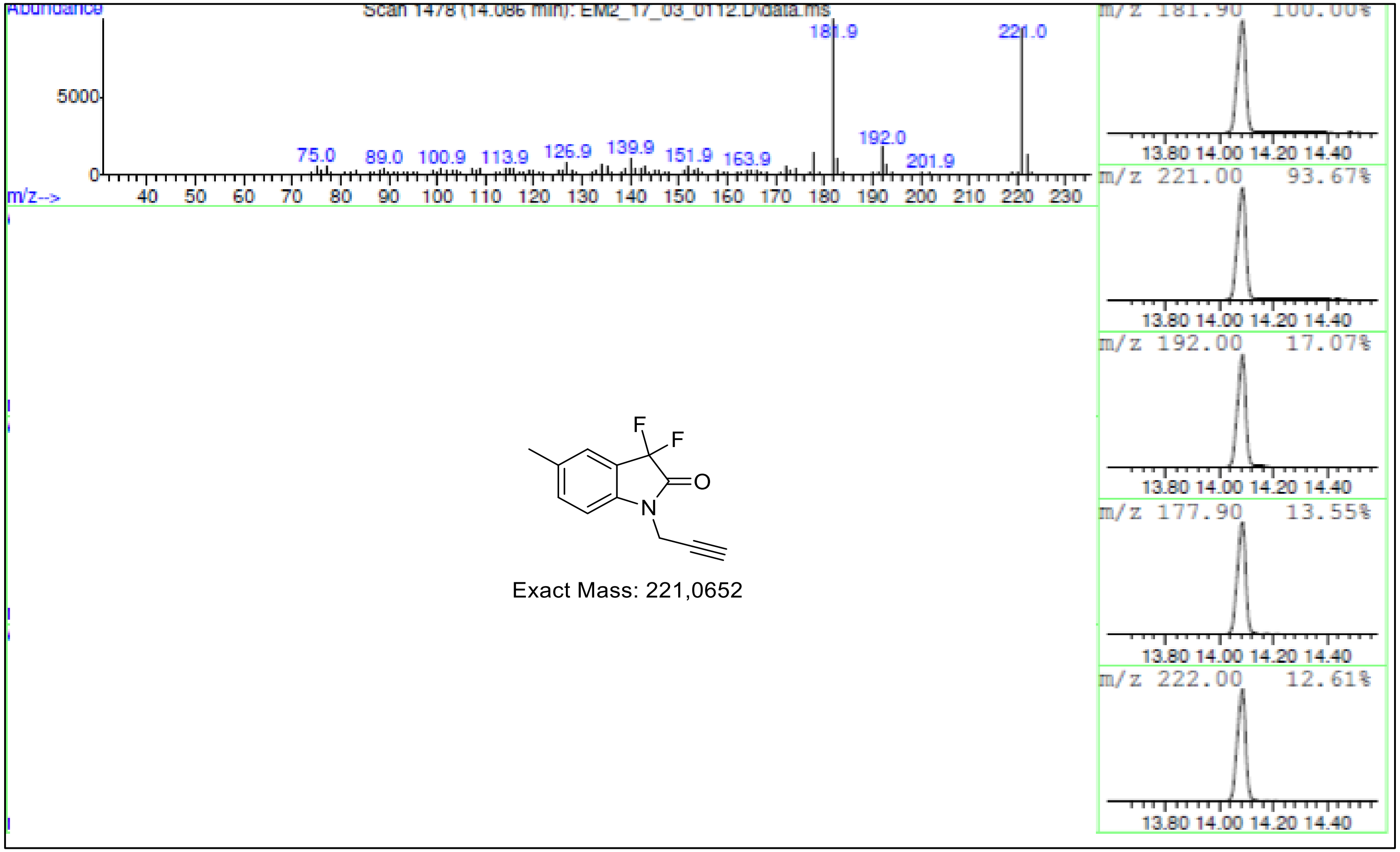

Figure S34. GC-MS of 3,3-difluoro-5-methyl-1-(prop-2-yn-1-yl)indolin-2-one (**14b**)

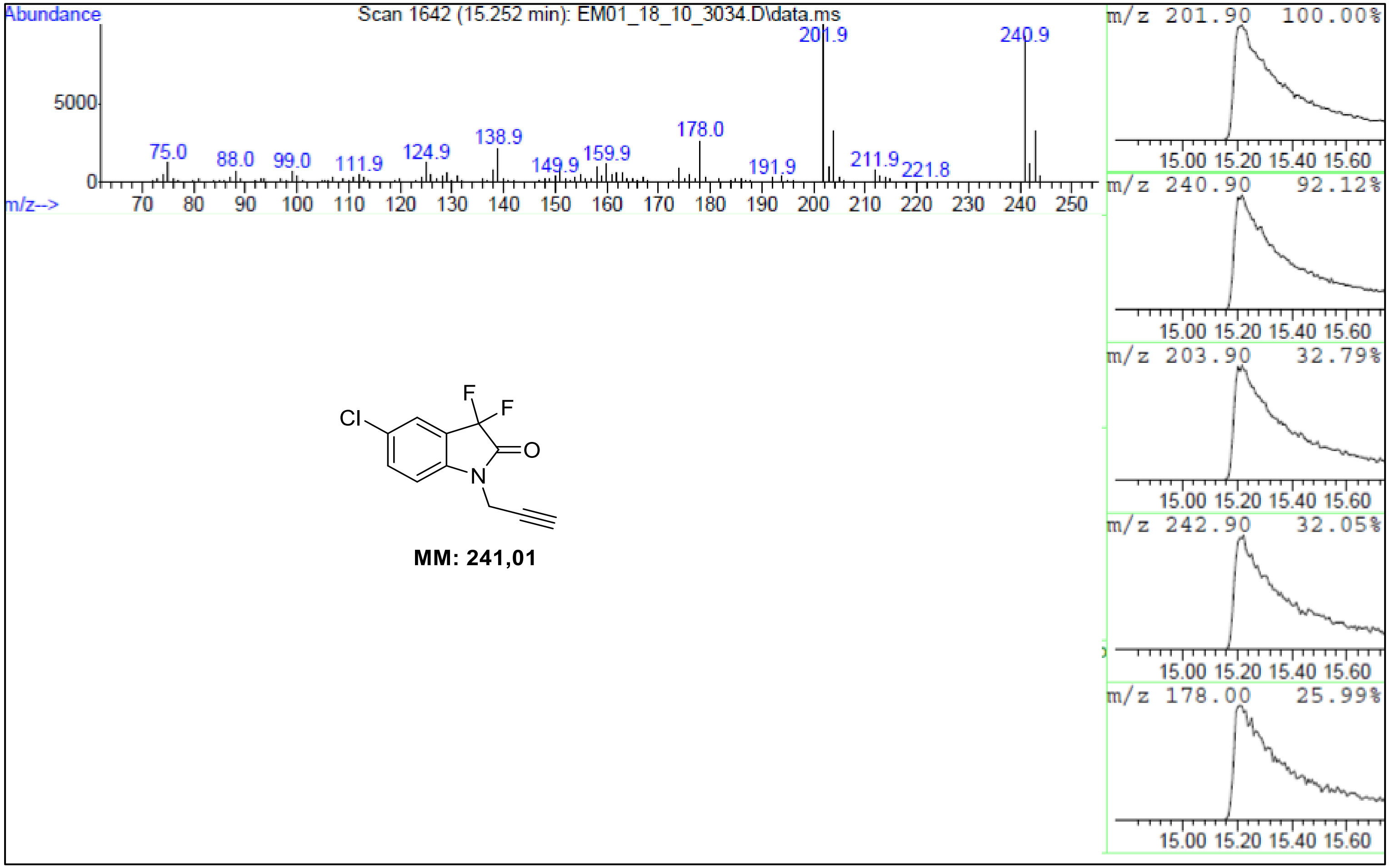

Figure S35. GC-MS of 5-chloro-3,3-difluoro-1-(prop-2-yn-1-yl)indolin-2-one (**14c**)

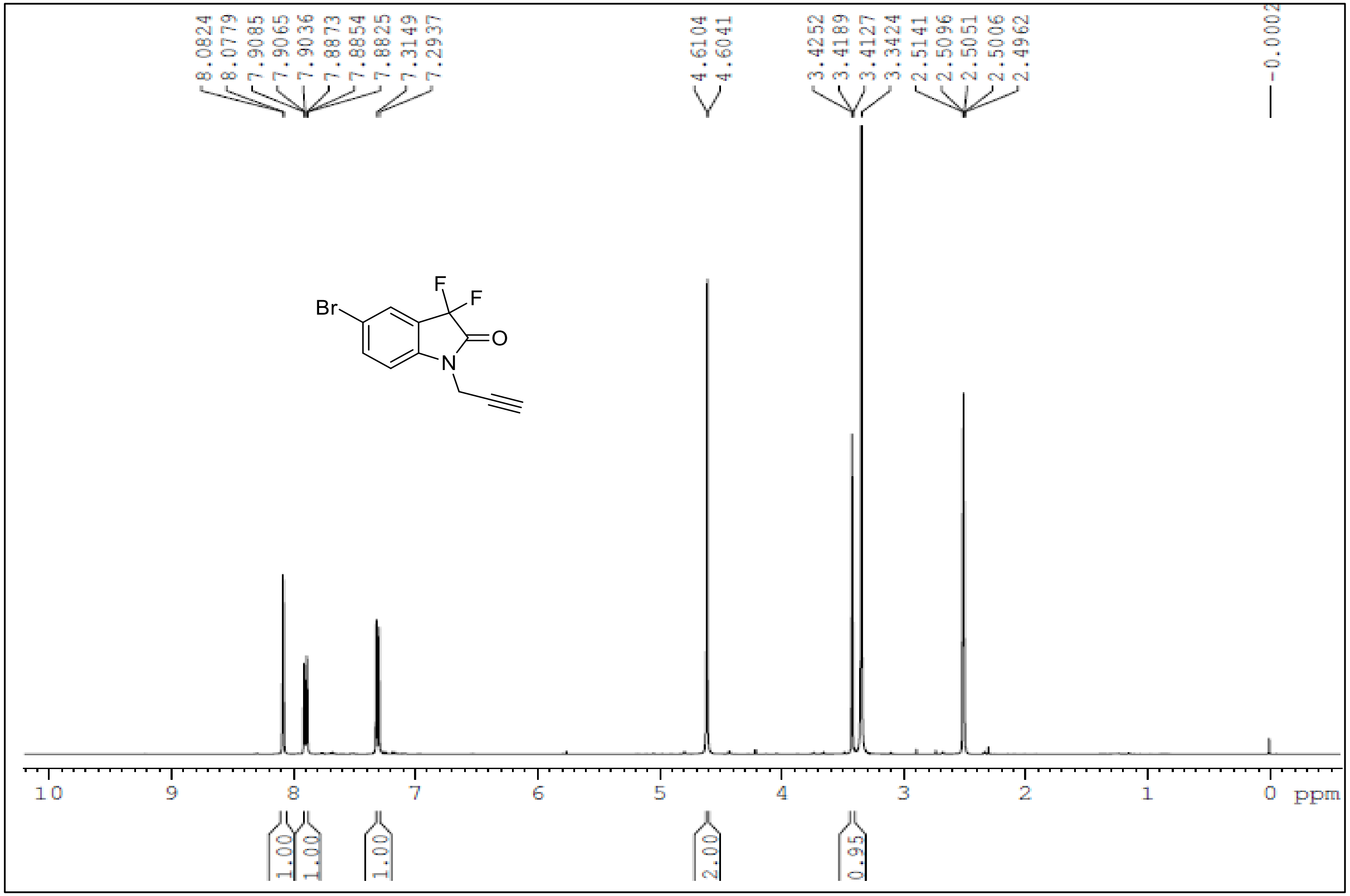

Figure S36. ^{1}H NMR of 5-bromo-3,3-difluoro-1-(prop-2-yn-1-yl)indolin-2-one (**14d**)

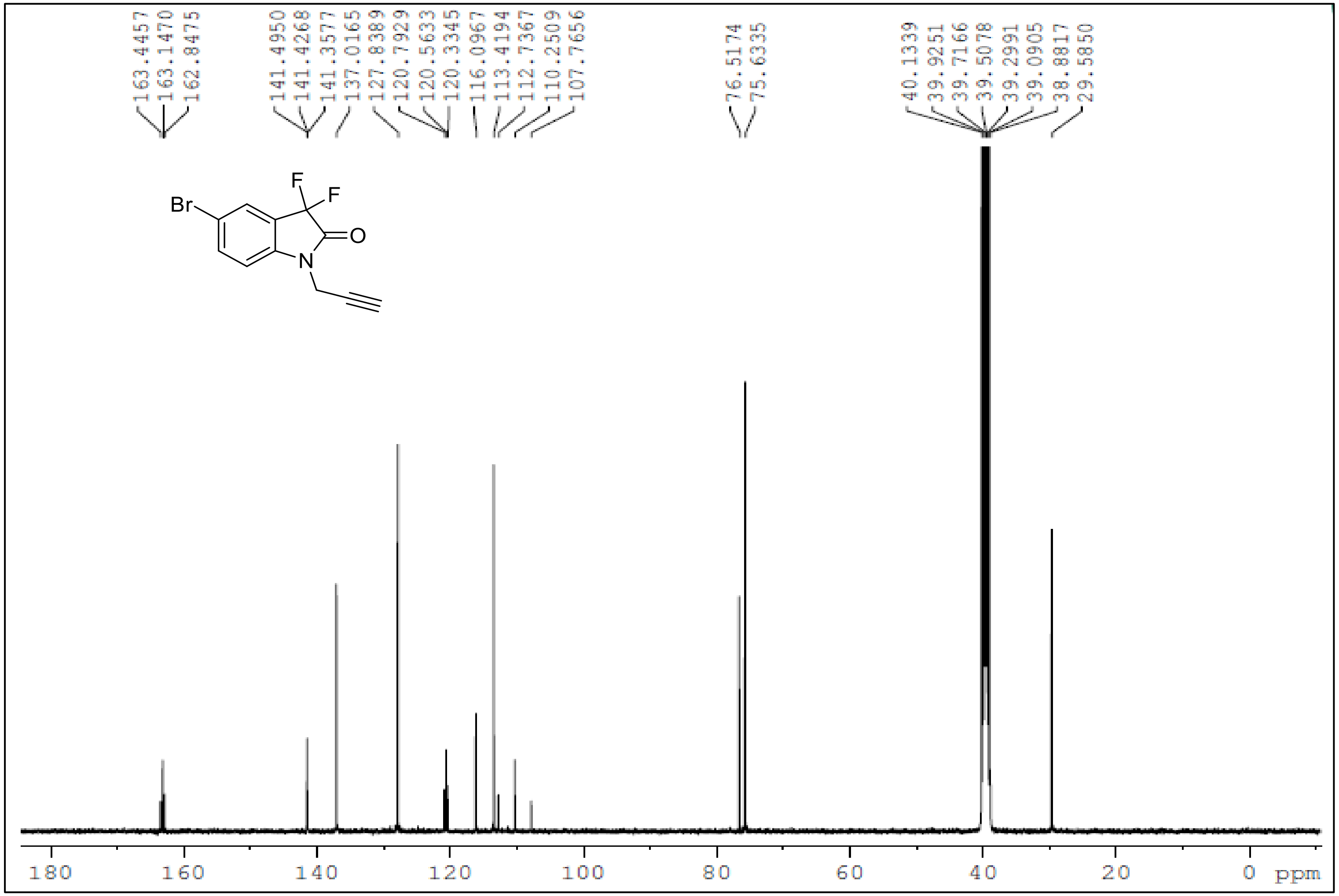

Figure S37. ^{13}C NMR of 5-bromo-3,3-difluoro-1-(prop-2-yn-1-yl)indolin-2-one (**14d**)

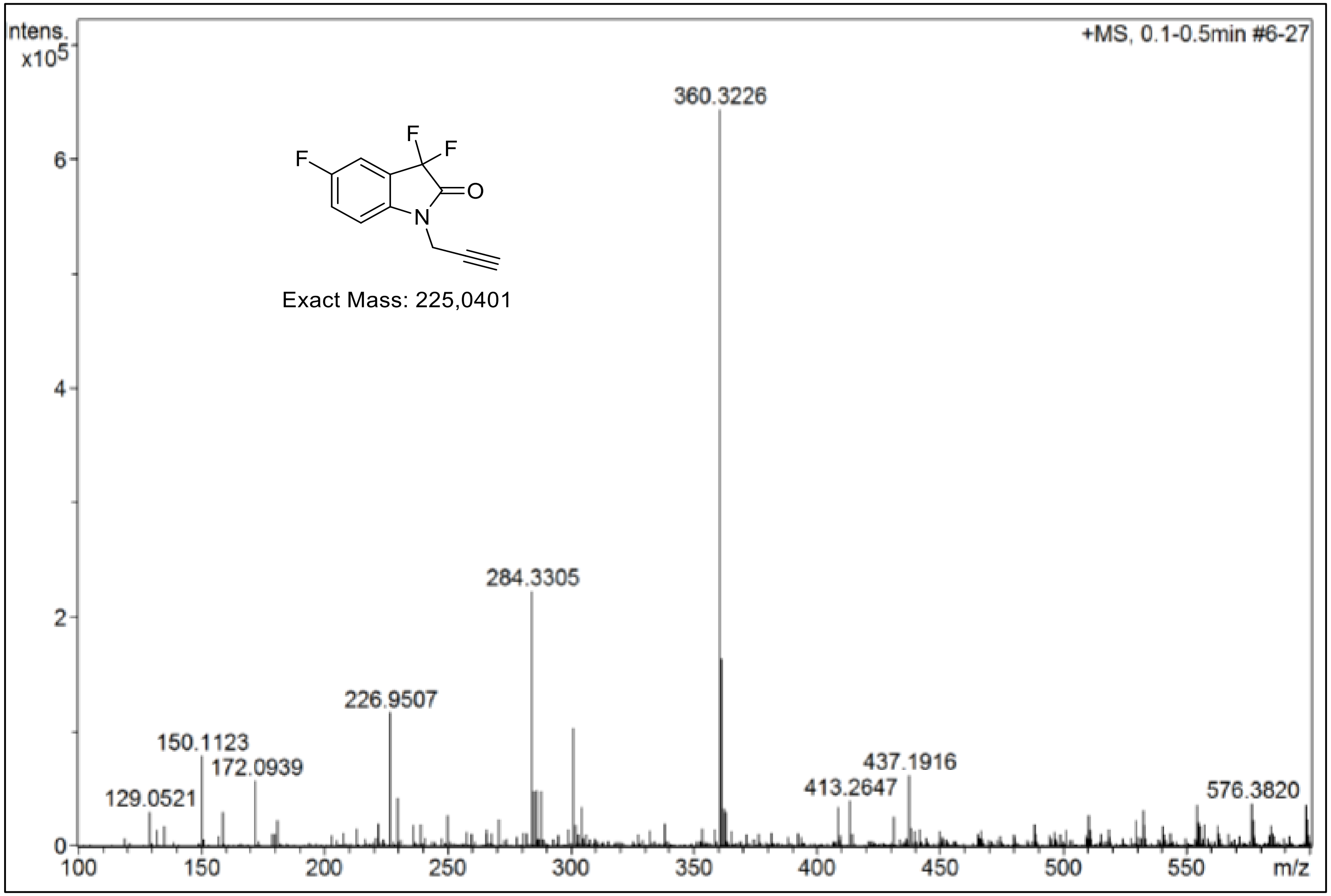

Figure S38. HRMS of 3,3,5-trifluoro-1-(prop-2-yn-1-yl)indolin-2-one (**14e**)

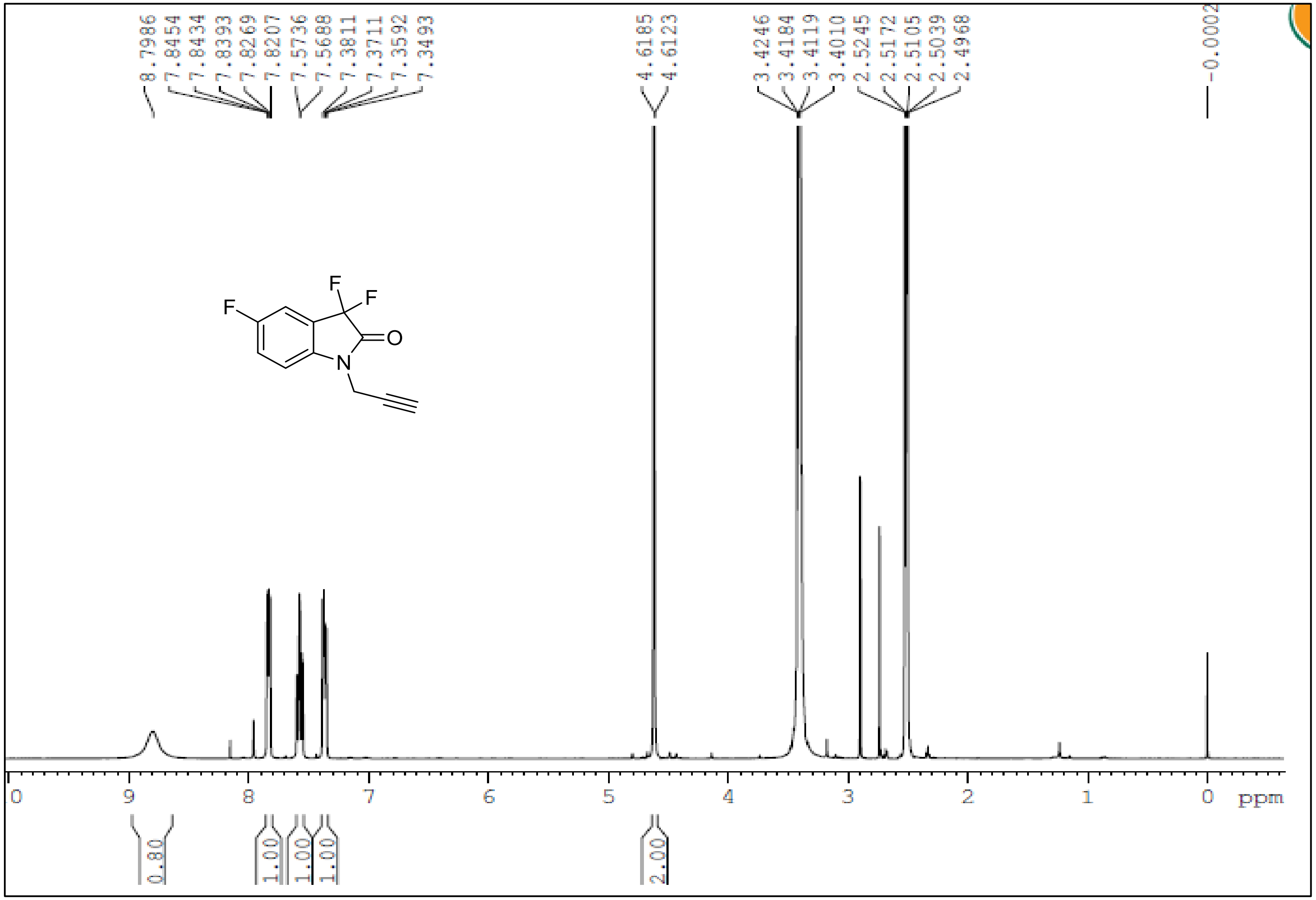

Figure S39. ^{1}H NMR of 3,3,5-trifluoro-1-(prop-2-yn-1-yl)indolin-2-one (**14e**)

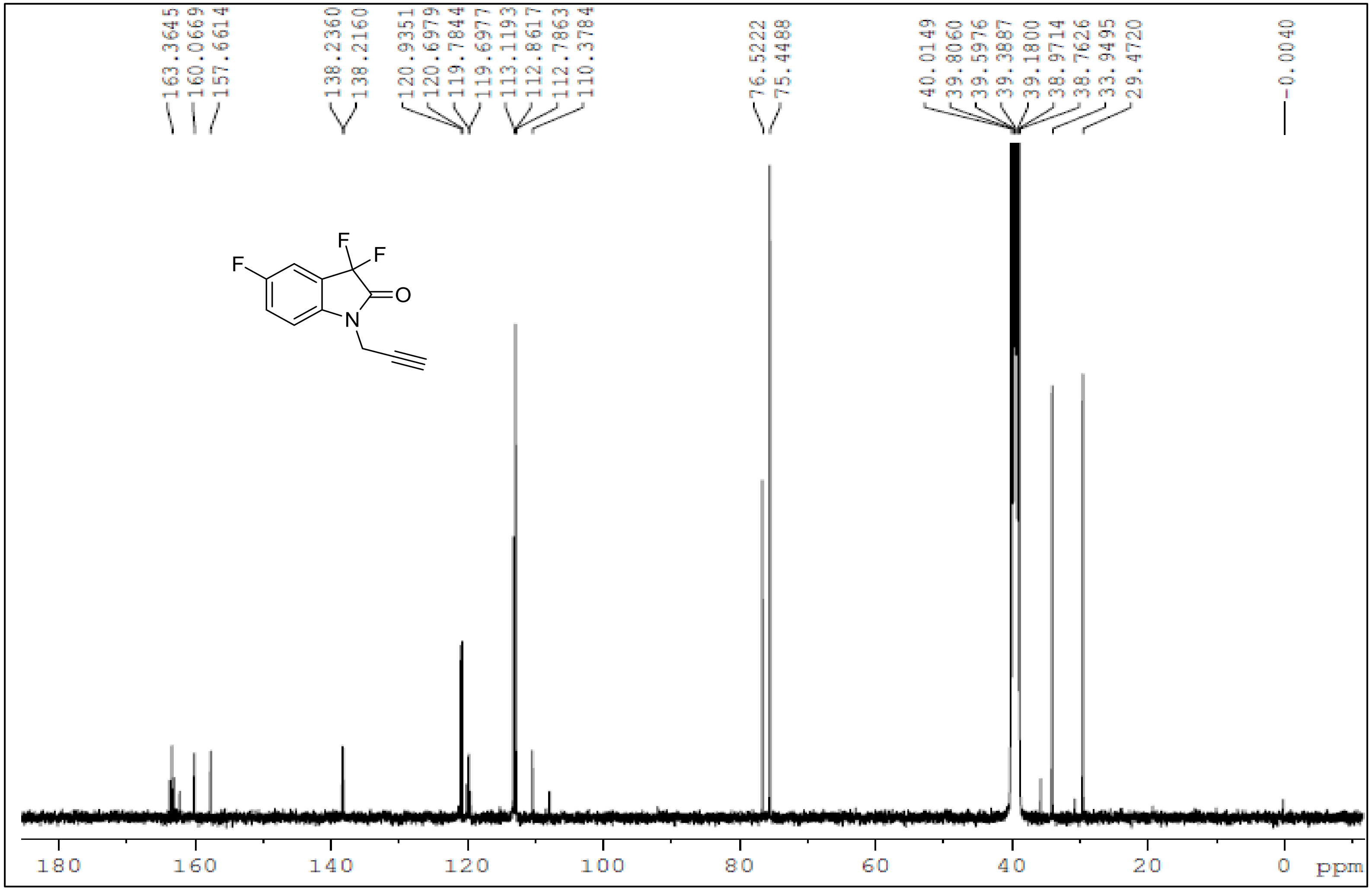

Figure S40. ^{13}C NMR of 3,3,5-trifluoro-1-(prop-2-yn-1-yl)indolin-2-one (**14e**)

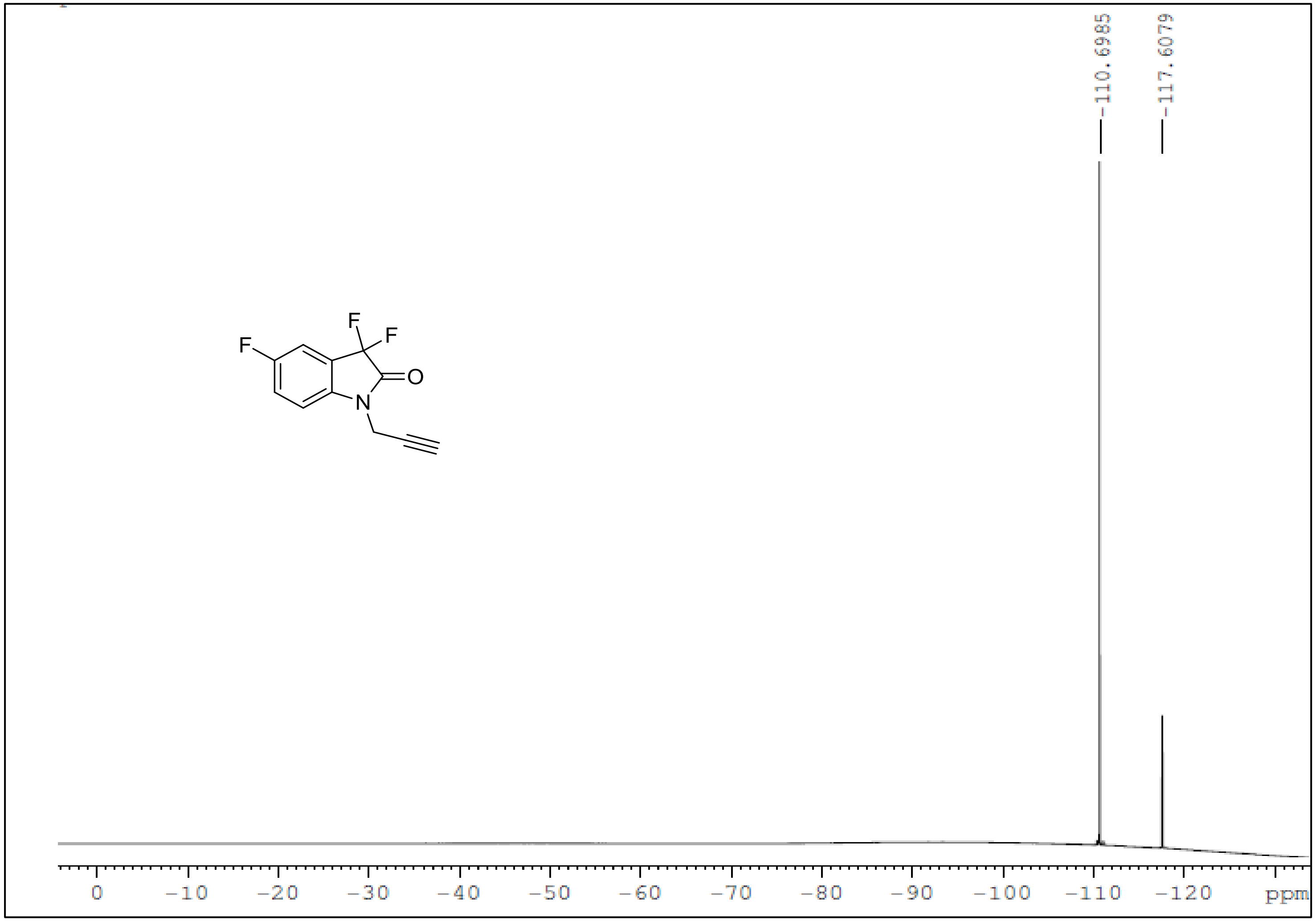

Figure S41. ^{19}F NMR of 3,3,5-trifluoro-1-(prop-2-yn-1-yl)indolin-2-one (**14e**)

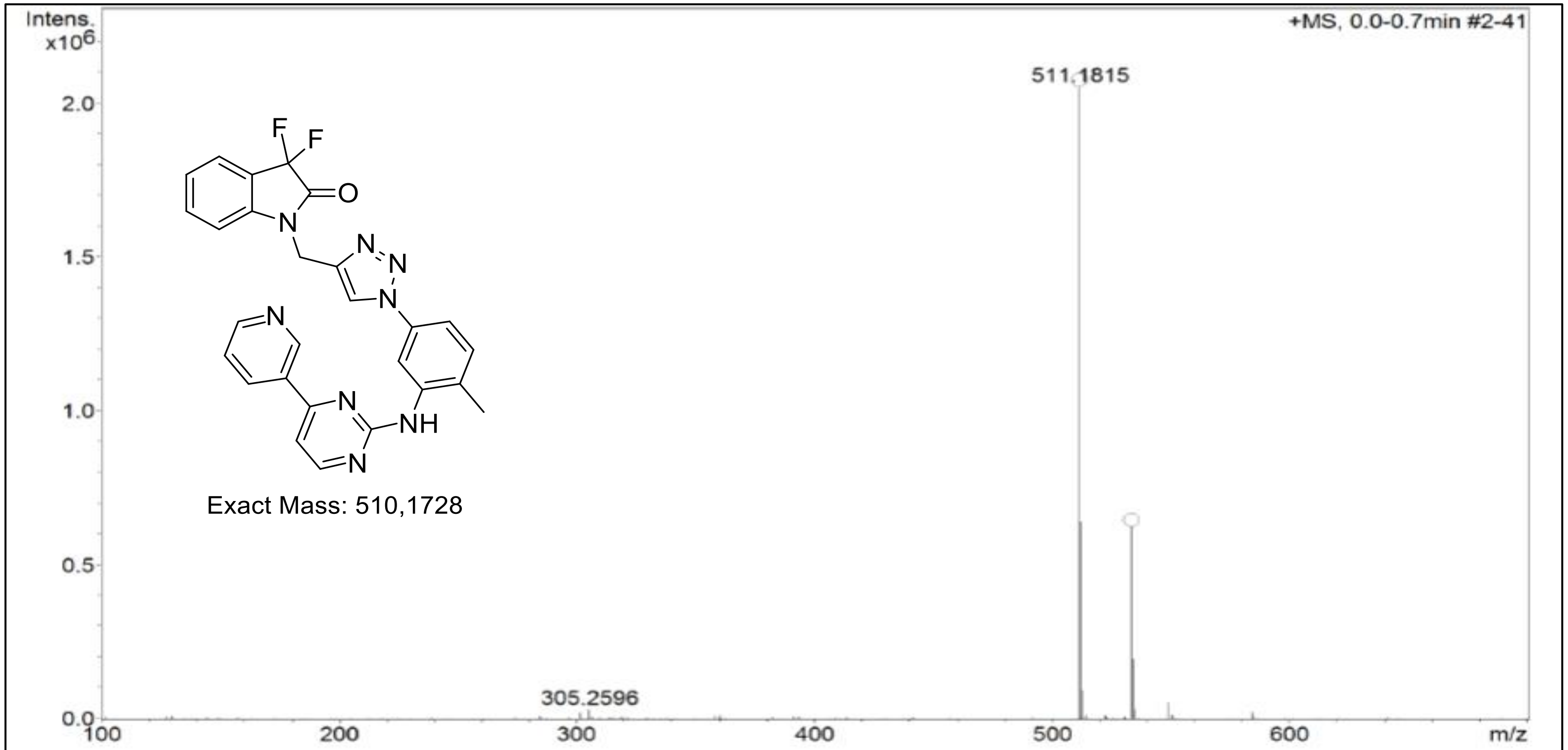

Meas. m/z	#	Ion Formula	m/z	err [ppm]	Mean err [ppm]	rdb	N-Rule	e⁻ Conf	mSigma	Std I	Std Mean m/z	Std I VarNorm	Std m/z Diff	Std Comb Dev
511.181494	1	C27H21F2N8O	511.180090	-2.7	-2.4	20.5	ok	even	7.2	11.3	n.a.	n.a.	n.a.	n.a.
	2	C26H25F2N4O5	511.178753	-5.4	-4.3	15.5	ok	even	7.6	10.9	n.a.	n.a.	n.a.	n.a.
	3	C31H25F2N2O3	511.182776	2.5	4.0	19.5	ok	even	19.0	33.8	n.a.	n.a.	n.a.	n.a.
533.163359	1	C27H20F2N8NaO	533.162034	-2.5	-2.1	20.5	ok	even	6.4	9.7	n.a.	n.a.	n.a.	n.a.
	2	C26H24F2N4NaO5	533.160697	-5.0	-3.7	15.5	ok	even	7.2	10.3	n.a.	n.a.	n.a.	n.a.
	3	C31H24F2N2NaO3	533.164720	2.6	4.3	19.5	ok	even	18.3	32.1	n.a.	n.a.	n.a.	n.a.

Figure S42. HRMS of 3,3-difluoro-1-((1-(4-methyl-3-((4-(pyridin-3-yl)pyrimidin-2-yl)amino)phenyl)-1H-1,2,3-triazol-4-yl)methyl)indolin-2-one (**3a**)

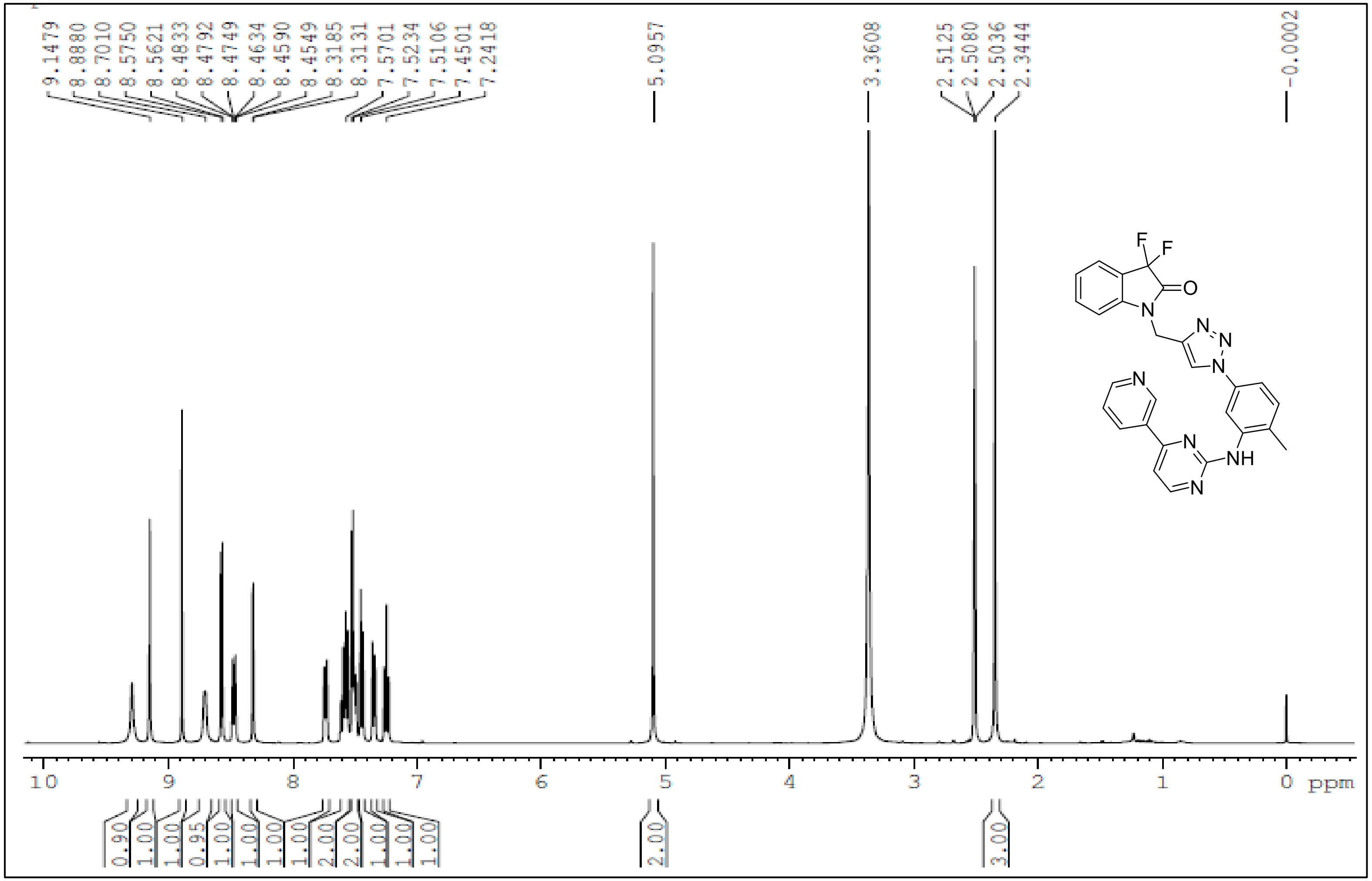

Figure S43. ¹H NMR of 3,3-difluoro-1-((1-(4-methyl-3-((4-(pyridin-3-yl)pyrimidin-2-yl)amino)phenyl)-1H-1,2,3-triazol-4-yl)methyl)indolin-2-one (**3a**)

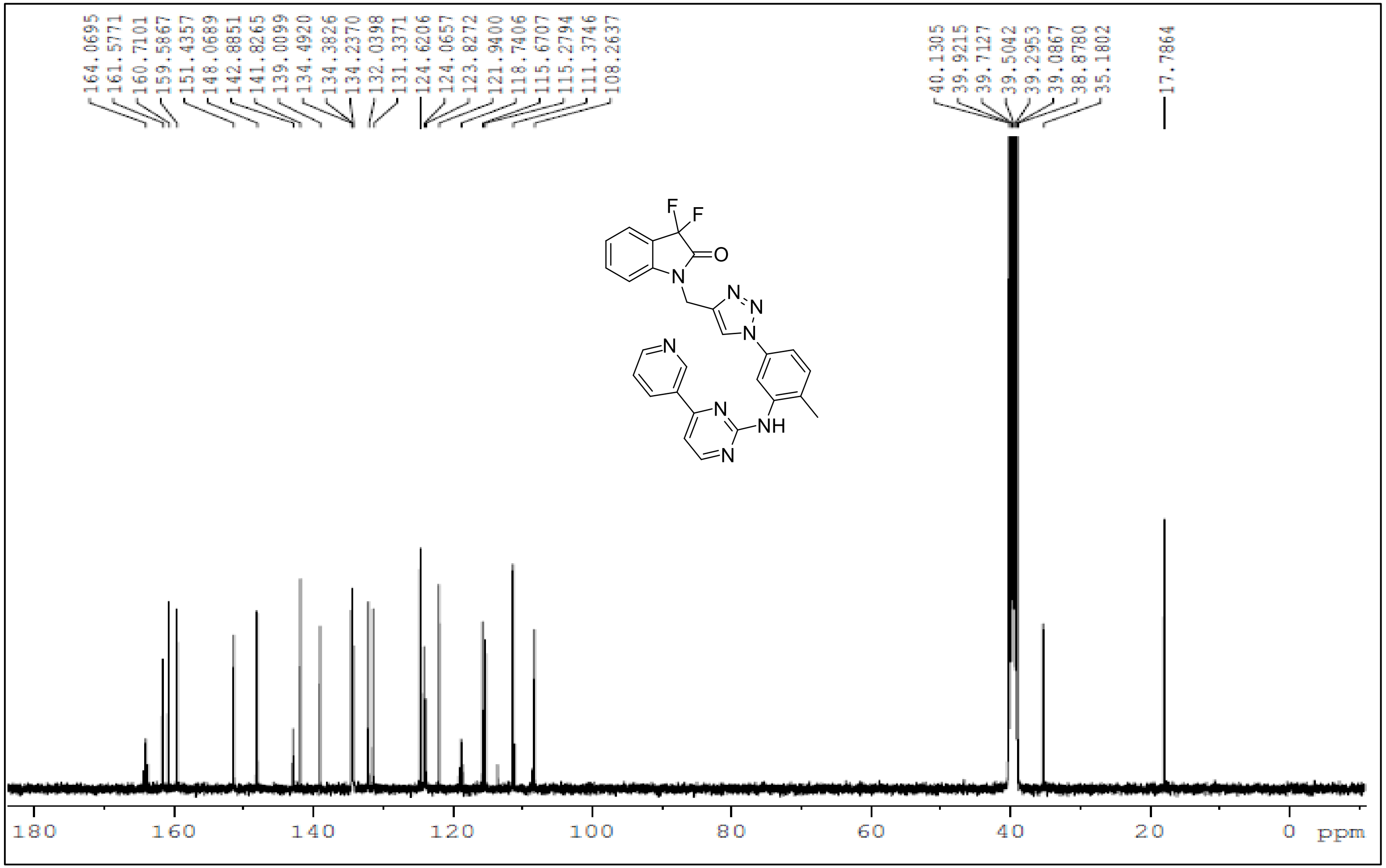

Figure S44. ^{13}C NMR of 3,3-difluoro-1-((1-(4-methyl-3-((4-(pyridin-3-yl)pyrimidin-2-yl)amino)phenyl)-1H-1,2,3-triazol-4-yl)methyl)indolin-2-one (**3a**)

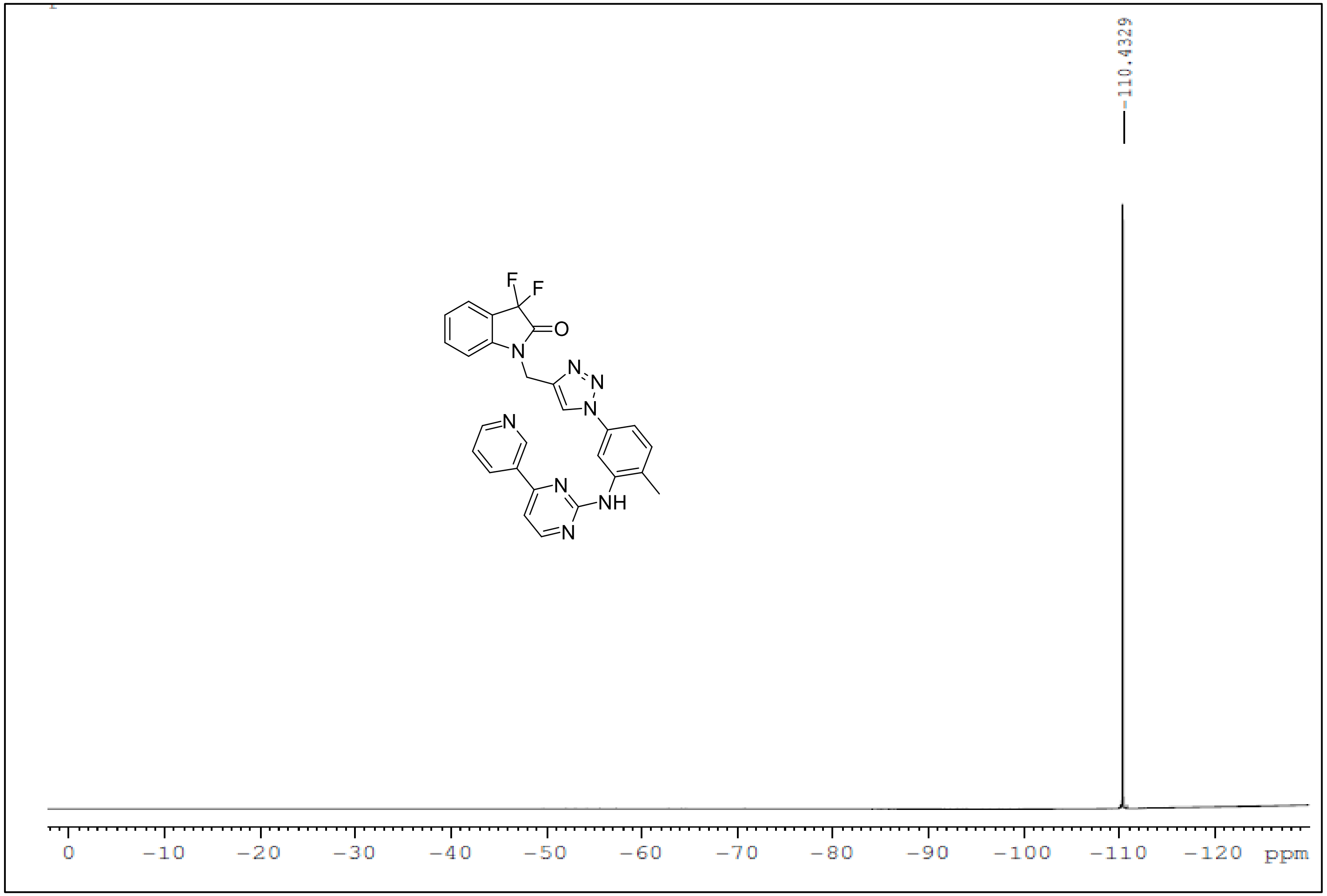

Figure S45. ^{19}F NMR of 3,3-difluoro-1-((1-(4-methyl-3-((4-(pyridin-3-yl)pyrimidin-2-yl)amino)phenyl)-1H-1,2,3-triazol-4-yl)methyl)indolin-2-one (**3a**)

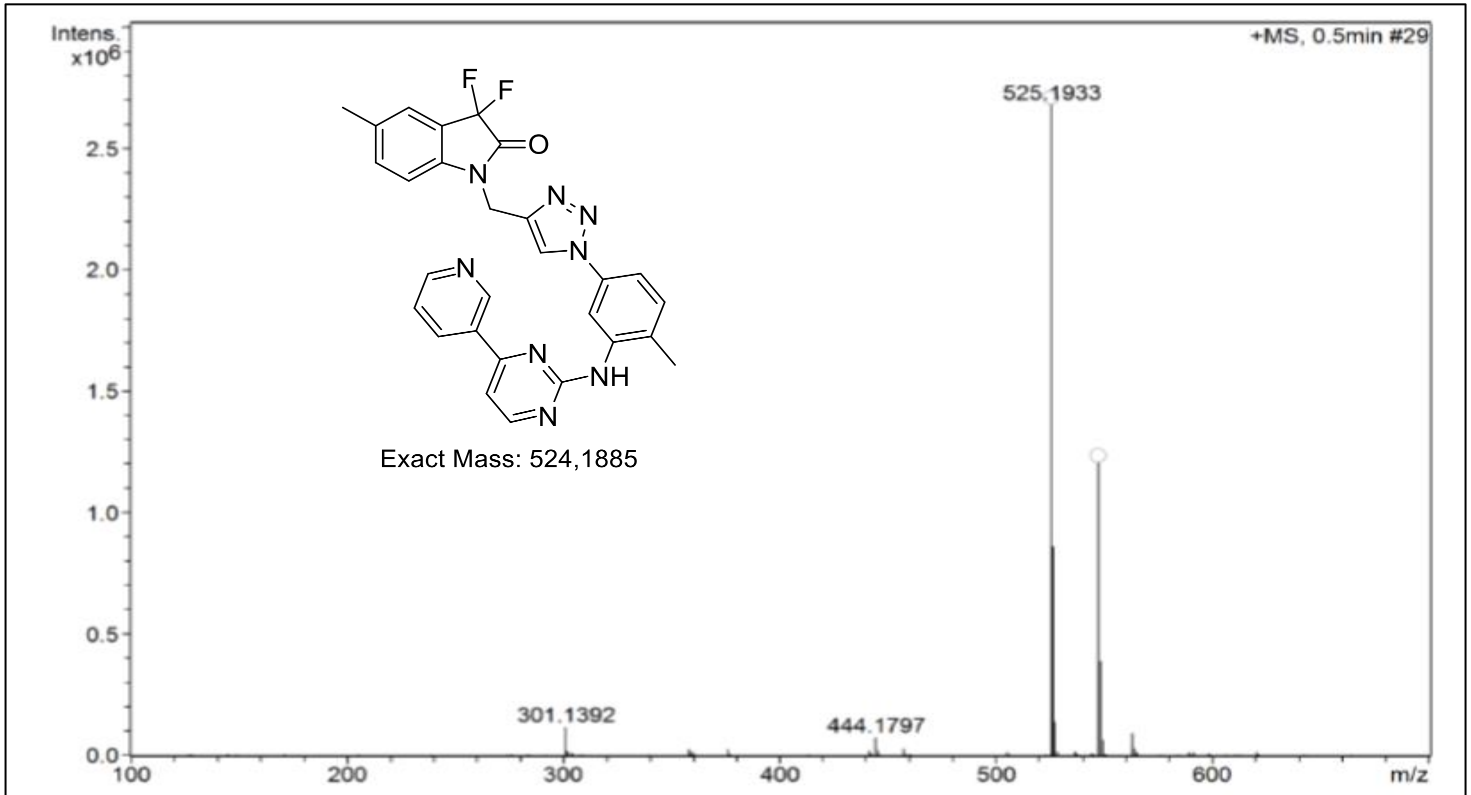

Meas. m/z	#	Ion Formula	m/z	err [ppm]	Mean err [ppm]	rdb	N-Rule	e⁻ Conf	mSigma	Std I	Std Mean m/z	Std I VarNorm	Std m/z Diff	Std Comb Dev
525.193273	1	C28H23F2N8O	525.195740	4.7	5.1	20.5	ok	even	7.9	11.3	n.a.	n.a.	n.a.	n.a.
	2	C23H23F2N10O3	525.191718	-3.0	-2.9	16.5	ok	even	19.3	27.9	n.a.	n.a.	n.a.	n.a.
	3	C22H27F2N6O7	525.190380	-5.5	-5.2	11.5	ok	even	32.6	46.4	n.a.	n.a.	n.a.	n.a.
547.175153	1	C28H22F2N8NaO	547.177685	4.6	4.9	20.5	ok	even	6.1	8.6	n.a.	n.a.	n.a.	n.a.
	2	C23H22F2N10NaO3	547.173662	-2.7	-2.8	16.5	ok	even	21.0	30.6	n.a.	n.a.	n.a.	n.a.
	3	C22H26F2N6NaO7	547.172324	-5.2	-5.0	11.5	ok	even	34.3	49.0	n.a.	n.a.	n.a.	n.a.

Figure S46. HRMS of 3,3-difluoro-5-methyl-1-((1-(4-methyl-3-((4-(pyridin-3-yl)pyrimidin-2-yl)amino)phenyl)-1H-1,2,3-triazol-4-yl)methyl)indolin-2-one (**3b**)

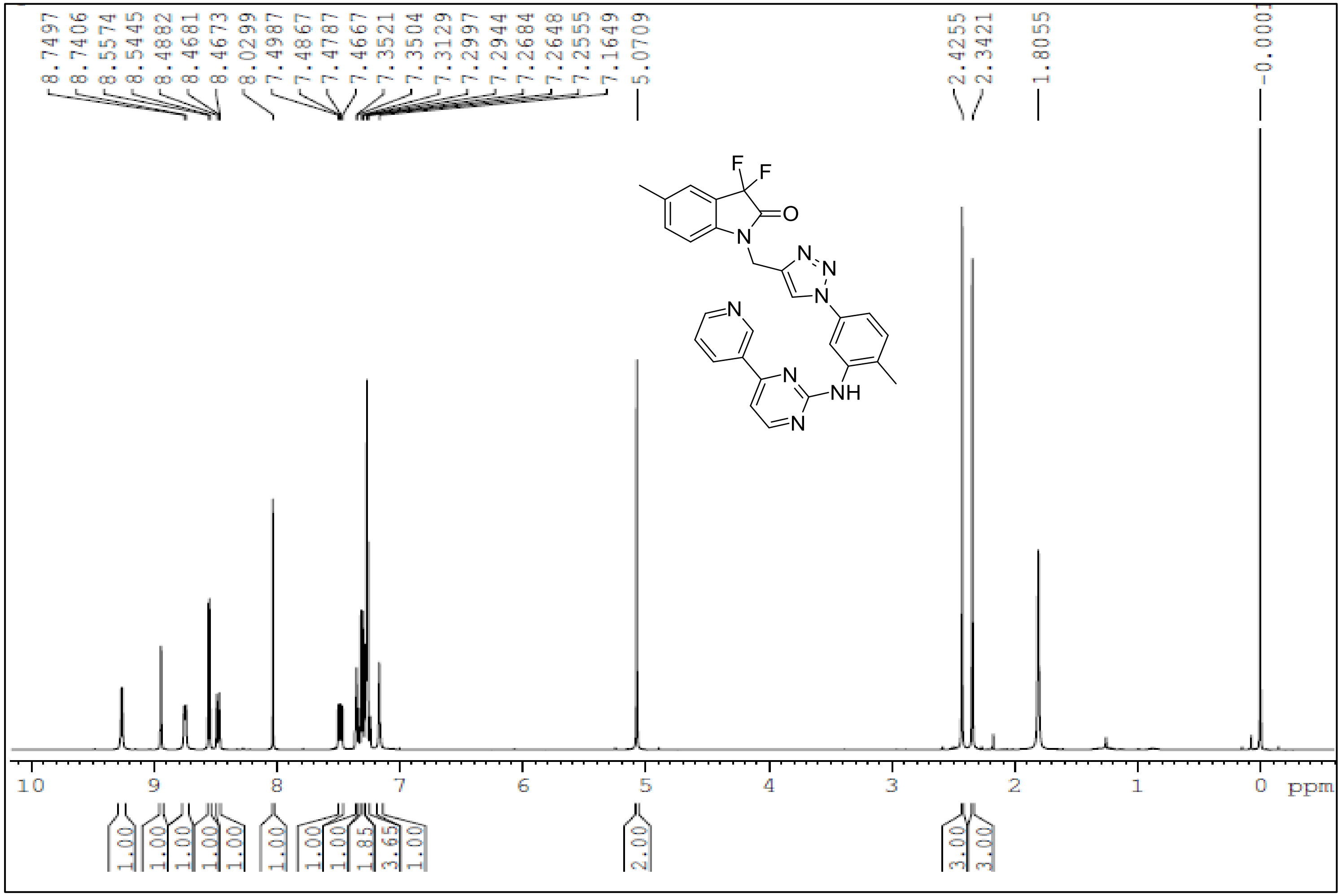

Figure S47. ^{1}H NMR of 3,3-difluoro-5-methyl-1-((1-(4-methyl-3-((4-(pyridin-3-yl)pyrimidin-2-yl)amino)phenyl)-1H-1,2,3-triazol-4-yl)methyl)indolin-2-one (**3b**)

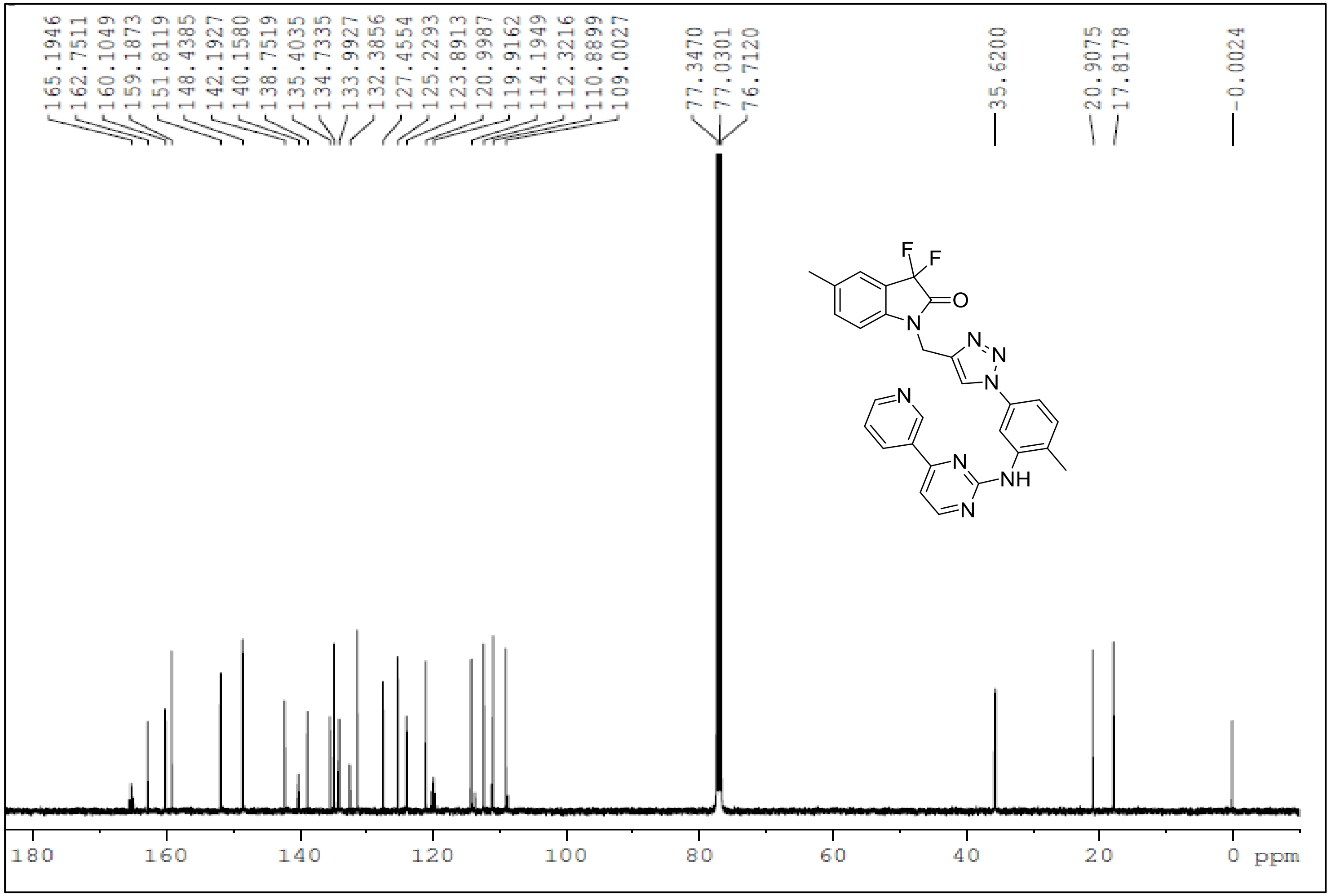

Figure S48. ^{13}C NMR of 3,3-difluoro-5-methyl-1-((1-(4-methyl-3-((4-(pyridin-3-yl)pyrimidin-2-yl)amino)phenyl)-1H-1,2,3-triazol-4-yl)methyl)indolin-2-one (**3b**)

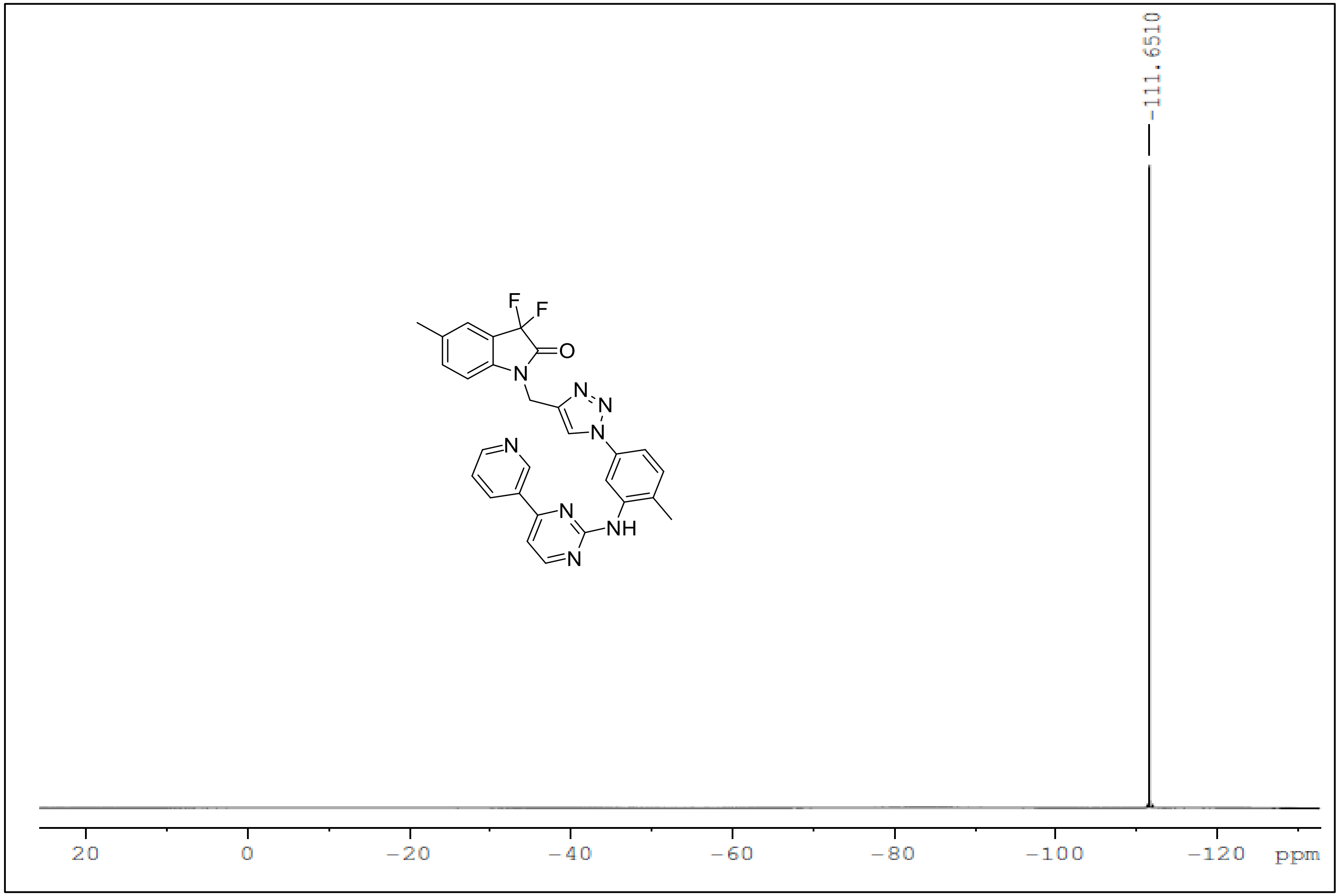

Figure S49. ^{19}F NMR of 3,3-difluoro-5-methyl-1-((1-(4-methyl-3-((4-(pyridin-3-yl)pyrimidin-2-yl)amino)phenyl)-1H-1,2,3-triazol-4-yl)methyl)indolin-2-one (**3b**)

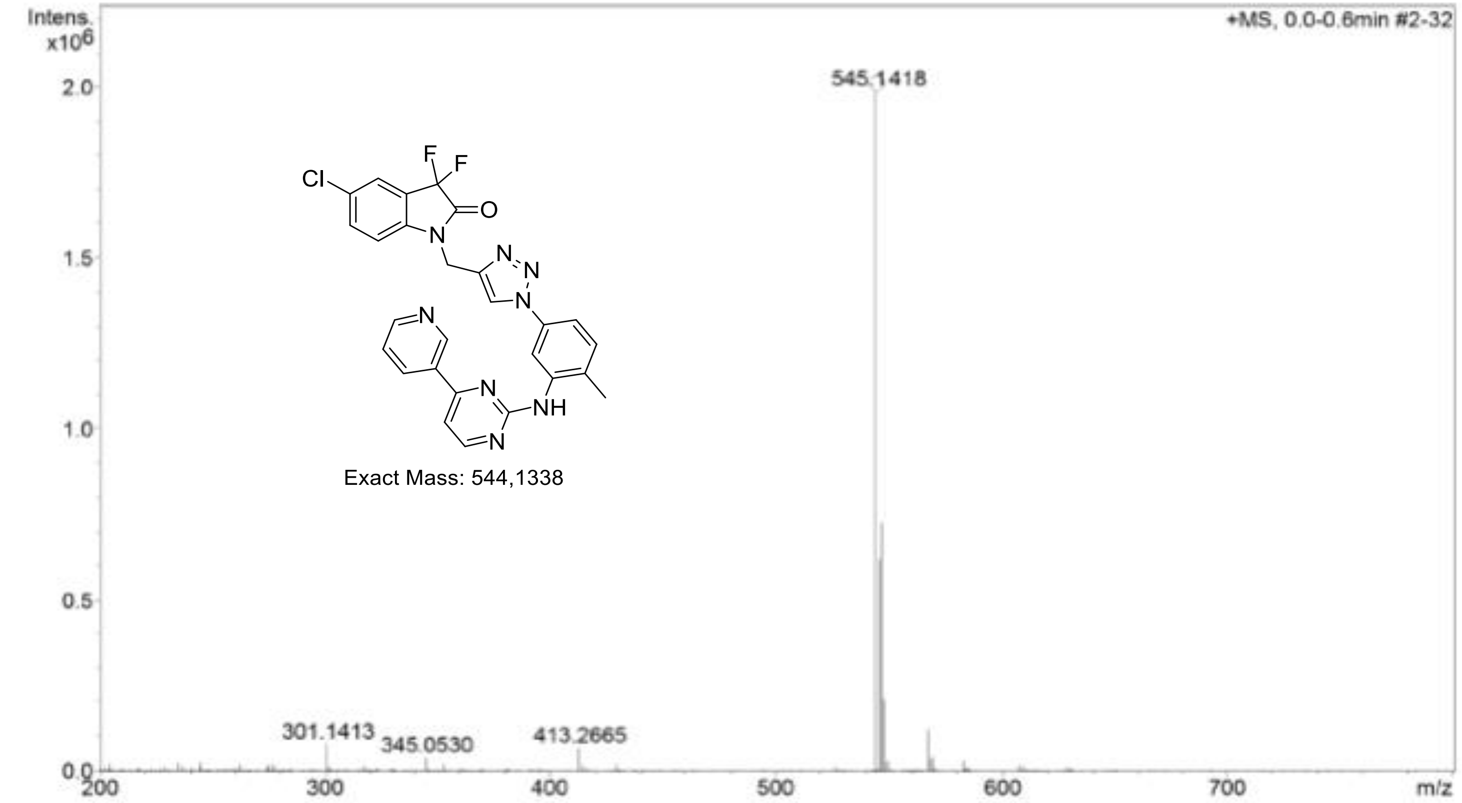

Meas. m/z	#	Ion Formula	m/z	err [ppm]	Mean err [ppm]	rdb	N-Rule	e⁻ Conf	mSigma	Std I	Std Mean m/z	Std I VarNorm	Std m/z Diff	Std Comb Dev
545.141771	1	C27H20ClF2N8O	545.141118	-1.2	-0.7	20.5	ok	even	6.1	8.2	n.a.	n.a.	n.a.	n.a.
	2	C26H24ClF2N4O5	545.139780	-3.7	-2.9	15.5	ok	even	6.7	8.4	n.a.	n.a.	n.a.	n.a.
	3	C31H24ClF2N2O3	545.143803	3.7	4.8	19.5	ok	even	18.3	24.7	n.a.	n.a.	n.a.	n.a.

Figure S50. HRMS of 5-chloro-3,3-difluoro-1-((1-(4-methyl-3-((4-(pyridin-3-yl)pyrimidin-2-yl)amino)phenyl)-1H-1,2,3-triazol-4-yl)methyl)indolin-2-one (**3c**)

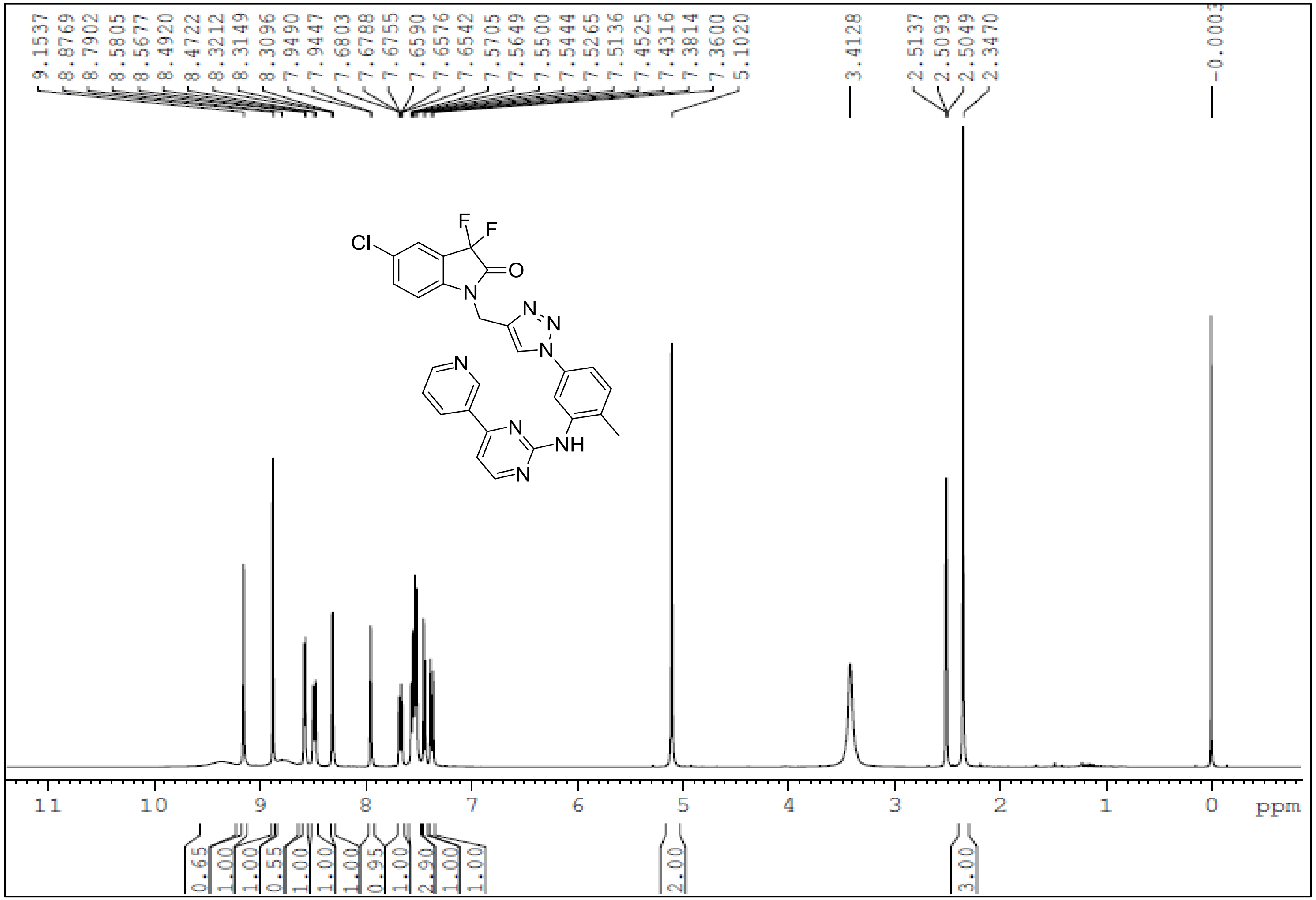

Figure S51. ¹H NMR of 5-chloro-3,3-difluoro-1-((1-(4-methyl-3-((4-(pyridin-3-yl)pyrimidin-2-yl)amino)phenyl)-1H-1,2,3-triazol-4-yl)methyl)indolin-2-one (**3c**)

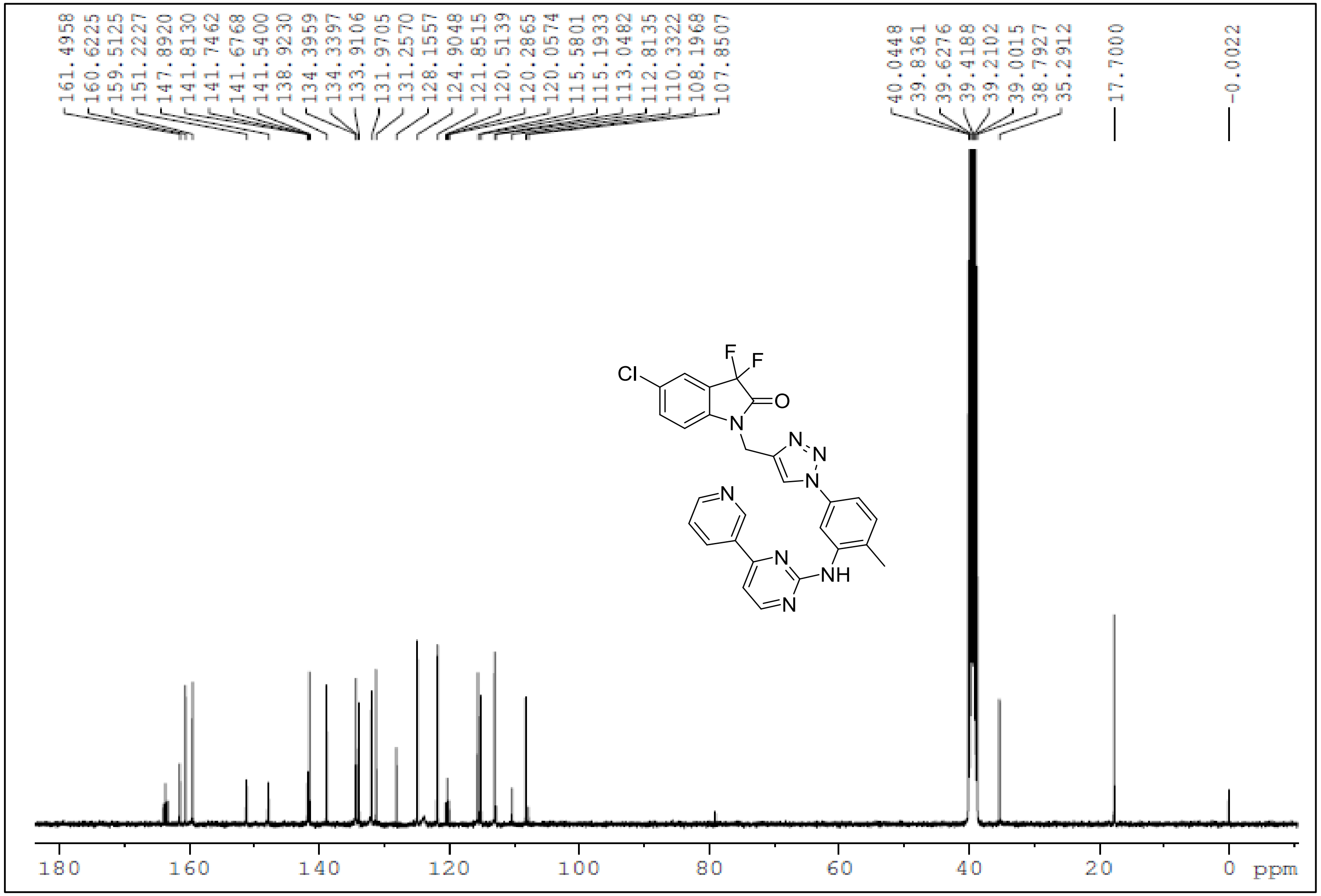

Figure S52. ^{13}C NMR of 5-chloro-3,3-difluoro-1-((1-(4-methyl-3-((4-(pyridin-3-yl)pyrimidin-2-yl)amino)phenyl)-1H-1,2,3-triazol-4-yl)methyl)indolin-2-one (**3c**)

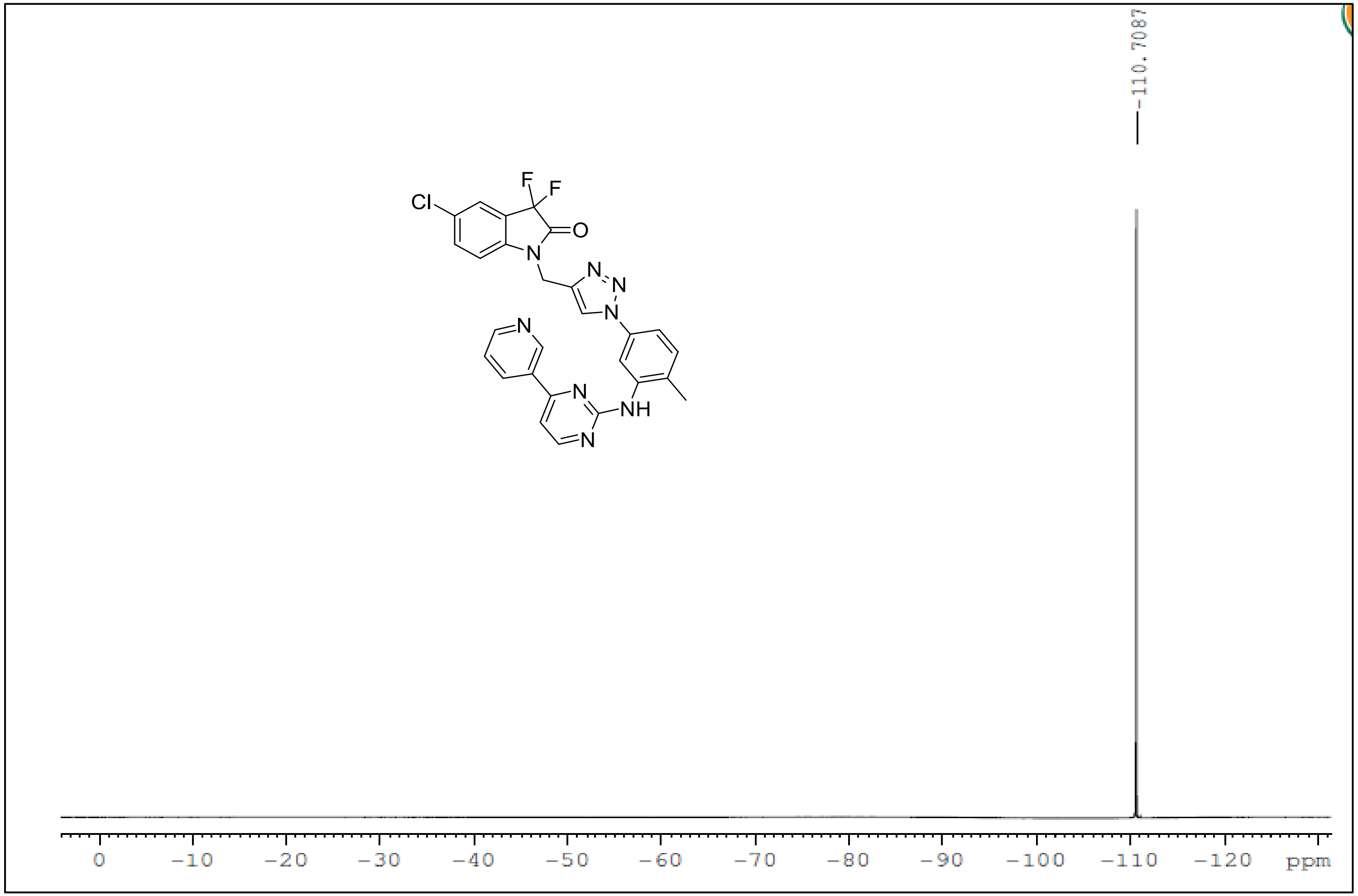

Figure S53. ^{19}F NMR of 5-chloro-3,3-difluoro-1-((1-(4-methyl-3-((4-(pyridin-3-yl)pyrimidin-2-yl)amino)phenyl)-1H-1,2,3-triazol-4-yl)methyl)indolin-2-one (**3c**)

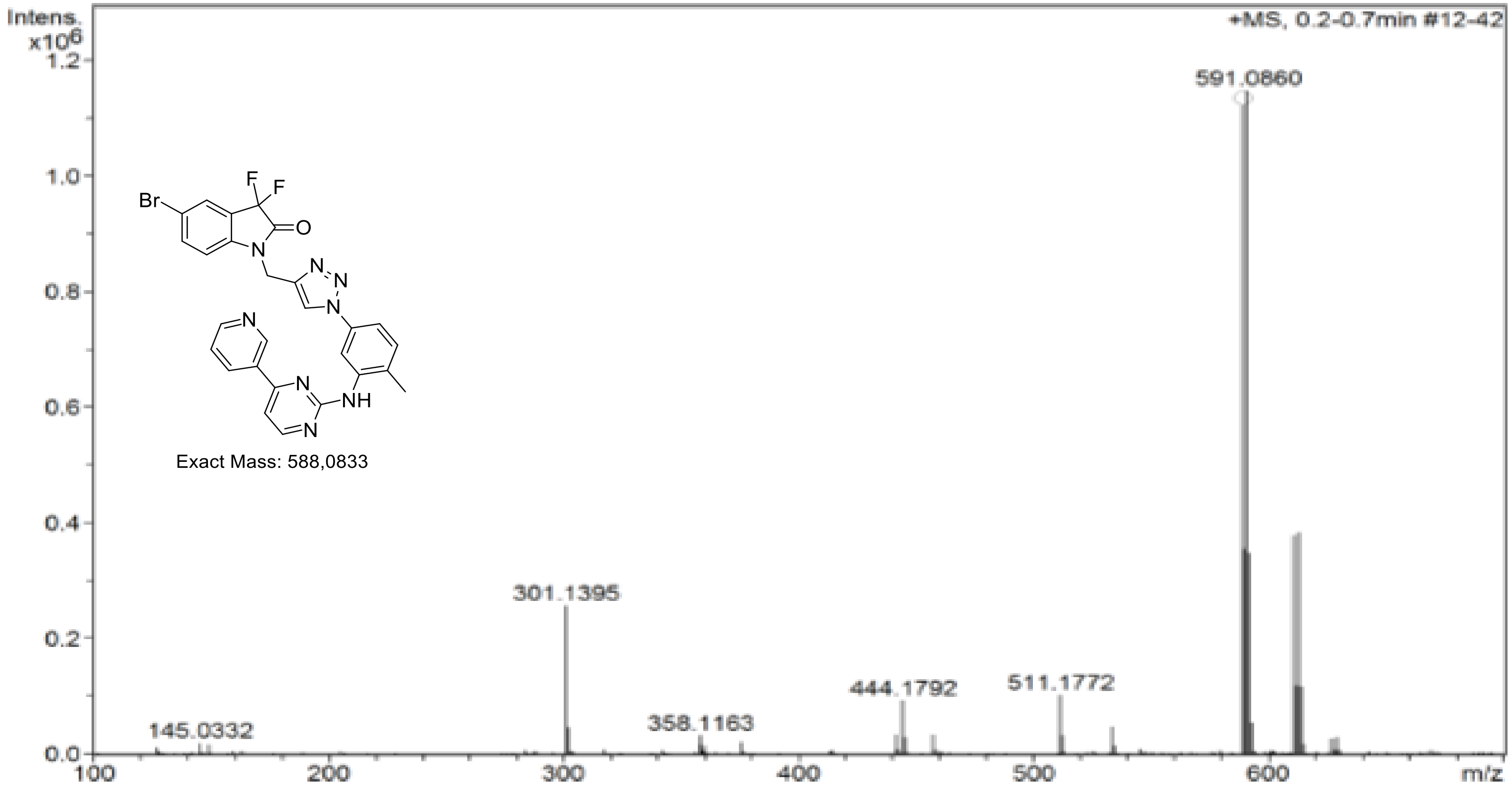

Meas. m/z	#	Ion Formula	m/z	err [ppm]	Mean err [ppm]	rdb	N-Rule	e⁻ Conf	mSigma	Std I	Std Mean m/z	Std I VarNorm	Std m/z Diff	Std Comb Dev
9.087738	1	C27H20BrF2N8O	589.090602	4.9	5.1	20.5	ok	even	5.0	4.9	n.a.	n.a.	n.a.	n.a.
	2	C26H24BrF2N4O5	589.089265	2.6	3.0	15.5	ok	even	10.0	8.5	n.a.	n.a.	n.a.	n.a.
	3	C22H20BrF2N10O3	589.086580	-2.0	-2.0	16.5	ok	even	22.5	22.0	n.a.	n.a.	n.a.	n.a.
	4	C25H28BrF2O9	589.087927	0.3	1.0	10.5	ok	even	24.0	21.4	n.a.	n.a.	n.a.	n.a.

Figure S54. HRMS of 5-bromo-3,3-difluoro-1-((1-(4-methyl-3-((4-(pyridin-3-yl)pyrimidin-2-yl)amino)phenyl)-1H-1,2,3-triazol-4-yl)methyl)indolin-2-one (**3d**)

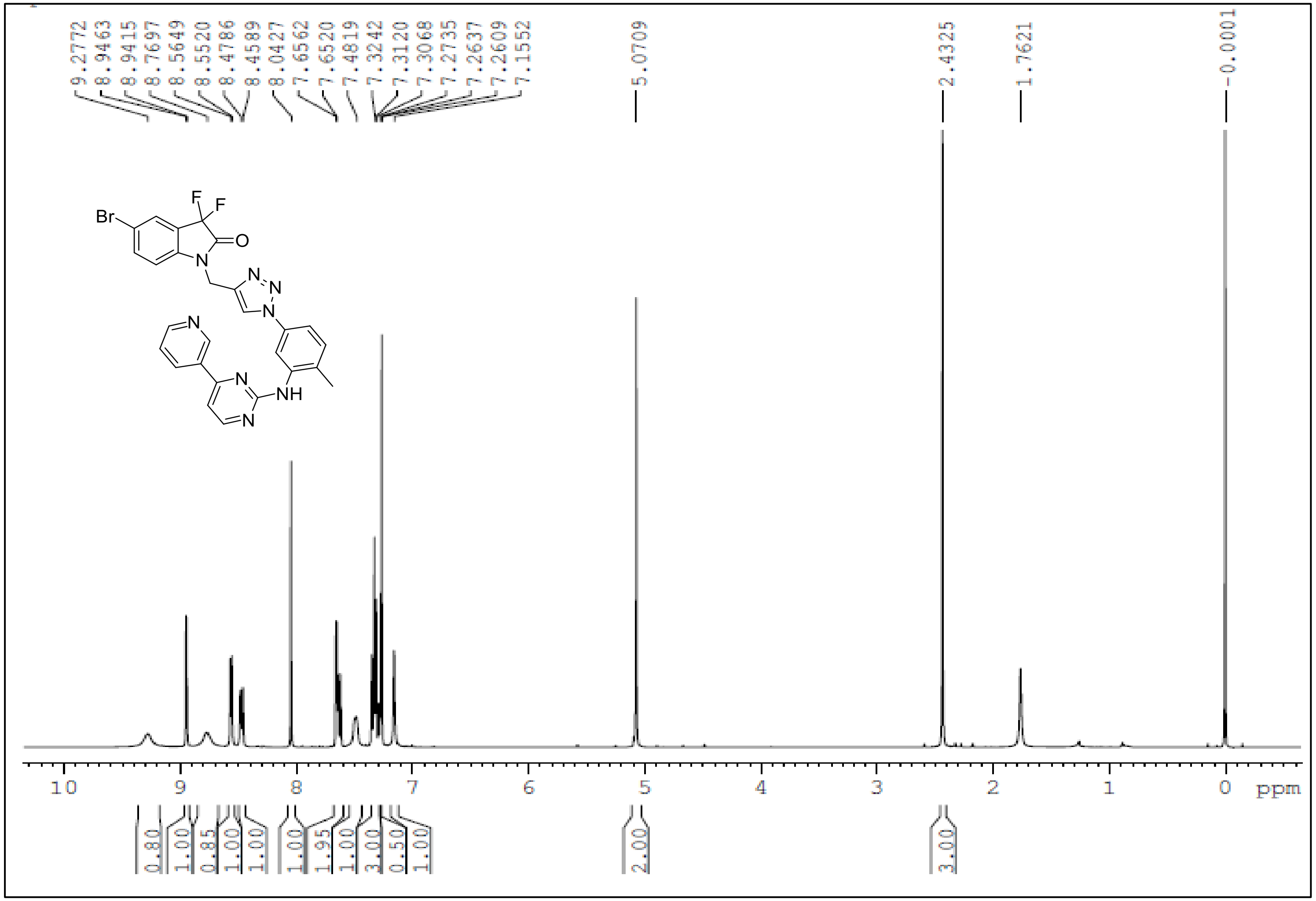

Figure S55. ^{1}H NMR of 5-bromo-3,3-difluoro-1-((1-(4-methyl-3-((4-(pyridin-3-yl)pyrimidin-2-yl)amino)phenyl)-1H-1,2,3-triazol-4-yl)methyl)indolin-2-one (**3d**)

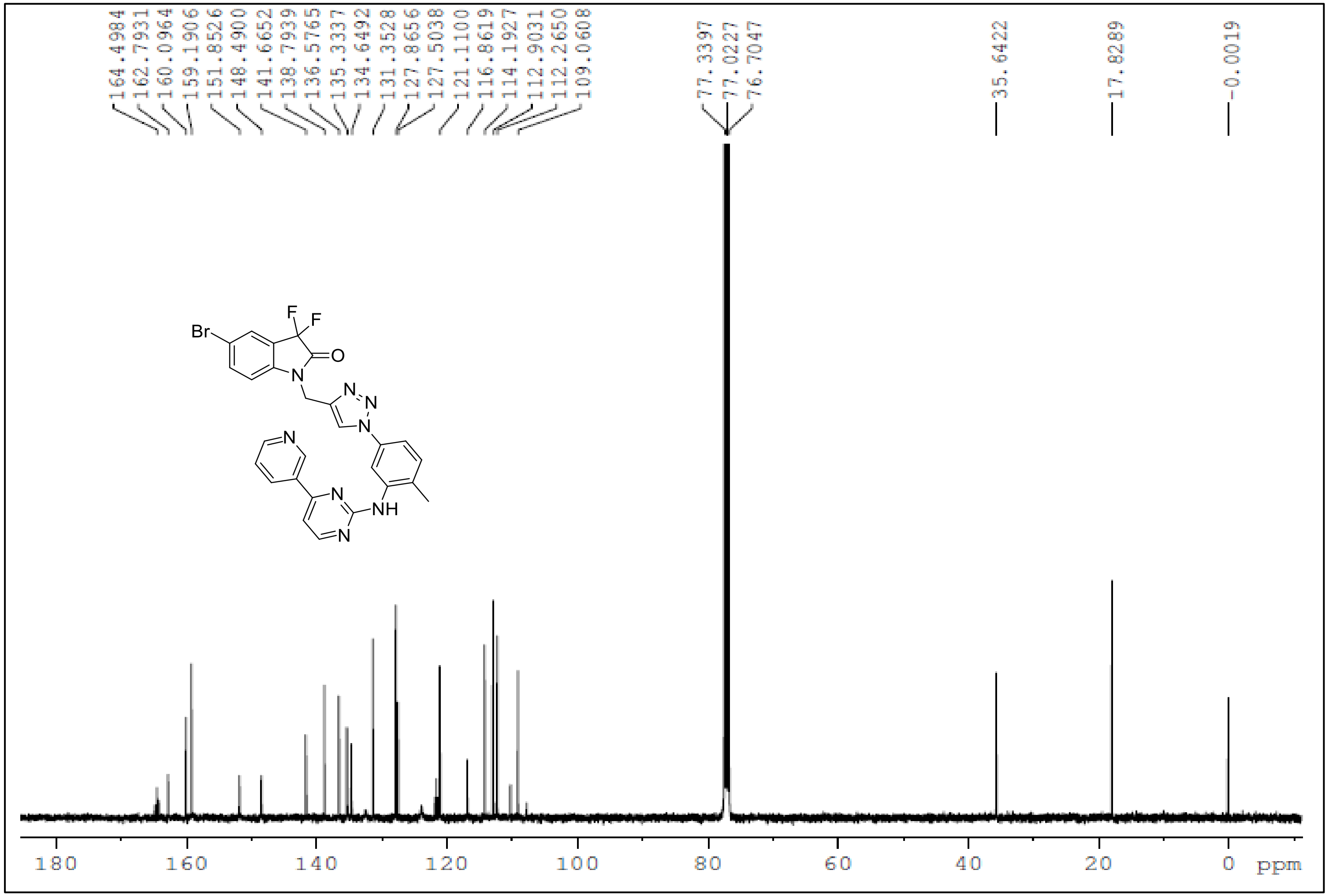

Figure S56. ^{13}C NMR of 5-bromo-3,3-difluoro-1-((1-(4-methyl-3-((4-(pyridin-3-yl)pyrimidin-2-yl)amino)phenyl)-1H-1,2,3-triazol-4-yl)methyl)indolin-2-one (**3d**)

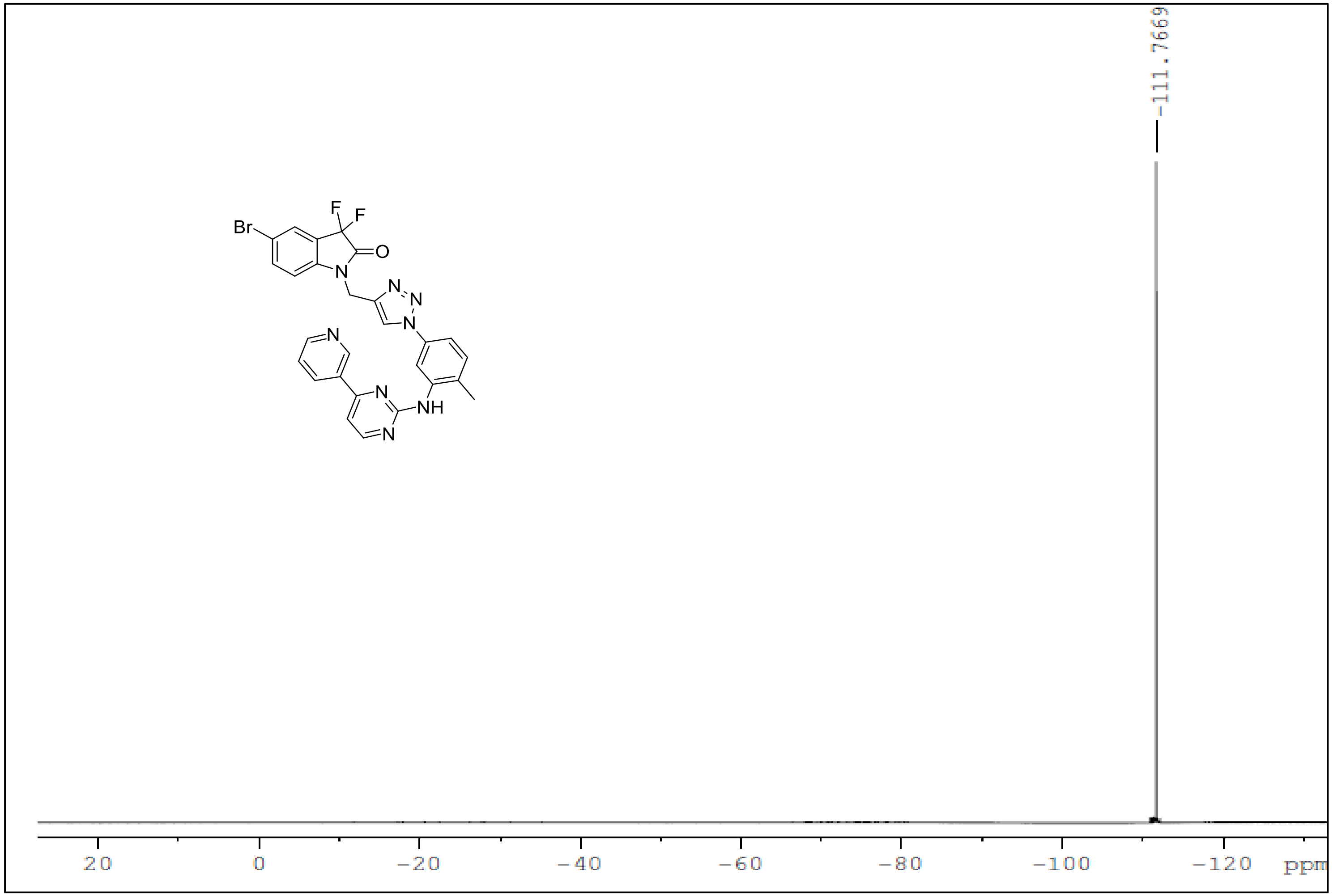

Figure S57. ^{19}F NMR of 5-bromo-3,3-difluoro-1-((1-(4-methyl-3-((4-(pyridin-3-yl)pyrimidin-2-yl)amino)phenyl)-1H-1,2,3-triazol-4-yl)methyl)indolin-2-one (**3d**)

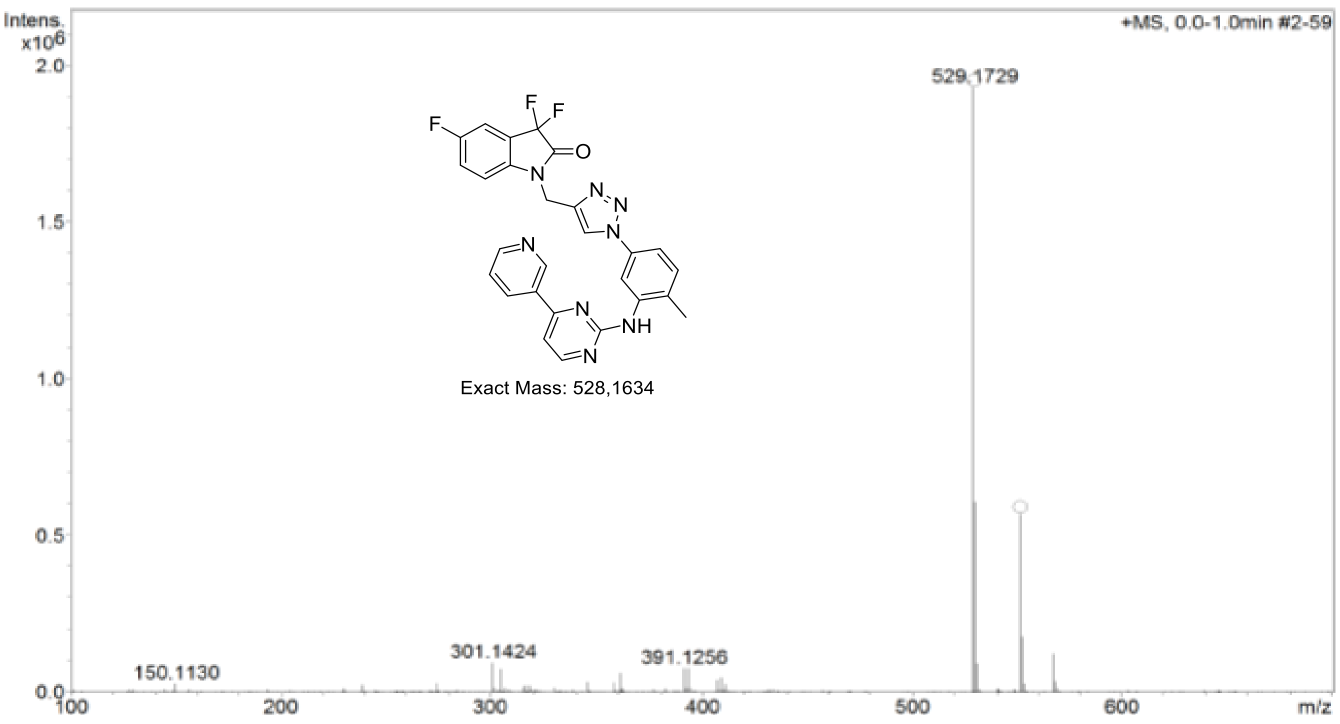

Meas. m/z	#	Ion Formula	m/z	err [ppm]	Mean err [ppm]	rdb	N-Rule	e^- Conf	mSigma	Std I	Std Mean m/z	Std I VarNorm	Std m/z Diff	Std Comb Dev
529.172898	1	C27H20F3N8O	529.170668	-4.2	-3.8	20.5	ok	even	6.0	9.3	n.a.	n.a.	n.a.	n.a.
	2	C26H24F3N4O5	529.169331	-6.7	-5.9	15.5	ok	even	7.9	11.2	n.a.	n.a.	n.a.	n.a.
	3	C31H24F3N2O3	529.173354	0.9	2.2	19.5	ok	even	17.9	31.7	n.a.	n.a.	n.a.	n.a.
551.154870	1	C26H23F3N4NaO5	551.151275	-6.5	-5.3	15.5	ok	even	6.1	8.7	n.a.	n.a.	n.a.	n.a.
	2	C27H19F3N8NaO	551.152613	-4.1	-3.4	20.5	ok	even	7.7	11.4	n.a.	n.a.	n.a.	n.a.
	3	C31H23F3N2NaO3	551.155298	0.8	2.5	19.5	ok	even	19.4	33.8	n.a.	n.a.	n.a.	n.a.

Figure S58. HRMS of 3,3,5-trifluoro-1-((1-(4-methyl-3-((4-(pyridin-3-yl)pyrimidin-2-yl)amino)phenyl)-1H-1,2,3-triazol-4-yl)methyl)indolin-2-one (**3e**)

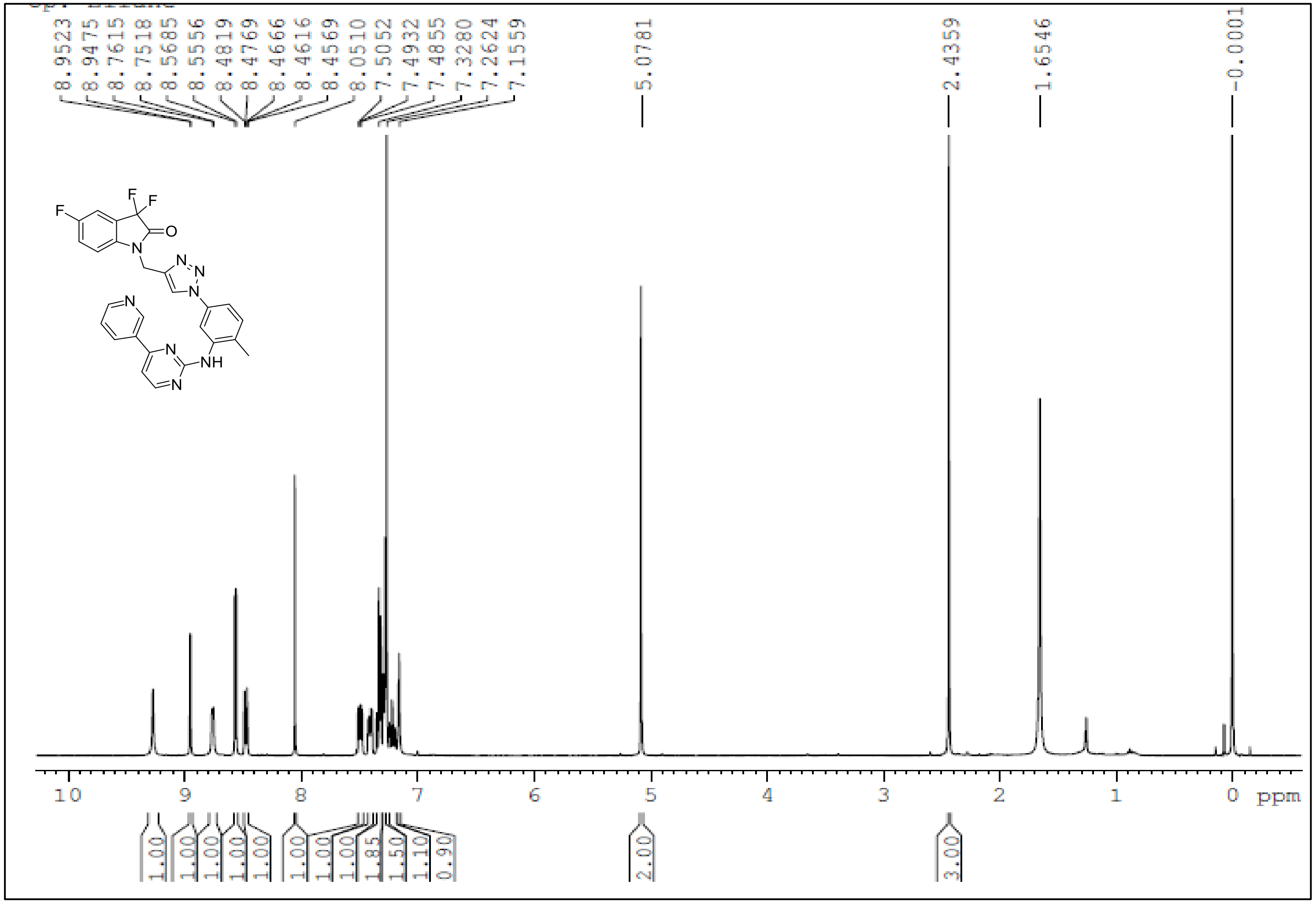

Figure S59. ^{1}H NMR of 3,3,5-trifluoro-1-((1-(4-methyl-3-((4-(pyridin-3-yl)pyrimidin-2-yl)amino)phenyl)-1H-1,2,3-triazol-4-yl)methyl)indolin-2-one (**3e**)

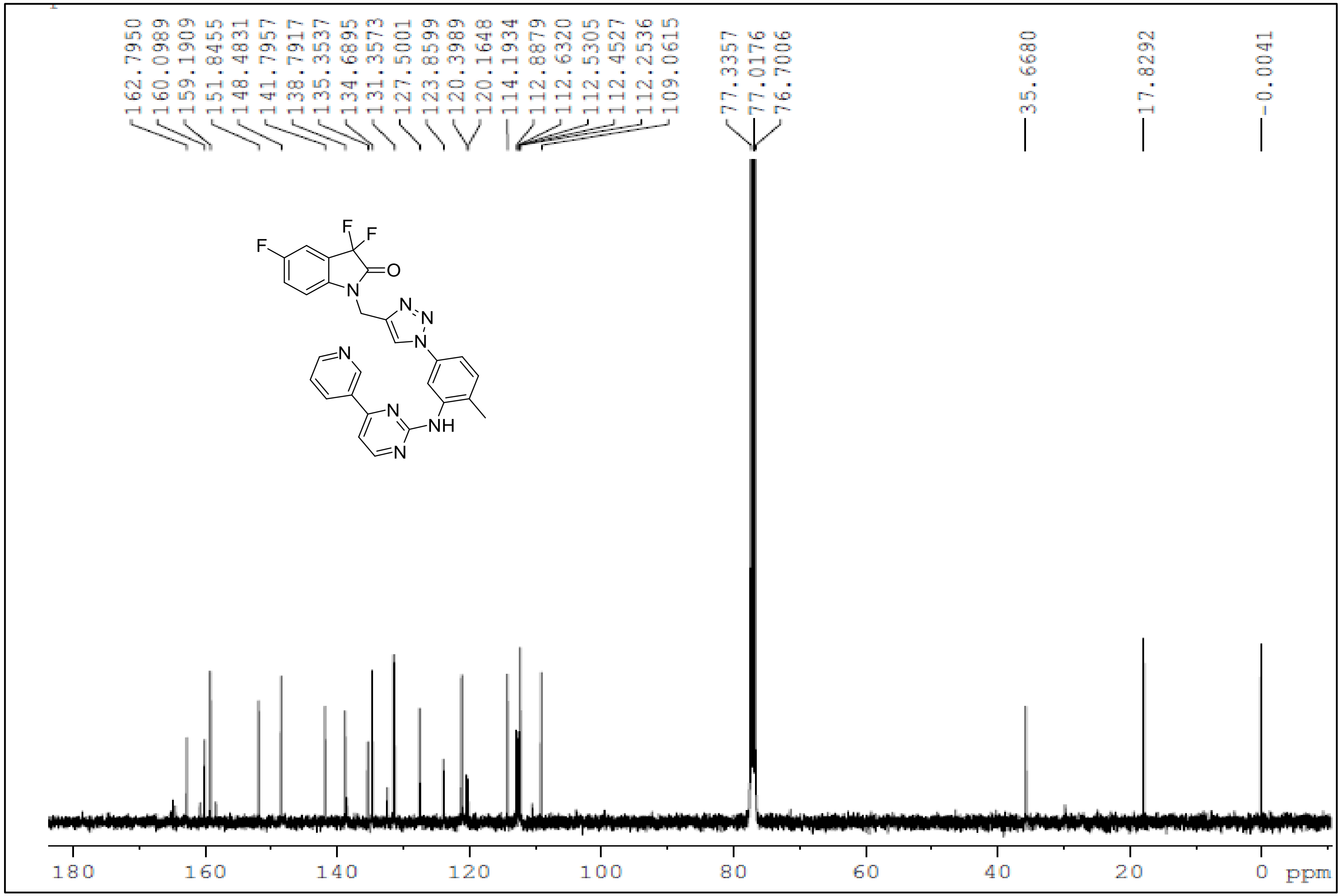

Figure S60. ^{13}C NMR of 3,3,5-trifluoro-1-((1-(4-methyl-3-((4-(pyridin-3-yl)pyrimidin-2-yl)amino)phenyl)-1H-1,2,3-triazol-4-yl)methyl)indolin-2-one (**3e**)

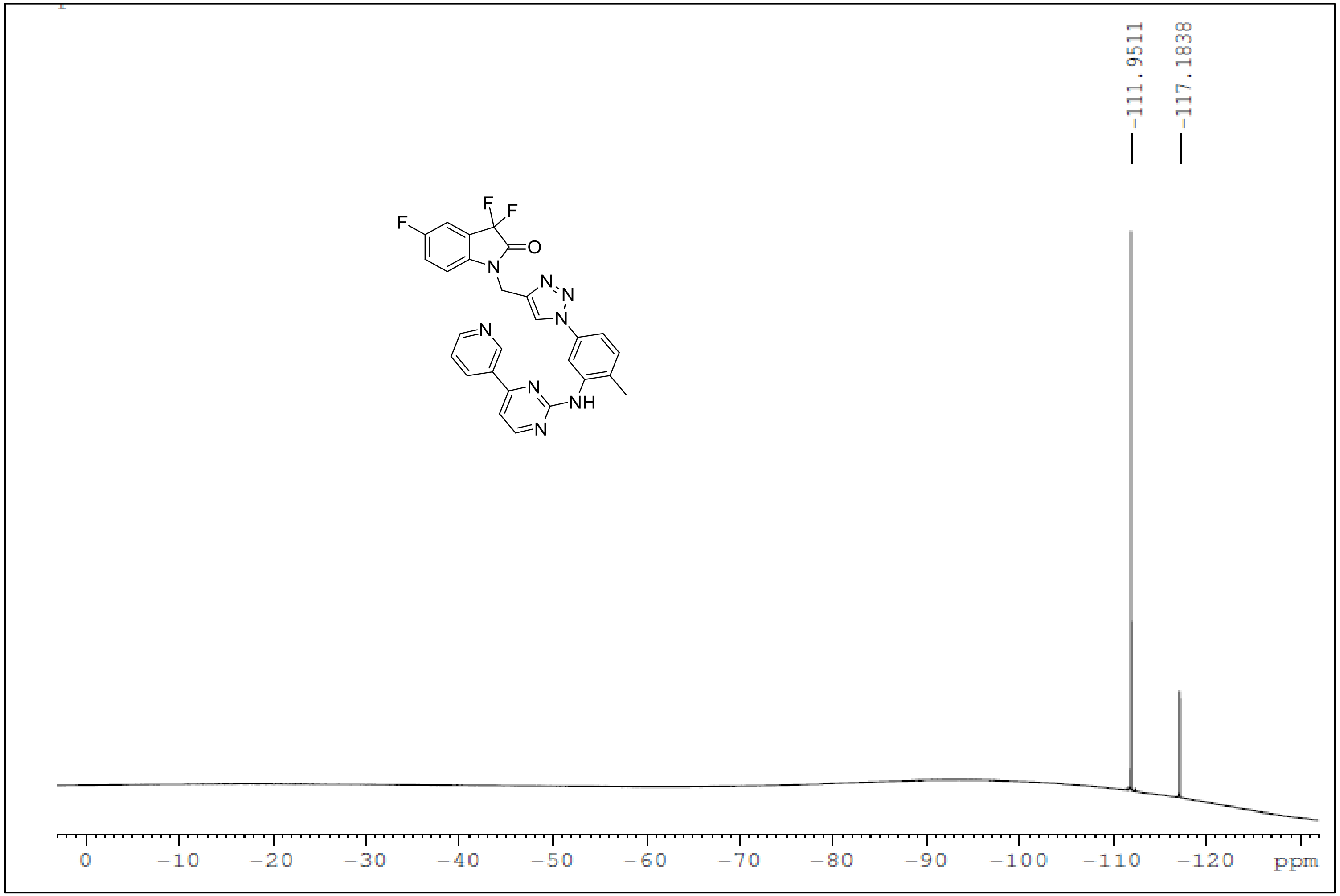

Figure S61. ^{19}F NMR of 3,3,5-trifluoro-1-((1-(4-methyl-3-((4-(pyridin-3-yl)pyrimidin-2-yl)amino)phenyl)-1H-1,2,3-triazol-4-yl)methyl)indolin-2-one (**3e**)
